# The Closing-the-Gap Effect: Joint Evaluation Leads Donors to Help Charities Farther from Their Goal

**DOI:** 10.1177/00222437241270225

**Published:** 2024-10-30

**Authors:** Rishad Habib, David J. Hardisty, Katherine White, Baek Jung Kim

**Keywords:** crowdfunding, evaluation mode, joint evaluation, goal gradient hypothesis, charitable giving

## Abstract

Charitable donations can be influenced by the level of progress a cause has made toward its fundraising goal. The current work demonstrates how jointly considering more than one charitable cause along with their goal progress information shifts consumers’ donation decisions. When charitable causes are evaluated jointly (vs. separately), the comparison makes relative need for help more salient and easier to evaluate, leading to greater giving to the cause farther from its goal. A multimethod investigation, involving six preregistered experimental studies, seven supplemental studies, and a large secondary dataset with over 10,000 projects from a micro-crowdfunding platform, provides evidence for this phenomenon and demonstrates that it is robust to variations in the type of cause, the number of projects, and the donor being able to personally complete the goal. Conversely, the effect is eliminated or reversed when charities are evaluated separately (as relative need for help is less salient), when the gap between charities is smaller (as perceptions of relative need for help are diminished), or when for-profit businesses are evaluated (as the context does not heighten sensitivity to need). This work contributes to research on goal progress and evaluation mode and has implications for charitable giving in comparative contexts like crowdfunding.

Imagine navigating a fundraising website, looking at charitable causes to potentially donate to, when you come across two similar charities that resonate with you. You note that one is closer to its goal and has raised 90% of its target, while the other is farther from its goal and has raised only 10% of its target. To which project would you donate? This research investigates how people make donation decisions when they concurrently encounter one or more charitable projects at varying distances from their goals. Previous work suggests that, under some circumstances, people tend to give more to causes that are closer to their goal (Cryder, Loewenstein, and Seltman 2013; Kivetz, Urminsky, and Zheng 2006), and, in others, they give more to those farther from their goal (Huang, Zhang, and Broniarczyk 2012; Koo and Fishbach 2012). We build on this existing work to demonstrate an important factor that influences when consumers will give less versus more to a charity as a function of its goal progress. Specifically, we predict and find that the evaluation mode matters: In joint evaluation (vs. separate evaluation) contexts, consumers tend to give more to the charity that is farther from its goal.

We suggest that consumers will be most likely to help an organization farther from its goal when (1) the domain is relatively prosocial, such as in charitable fundraising contexts, and (2) when the evaluation is comparative, wherein more than one charity is considered by the potential donor. This is because the effect emerges when the context heightens sensitivity to need for help (i.e., the context is somewhat prosocial) and when the relative need for help is more salient and easier to evaluate (i.e., in joint evaluation). Across six preregistered main experimental studies, seven supplemental studies, and one study using secondary real-world data, we show that when individuals evaluate charities jointly, they tend to be more likely to donate to (and donate larger amounts to) the charitable organization that is farther from its goal. This effect is eliminated or reversed when relative need is less salient and harder to evaluate, such as when organizations are presented alone; when the context does not heighten sensitivity to need, such as fundraising for a for-profit business rather than for a charity; or when perceptions of need are weakened, such as when the gap between the charities is smaller.

We build on previous work investigating charitable giving and make several focal contributions. First, we build on the goal progress literature. Previous research on goal progress demonstrates greater motivation to reach a goal at both low and high levels of progress depending on the inferences consumers make. For instance, the goal gradient effect demonstrates that people make greater effort and progress toward a goal when an organization or individual is closer to reaching it (Cryder, Loewenstein, and Seltman 2013; Kivetz, Urminsky, and Zheng 2006). In contrast, other work shows that as people make progress toward a goal, they will sometimes switch to conflicting goals with lower progress (Fishbach and Dhar 2005) or put in more effort when their attention is directed toward a domain with less progress, altering goal representation in consumers’ minds (Huang, Zhang, and Broniarczyk 2012; Koo and Fishbach 2012). We add to this literature by uncovering a novel, previously untested factor that reliably shifts preferences toward the lower progress option. Specifically, we show that when charitable causes are evaluated jointly (vs. separately), people are more likely to support the option that is farther from its goal. Our research thus contributes to work on balancing multiple goals (Fishbach and Dhar 2005), inference making in goal research (Fishbach, Henderson, and Koo 2011), and representations of goal pursuit (Huang, Zhang, and Broniarczyk 2012; Koo and Fishbach 2012).

Second, we identify the mechanism by which goal progress impacts donations under joint evaluation. We propose that charitable donors are generally sensitive to need and want to give to those with greater need for help. In the context of a prosocial cause fundraising toward a target goal (e.g., “This charity has raised $200 toward a goal of $5,000”), the charity's need for help, that is, the potential donor's perception of the level of assistance the target requires to reach the goal, becomes the main concern for givers. Our proposed mechanism is distinct from previous work in the goal progress literature as it focuses on perceptions of the target rather than the focal actor's affective state and satisfaction with goal progress (Fishbach, Henderson, and Koo 2011).

Third, we build on existing work on preference shifts in joint versus separate evaluation, more generally (Hsee et al. 1999). We argue that the comparison in joint presentation makes a key attribute (need for help) easier to evaluate, and also changes perceptions of that attribute. While there is an inverse relationship between relative fundraising progress and perceived need for help, consumers may not always be able to assess this accurately. Joint evaluation makes relative need salient and easier to evaluate, widening the gap in perceptions of need for help between the charities in joint evaluation, compared with separate evaluation. We show how this change in perceptions contributes to preference shifts in joint versus separate evaluation and makes participants more likely to give on the basis of need for help.

Fourth, we identify theoretically relevant moderators of the closing-the-gap effect. We argue that consumers have increased sensitivity to need for help in prosocial contexts where they are focused on benefits to others (Bhattacharjee, Dana, and Baron 2017). We show that our effect is stronger when considering prosocial causes such as nonprofits and is eliminated when considering for-profit organizations, where consumers are less sensitive to the needs of others (Aaker, Vohs, and Mogilner 2010). We also argue that the size of the gap in terms of differences in goal progress between causes serves as an indicator of relative need for help. In support of this, we find that the effect is diminished when the size of the gap between charities closer to versus farther from their goal is smaller.

Finally, from a practical perspective, this work demonstrates an easy-to-implement solution to direct funds where they are most needed through a simple change in presentation format for online fundraising platforms, email communications, or other media. Our research suggests that charitable marketers can effectively use comparative messaging and advertising to drive charitable giving to projects in their initial stages of fundraising or to projects that are struggling to raise funds. We next turn to a discussion of the theoretical background.

## Conceptual Development

Crowdfunding websites aim to raise small amounts of money from a large number of people and are increasingly used by charities and individuals when fundraising toward a goal. For example, GoFundMe has helped raise over $9 billion from more than 120 million donations (GoFundMe 2024), and Kiva has lent out $12 billion in zero-interest loans to 5 million recipients (Kiva 2024).

For charities, this new crowdfunding landscape brings two key changes from prior methods of fundraising that are relevant to the current research. First, crowdfunding has made it much easier for donors to compare and evaluate multiple charitable projects simultaneously. This differs from traditional forms of charitable marketing where potential donors often receive direct marketing communications via mail, phone, or email, focusing their attention on a single target organization trying to raise funds. Second, consumers now often receive real-time, up-to-date information about an organization's progress, such as how much money has been raised and how far an organization is from its goal. This gives consumers additional information to use when deciding whether and how much to give to a charitable cause.

We investigate the potential consequences of these changes in evaluation mode (i.e., separate vs. joint evaluation) and availability of information about goal progress on consumers’ charitable decision-making. Specifically, we examine how consumers choose to allocate charitable support when they evaluate options together versus on their own and have information about each charity's progress toward its goal. We predict and demonstrate that, in joint evaluation, when consumers are explicitly shown two or more charitable projects at different levels of goal progress, they tend to donate more to the one that is farther from its goal. Furthermore, we predict and find that this effect is driven by the change in perceptions of the organization's need for help; donors want to give to charities that need more assistance to meet their goals. In this case, jointly evaluating two charities makes relative need for help more salient and easier to evaluate, leading consumers to donate based on need for help. However, it is notable that the prediction that low goal progress will lead to increased donations is counter to what some previous literature would suggest. Next, we discuss competing predictions about the effects of low goal progress on charitable giving, and we outline the conditions under which low goal progress will have positive effects on donations.

### Goal Pursuit at Different Distances from the Goal

Research on the goal gradient effect suggests that consumers will exhibit greater donations to an organization closer to (vs. farther from) reaching its goal. Specifically, this work predicts that the “tendency to approach a goal increases with proximity to the goal” (Kivetz, Urminsky, and Zheng 2006, p. 39) and has been found in a variety of behavioral, consumption, and marketing contexts (Cryder, Loewenstein, and Seltman 2013; Kivetz, Urminsky, and Zheng 2006). Relevant to the present research, the goal gradient effect has been shown to extend beyond achieving one's own goals to helping others reach their goals, in contexts such as giving to crowdfunding projects (Dai and Zhang 2019; Kuppuswamy and Bayus 2018) or donating to charity (Cryder, Loewenstein, and Seltman 2013; Jensen, King, and Carcioppolo 2013).

In contrast to the goal gradient effect, another line of work shows that, under certain conditions, subsequent goal pursuit can *decelerate* as one gets closer to achieving a goal. The effect of higher progress on goal pursuit can depend on the inferences consumers make; when they infer sufficient progress has been made, they may decrease effort and future goal pursuit (Fishbach, Henderson, and Koo 2011; Koo and Fishbach 2008). For instance, when consumers hold multiple conflicting goals (e.g., weight loss and eating high-calorie food), higher perceived progress in one area can lead to a preference for goal-inconsistent rather than goal-consistent options (Fishbach and Dhar 2005), and focusing on one goal may devalue others (Brendl, Markman, and Messner 2003). At lower levels of progress, consumers are more concerned about whether the goal is attainable and put in more effort when the velocity of progress is high than when they are at higher levels of progress (Huang and Zhang 2011). Similarly, when consumers are already committed to a goal, highlighting remaining progress at lower levels of progress can increase goal pursuit (Koo and Fishbach 2012). Taken together, this body of work predicts and finds the opposite pattern of results from the goal gradient effect.

### Goal Progress and Evaluation Mode

We introduce a novel factor that determines how consumers will respond to a charity's fundraising progress: evaluation mode. We predict that when charities are considered jointly, the charity farther from its goal will receive relatively higher donations than the one closer to its goal. More broadly, joint evaluation (where two or more options are presented simultaneously) versus separate evaluation (where each option is presented on its own) is one of the most widely discussed distinctions in evaluation modes (Bazerman et al. 1999; Hsee 1996). Yet, past work on goal progress has focused almost exclusively on separate evaluation, in which individuals evaluate their own or another's progress in isolation. Whereas normative decision theories suggest that people should have stable and consistent preferences, regardless of how such preferences are elicited, research on evaluation modes demonstrates that choice format can have a large influence on decisions such as payoff allocations, willingness to pay, and consumer preferences and choices (Bazerman, Loewenstein, and White 1992; Hsee 1996; Irwin et al. 1993; Nowlis and Simonson 1997).

One of the main theories advanced to explain preference shifts across evaluation mode, wherein the option that was preferred in one mode becomes less desirable in the other, is the *evaluability* hypothesis (Hsee 1996). This posits that preference shifts occur when one attribute becomes easier to evaluate when options are presented jointly versus separately, which leads to differences when responding (Hsee 1996; Li and Hsee 2019). Consumers draw inferences from the information they are given (Koo and Fishbach 2008), and joint evaluation can systematically change the inferences they make by changing how easy it is to evaluate important attributes. We draw on this work to predict that the key attribute of need for help will be more salient and easier to evaluate in joint (vs. separate) evaluation mode and that this will increase perceptions of need for help for the charity farther from its goal, leading to greater donations to the charity with a greater need for help. We conceptualize *need for help* as the potential donor's perception of the level of assistance that the target (such as the recipient or the cause) requires. This focus on the target distinguishes need for help from related constructs, such as *need for progress*, that focus on the psychological motivation of the donor (Fishbach, Henderson, and Koo 2011; Fishbach, Koo, and Finkelstein 2014; Koo and Fishbach 2008, 2012). We suggest that, when information is presented in a way that emphasizes the need for help of a target charity, this will drive donations to that target. Given that relative (vs. absolute) judgments have been shown to make various dimensions including social status, wealth, and pay (Major and Forcey 1985; Ordabayeva and Chandon 2011) easier to evaluate, we propose that comparative evaluation of goal progress should make it easier to evaluate differences in need for help between options, leading consumers to give to the project that has the greater perceived need for help (Bazerman et al. 1994; Major and Forcey 1985). We dub this the “closing-the-gap” effect.

In sum, we argue that joint evaluation substantially alters consumers’ decision-making in charitable giving contexts, as it makes relative need for help more salient and easier to evaluate. Thus, we predict that evaluation mode will moderate the effect of goal progress on donations such that joint evaluation will shift donations away from the charity that is closer to its goal (i.e., lower need for help) and toward the charity that is farther from its goal (i.e., higher need for help). More formally:

**H_1_:** Evaluation mode influences donations to charities such that, compared with separate evaluation, joint evaluation results in greater giving to charities farther from their goal.
**H_2_:** Perceptions of need for help mediate the relationship between relative distance from goal and donations to charity.

### The Role of Prosocial Contexts

Our conceptualization proposes that the closing-the-gap effect emerges under conditions where consumers are sensitive to need. Sensitivity toward need for help is likely heightened in contexts that are relatively more prosocial in nature, where the focus is more on helping others as opposed to generating profit. We propose that sensitivity to need for help is likely more pronounced in prosocial contexts, such as charitable giving, where consumers are focused on benefits to others (Bhattacharjee, Dana, and Baron 2017). In support of this proposition, research demonstrates that helping those in need is a core moral rationale for engaging in charitable giving (Cryder, Botti, and Simonyan 2017) and that “need” is a key reason for people's decision to donate to a charitable cause (Breeze 2013). Conversely, when for-profit intentions are focal, consumers tend to neglect other-focused benefits (Bhattacharjee, Dana, and Baron 2017) and are less sensitive to the needs of others (Aaker, Vohs, and Mogilner 2010). Therefore, we propose that organization type (nonprofit vs. for-profit) will moderate the effect.

**H_3_:** Organization type moderates the closing-the-gap effect such that the effect is more likely to emerge for nonprofit charities and is attenuated for profit-driven businesses.

### Overview of Studies

We test the preceding hypotheses using a combination of secondary field data and six preregistered experiments (plus seven supplemental experiments), summarized in Table 1. Examples of stimuli are included in Web Appendix A, and the complete survey questionnaires, deidentified data, analysis code, and preregistrations are available on OSF (https://osf.io/aj7g5/). In all experimental studies we aimed to recruit at least 100 participants per condition, and we followed our preregistered study plans regarding hypotheses, sample sizes, exclusion criteria, and data analysis.

**Table 1.** Summary of Results by Study Condition.

**Mean Funding Likelihood and Amount Across Study Conditions**								
Study 1 **JE vs. SE charity**	▪ Preference shift between JE and SE (H_1_). ▪ Mediation with need in JE but not SE (H_2_). ▪ DV: £100 lottery divided between charities. Preregistered. ▪ Three conditions (evaluation type: JE vs. SE far vs. SE close)							
	JE Close		JE Far		SE Close		SE Far	
Amount	34.68		65.32		51.18		55.89	

Study 2 **JE vs. SE individual**	▪ Preference shift between JE and SE (H_1_). ▪ Mediation with need in JE but not SE (H_2_). ▪ DV: likelihood and binary choice. Preregistered. ▪ Three conditions (evaluation type: JE vs. SE far vs. SE close).							
	JE Close		JE Far		SE Close		SE Far	
Likelihood	4.73		5.90		5.78		5.33	
Spending $1	26		51		63		59	

Study 3 **Three projects JE vs. SE**	▪ Preference shift between JE and SE (H_1_) with three projects. ▪ DV: incentive-compatible donation from bonus amount. Preregistered. ▪ Four conditions (evaluation type: JE vs. SE far vs. SE mid vs. SE close).							
	JE				SE			
	Close	Mid	Far	Keep	Close	Mid	Far	Keep
Amount	.08	.08	.16	.18	.27	.28	.26	.23
Percentage	16.2%	15.5%	31.8%	36.5%	53.9%	56.6%	51.7%	45.9%

Study 4 **Charity vs. business**	▪ Moderation by organization type (H_3_). ▪ Preregistered. ▪ 3 (evaluation type: JE vs. SE far vs. SE close) × 2 (organization type: charity vs. business).							
	Charity				Business			
	JE		SE		JE		SE	
	Close	Far	Close	Far	Close	Far	Close	Far
Amount	44.29	55.71	43.86	36.52	52.16	47.84	48.43	40.40

Study 5a **Gap size**	▪ Tests different combinations of gaps between the two charities. ▪ DV: £100 lottery distributed between charities and the self. Preregistered. ▪ Four conditions (gap size: 80% vs. 60% vs. 40% vs. 20%).							
	80%		60%		40%		20%	
	Close	Far	Close	Far	Close	Far	Close	Far
Amount	16.80	31.06	27.23	34.25	26.88	29.65	29.98	27.66

Study 5b **Tipping point**	▪ Robust to tipping points (H_1_). Consequential donation design. ▪ Shows mediation through need but not warmth, competence, or impact (H_2_). ▪ Preregistered. ▪ Two conditions (goal progress: high progress vs. tipping point).							
	High Progress ($2,700 out of $3,000)				Tipping Point ($2,930 out of $3,000)			
	Close to Goal		Far from Goal		Close to Goal		Far from Goal	
Amount	43.21		56.79		35.86		64.14	

Study 6 **Kiva study**	▪ Dataset with over 10,000 crowdfunding projects at varying levels of progress. ▪ Negative coefficient shows that funds decrease as progress increases (while controlling for confounding factors such as display order).							
	All Samples		Low Progress: <33%		Medium Progress: 33%–66%		High Progress: >66%	
log(1+ Progress_j,t−1_)	−.021***		−.033***		−.039***		−.189***	

Supplemental Studies								
Supplemental Study S1 **JE vs. SE charity**	▪ Preference shift between JE and SE (H_1_), replication of Study 1. ▪ Three conditions (evaluation type: JE vs. SE far vs. SE close).							
	JE Close		JE Far		SE Close		SE Far	
Amount	43.75		56.25		54.57		48.24	

Supplemental Study S2 **JE vs. SE individual**	▪ Preference shift between JE and SE (H_1_), replication of Study 2. ▪ Preregistered. ▪ Three conditions (evaluation type: JE vs. SE far vs. SE close).							
	JE Close		JE Far		SE Close		SE Far	
Likelihood	4.64		5.92		5.88		5.66	

Supplemental Study S3 **Three projects**	▪ Robust to three projects with varying middle progress project (H_1_). ▪ Preregistered. ▪ Three conditions (10%/20%/90% vs. 10%/50%/90% vs. 10%/80%/90%).							
	Far		Middle			Close		
Amount	31.38		22.14			18.19		

Supplemental Study S4 **Main effect**	▪ Tests if people donate to the charity farther from its goal in JE. ▪ Preregistered.							
	Close to Goal				Far from Goal			
Likelihood	3.40				4.60			
Amount	41.86				58.14			

Supplemental Study S5 **Completion-contingent tipping point**	▪ Tests if people donate to the charity farther from its goal when the one closer is at the tipping point and rewards are contingent on completion. ▪ Preregistered. ▪ Two conditions (completion contingent vs. not completion contingent).							
	Completion Contingent				Not Completion Contingent			
	Close to Goal		Far from Goal		Close to Goal		Far from Goal	
Amount	29.60		27.69		26.81		32.62	

Supplemental Study S6 **Relative vs. consistent progress to charity far from goal**	▪ Compares different types of goal progress in joint evaluation. ▪ Shows mediation through need. ▪ Two conditions (goal progress: relative vs. consistent) with two replicate levels each and an additional control condition.							
	Control ($300 vs. $2,700)		Relative (e.g., 10% vs. 90% Progress)			Consistent (e.g., Both at 10% Progress)		
Likelihood	4.69		4.44			4.16		
Amount	58.93		60.16			52.90		

Supplemental Study S7 **“To go” vs. “to date” framing**	▪ Preference shift between JE and SE (H_1_) with “to date” framing but not “to go” framing. ▪ DV: incentive-compatible donation from bonus amount. Preregistered. ▪ 3 (evaluation type: JE vs. SE far vs. SE close) × 2 (framing: to date vs. “to go”).							
	To Date				To Go			
	JE		SE		JE		SE	
Close	Far	Close	Far	Close	Far	Close	Far
Amount	.10	.16	.24	.28	.12	.16	.29	.25

*Notes:* DV = dependent variable, JE = joint evaluation, SE = separate evaluation.

Studies 1 and 2 test the basic effect of evaluation mode on charitable giving. We demonstrate a shift in preferences between joint and separate evaluation modes for both organizational and individual fundraisers in preregistered studies with consequential dependent measures using lotteries for participants. Next, Study 3 tests the robustness of the effect in the context of presenting three projects. This study also gives every participant a real bonus that they can choose to donate or keep for themselves, making this choice incentive-compatible for every participant. Study 4 tests the moderating role of organization type, finding that participants give more to the charity farther from its goal in joint evaluation, but not to a for-profit business farther from its goal. Across all of these studies (Studies 1–4) we measure perceptions of need, and in Studies 1, 2, and 4 we show mediation through perceptions of need for help in joint evaluation. We find that the gap in perceptions of need is larger in joint evaluation than in separate evaluation, and in Study 3 we also find that need is seen as easier to evaluate in joint evaluation.

Two additional studies (Studies 5a and 5b) test the robustness of the effect. In Study 5a we find that the effect is attenuated when the size of the gap in progress becomes smaller. In Study 5b we find that the effect is robust to participants’ donation being the tipping point for goal completion but that it is attenuated when rewards are contingent on completing the goal. Last, in Study 6 we find evidence for the focal effect in secondary data collected from a micro-crowdfunding site, Kiva. This shows generalizability of our findings to a context with a large number of diverse charities and consequential donor decisions. Across our studies, we enhance ecological validity in a number of ways: We use different charities, different fundraisers at different levels, and different projects from the same organization, as well as a large dataset with over 10,000 projects from various sectors. In addition, we use a variety of dependent variables including consequential lotteries, incentive-compatible donations from a bonus amount, and real out-of-pocket donations from users on a crowdfunding site.

## Study 1: Joint Versus Separate Evaluation—Charities

Studies 1 and 2 aimed to test our key proposition that consumers tend to donate more to a cause farther from its goal when options are evaluated jointly (vs. separately). Further, we test for the mediating role of perceived need for help. In Study 1 we examined this effect with charitable organizations, and in Study 2 we tested the effect with individual fundraisers. In Study 1, we used a lottery as a consequential dependent measure and emphasized the distance left to reach the goal to ensure participants were aware of how far from or close to reaching the goal the charity was. We also measured a potential alternative explanation for our predicted effect. Previous work has shown that perceptions of warmth can increase donations (Kervyn, Fiske, and Malone 2022). It is possible that joint evaluation makes the charity farther from its goal appear warmer than the one closer to its goal, compared with separate evaluations, and this drives greater donations.

### Method

#### Participants and design

Three hundred U.K. participants from Prolific Academic (66.3% women, 33.7% men; M_age_ = 36.28 years) took part in this study in exchange for payment. The study used a three-factor (evaluation type: joint vs. separate far vs. separate close) between-subjects design.

#### Procedure

We used two real charities (FoodShare and Second Harvest), which were pretested to ensure they were equivalent in terms of various characteristics such as liking, need, impact, and competence (Web Appendix A). Participants were assigned to one of three conditions and were presented with information about an organization closer to its goal in separate evaluation, an organization farther from its goal in separate evaluation, or both simultaneously in joint evaluation. The organization closer to the goal had raised £2,930 out of £3,000 and was labeled as having £70 to go, while the organization farther from the goal had raised £300 out of £3,000 and was labeled as having £2,700 to go. As such, the stimuli employed a combination of both “to date” framing and “to go” framing. In this and subsequent studies, the charities were counterbalanced both in terms of which organization was closer to versus farther from its goal and which organization was presented on the left versus the right in joint evaluation.

As our dependent measure, participants were then given the opportunity to make a donation allocation. They were informed that one participant would be selected in a random draw and a £100 prize would be distributed according to their choice between the two charities in the joint evaluation condition, or between the charity they saw and any charity of their choice in the separate evaluation conditions. This meant that in separate evaluation they could choose to give any amount to their personal, preferred charity and were asked to identify a charity of their choice later in the survey. After the study ended, the money was distributed as per the choices of one randomly selected participant. Participants then rated their perceptions with separate items for each organization including the organization's need (“X is in need”), impact (“Donating to X would make a bigger impact”), and warmth (“X is warm/kind/friendly/sincere”) as well as additional variables outlined in Web Appendix B on seven-point scales (1 = “Strongly Disagree,” and 7 = “Strongly Agree”). Participants then answered an attention check, an open-ended question about how they made their donation allocation decision, and demographic questions.

### Results

#### Data preparation

After we excluded 13 participants who failed the attention check (as preregistered), 287 participants were included in the final analyses (66.6% women, 33.4% men; M_age_ = 36.34 years). No participants were excluded based on their open-ended answers in any of our studies.

#### Donation amount

**Figure 1.**
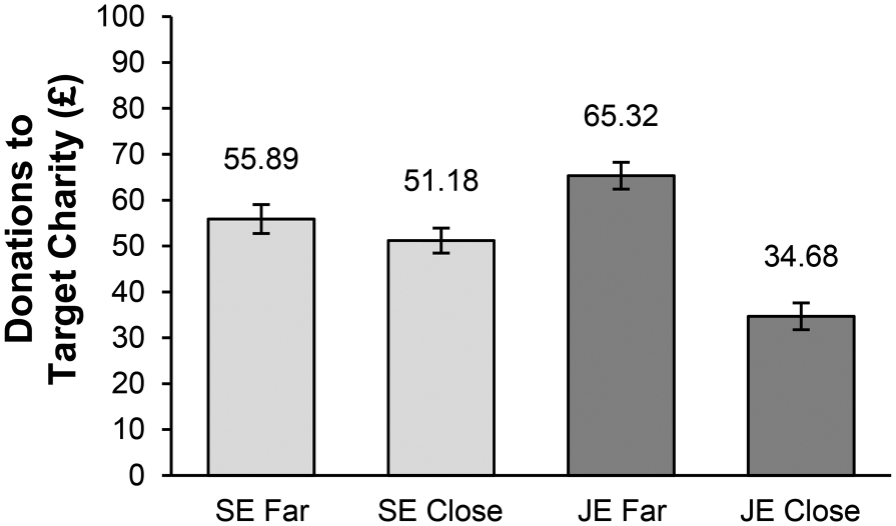
Donations to Charity Based on Distance from Goal in Joint Versus Separate Evaluation in Study 1.

*Notes:* JE = joint evaluation, SE = separate evaluation. Error bars represent ±1 standard error.

Table 1 shows average donation amounts (full distribution of donations for all studies is presented in the Web Appendix). Previous work on joint versus separate evaluation uses a hybrid t-test to compare a joint evaluation condition with two separate evaluation conditions (Hsee 1996).^1^ This test revealed a preference shift between joint evaluation and separate evaluation based on the distance of the organization from its goal (t(284) = 5.09, *p *< .001). As anticipated, in joint evaluation, a paired-samples t-test showed that respondents donated significantly more to the charity that was farther from its goal (M_far_ = 65.32 vs. M_close_ = 34.68; t(92) = 5.24, *p *< .001, d = .54). In separate evaluation, respondents did not donate more to the organization that was farther from its goal (M_close_ = 51.18 vs. M_far_ = 55.89; t(192) = 1.13, *p *= .26) (Figure 1).

#### Mediation

**Figure 2.**
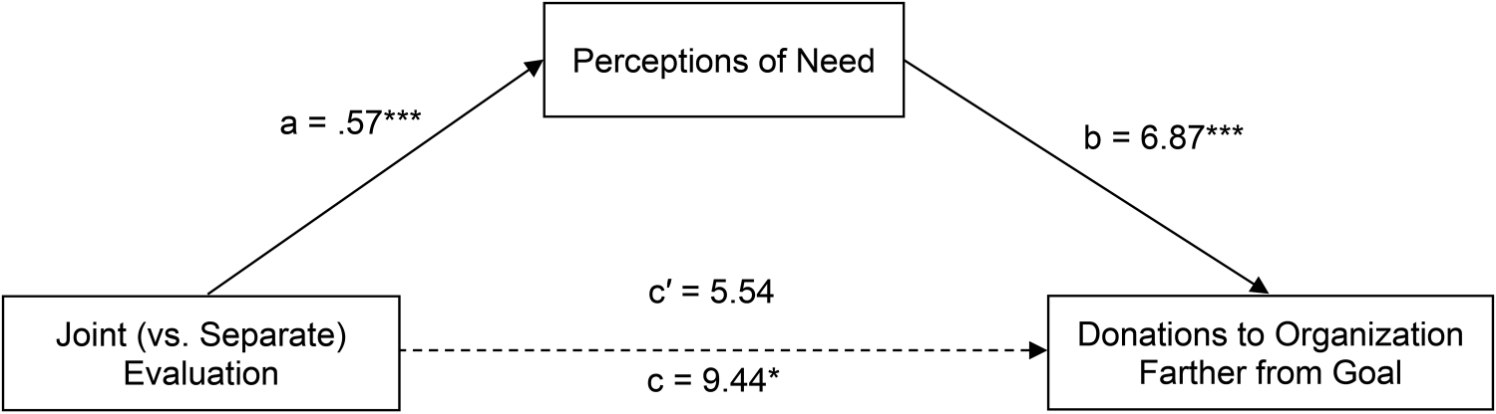
Mediation of Joint Versus Separate Evaluation on Donations Through Perceived Need in Study 1.

Given that each condition in separate evaluation had a corresponding joint evaluation condition, we carried out two mediation analyses, each with a different dependent variable. The first mediation analysis looked at donations to the charity farther from its goal, comparing the joint evaluation condition with the separate evaluation far condition. Mediation analysis using PROCESS Model 4 and 5,000 bootstrap samples showed that the effect of evaluation condition (coded 1 for joint evaluation and 0 for separate evaluation far) on donations to the charity farther from its goal was mediated by perceptions of need for help (B = 3.90, SE = 1.51, 95% CI: [1.28, 7.15]; Figure 2). A second mediation analysis looked at joint evaluation in comparison with separate evaluation close and examined donations to the charity closer to its goal using PROCESS Model 4 and 5,000 bootstrap samples. This revealed that perceived need for help did not mediate the effect of evaluation condition (coded 1 for joint evaluation and 0 for separate evaluation close) on donations to the charity closer to its goal (B = −1.79, SE = 1.41, 95% CI: [−4.79, .86]). This indicates that the comparison in joint evaluation made the need for help of the charity farther from its goal salient, which increased donations to this charity, but did not change perceptions of the charity closer to its goal. Moreover, a serial mediation model including both perceptions of need and warmth showed that warmth did not mediate donations to the charity farther from its goal (B = .77, SE = .78, 95% CI: [−.46, 2.59]) or closer to its goal (B = 1.73, SE = 1.14, 95% CI: [−.03, 4.37]).

*p < .05. **p < .01. ***p < .001.

Two research assistants, who were unaware of our hypotheses, coded the open-ended responses on whether decisions were based on need for help, fairness, impact, goal completion, or other factors. The average interrater reliability was 87%, and any inconsistencies were resolved through discussion. In joint evaluation, 52% of participants mentioned need for help, while only 17% and 23% mentioned need in the separate evaluation far and close conditions, respectively (χ^2^(2, N = 280) = 31.02, *p *< .001). This indicates that participants were more likely to mention need spontaneously as a reason for their donation decision in joint over separate evaluation. We repeated this analysis by giving the same instructions to ChatGPT 3.5 following the method outlined in Rathje et al. (2024). ChatGPT 3.5 had an accuracy of 81% when compared with the human coders and determined that 54% of participants mentioned need in the joint evaluation condition as opposed to 31% and 34% in the separate evaluation far and close conditions. We present detailed results of this analysis including results for additional codes, the codebook, sample responses, and summary tables in the Web Appendix.

### Discussion

Study 1 shows how joint evaluation mode, where two charities are presented together (vs. separately), can increase donations to the charitable organization farther from its goal. It also demonstrates that the effect is mediated by perceptions of need for help. That is, joint presentation makes the charity farther from its goal seem more in need of help, driving donations to the charity. This, combined with coded open-ended responses, supports our prediction that donation decisions are largely based on perceptions of relative need in joint (vs. separate) evaluation. We replicated Study 1 with a sample of undergraduate students (see Supplemental Study S1 in Web Appendix C).

## Study 2: Joint Versus Separate Evaluation—Individual Fundraisers

In Study 2, we explored the effect of differences in goal progress on helping behavior using individual fundraisers in a scenario taken directly from previous work (Cryder, Loewenstein, and Seltman 2013) and tested our conceptual framework by comparing joint versus separate evaluation formats. In this scenario, participants decided how much to donate to student fundraisers. We predicted that joint presentation of the fundraisers would result in more donations to the student farther from their goal, as this student would be seen as more in need of help. We also test whether sympathy toward the fundraiser farther from their goal mediates our effect; it is possible that participants feel greater sympathy for a fundraiser who has raised less money in joint over separate evaluations and that this emotional connection is what drives donations.

### Method

#### Participants and design

We recruited 299 U.S. participants from Amazon Mechanical Turk (MTurk) (30.4% women, 68.2% men, .7% other, .7% prefer not to say; M_age_ = 36.12 years) through CloudResearch. This study had a between-subjects design with three conditions (evaluation type: joint vs. separate far vs. separate close).

#### Procedures

All participants were presented with information about Olivia and/or Sienna (counterbalanced), seventh-grade students who need to sell 100 candy bars (cost: $1 each) to meet a quota for their school sports team fundraiser. The student(s) needed to sell either 2 or 32 more candy bars to meet their goal. In the joint evaluation condition both students were presented, and one was closer to the goal (needs to sell 2 more candy bars) while the other was farther from the goal (needs to sell 32 more candy bars). In the separate evaluation conditions, only one student was presented, either close to or far from the goal. The stimuli were based on previous research using this scenario (Cryder, Loewenstein, and Seltman 2013).

We then measured participants’ likelihood of buying a candy bar from each student (1 = “not likely at all,” and 7 = “very likely”). Unlike Study 1, where participants distributed a finite amount between charities, in this study they assessed their likelihood of giving to each student (independently). This meant that they could indicate very high donation likelihood for both students in the joint evaluation condition if they wanted to. Participants also indicated what they would do if they had $1 (the price of a single candy bar) with options to buy from the student(s) they had read about or to keep the money for themselves. This meant that in the joint evaluation condition they had three options (keep the money, donate to the student closer to their goal, or donate to the student farther from their goal) while in each separate evaluation condition they had two options; in the separate evaluation close condition they could either keep the money or donate to the student closer to their goal, and in the separate evaluation far condition they could either keep the money or donate to the student farther from their goal.

Participants answered a series of questions for each student fundraiser including perceived need for help (“To what extent did X need your help?”; 1 = “not at all,” and 7 = “to a great extent”) and three items on impact (e.g., “How much progress would your potential candy bar purchase make toward X's goal?”), where “X” was replaced with the name of the student(s) in the scenario. They also responded to questions related to alternative process explanations, previously tested in Cryder, Loewenstein, and Seltman (2013); three items to measure how satisfying it would be to help the student reach the goal (e.g., “How satisfying would it be to help X reach her goal?”); and three items to measure sympathy for the student (e.g., “To what extent do you feel sympathy for X?”), using a seven-point scale (1 = “not at all,” and 7 = “to a great extent”). Previous work finds that people feel greater satisfaction and impact when the fundraiser is closer to the goal (Cryder, Loewenstein, and Seltman 2013). Last, participants answered one item to measure likelihood of reaching the goal (“How likely is it that X will reach her goal?”; 1 = “not likely at all,” and 7 = “very likely”) and answered an attention check question and demographics.

### Results

#### Data preparation

After excluding participants (as preregistered) who failed the attention check, we were left with 273 participants (30% women, 68.9% men, .4% other, .7% prefer not to say; M_age_ = 35.97 years).

#### Purchase likelihood

**Figure 3.**
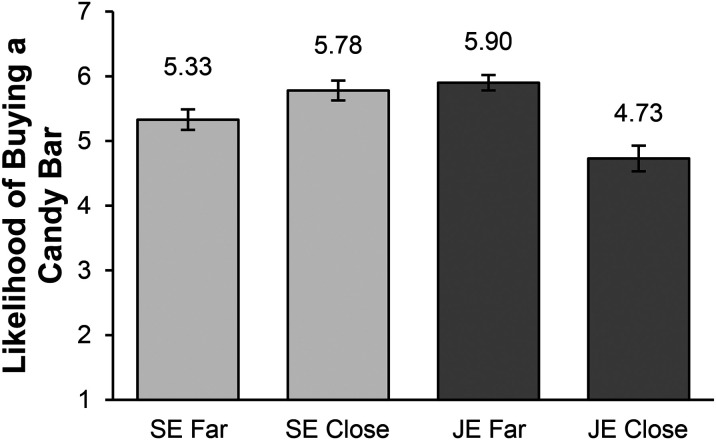
Purchase Likelihood for Candy in Joint Versus Separate Evaluation in Study 2.

*Notes*: JE = joint evaluation, SE = separate evaluation. Error bars represent ±1 standard error.

A hybrid t-test revealed a significant shift in preferences between joint evaluation and separate evaluation on likelihood of buying from the student closer to the goal (t(270) = 6.47, *p *< .001). In joint evaluation, a paired-samples t-test showed that participants were more likely to buy from the student who was farther from the goal (M = 5.90, SD = 1.14) than from the student closer to the goal (M = 4.73, SD = 1.89; t(98) = 5.13, *p *< .001, d = .54), consistent with Study 1. In separate evaluation, however, an independent-samples t-test revealed that participants were more likely to buy candy from the student who was closer to the goal (M = 5.78, SD = 1.46) than from the one who was farther from the goal (M = 5.33, SD = 1.51; t(179) = 2.02, *p *= .04), consistent with previous work on the goal gradient hypothesis. See Figure 3 for means in each condition.

#### Spending $1

A chi-square test revealed that participants chose to allocate their $1 differently across conditions (χ^2^(2, N = 273) = 16.00, *p *< .001). As anticipated, in joint evaluation, the majority of participants (56%, N = 51) chose to give to the student farther from the goal, while fewer chose to give to the student closer to the goal (29%, N = 26) or to keep the money for themselves (15%, N = 14; χ^2^(2, N = 91) = 21.97, *p *< .001). In separate evaluation, there was no difference between the choice to donate or keep the money for oneself (student closer to the goal: 35%, N = 63; farther from the goal: 32%, N = 59; keeping the money for oneself: 33%, N = 60; χ^2^(2, N = 182) = .23, *p *= .89).

#### Mediation

As this study used a mixed design where the mediators and dependent variables were measured within subjects in joint evaluation and between subjects in separate evaluation, we assessed the role of mediators for joint and separate evaluation individually. To determine the role of perceived need for help as a potential mechanism in the joint evaluation condition, we followed Montoya and Hayes’s (2017) procedure for mediation analysis with within-subject data using the MEMORE macro for SPSS with 5,000 bootstrap samples. We found a significant indirect effect where the charity farther from (vs. closer to) its goal was perceived as having a greater need for help, driving higher likelihood of purchasing candy in joint evaluation (B = .90, 95% CI: [.39, 1.42]). We then tested the roles of need for help, impact, sympathy, and satisfaction as parallel mediators in joint evaluation using the MEMORE package in SPSS. In this model, only the indirect effect of need for help was significant (B = .86, SE = .39, 95% CI: [.11, 1.66]). There was no significant indirect effect of impact (B = −.09, SE = .08, 95% CI: [−.28, .02]), sympathy (B = .001, SE = .19, 95% CI: [−.36, .38]), or satisfaction (B = .07, SE = .07, 95% CI: [−.08, .21]) on helping the student farther from the goal (see the Web Appendix for further details).

In contrast, using PROCESS Model 4, we found that in separate evaluation, need for help was not a significant mediator (B = .07, SE = .14, 95% CI: [−.19, .37]). As in previous research (Cryder, Loewenstein, and Seltman 2013), a parallel mediation model revealed that only satisfaction was a significant mediator, with greater satisfaction and donations when the organization was closer to its goal (B = .15, SE = .09, 95% CI: [.001, .36]), and neither need for help (B = .04, SE = .09, 95% CI: [−.11, .22]), impact (B = .08, SE = .08, 95% CI: [−.05, .23]), nor sympathy (B = −.03, SE = .04, 95% CI: [−.12, .04]) were significant mediators. This indicates that joint evaluation made the need for help of the student farther from the goal easier to evaluate, which increased donations to this student, but this was not the case in separate evaluation.

### Discussion

Study 2 extends the generalizability of the findings from charitable organizations in Study 1 to individuals who are fundraising for a charitable cause. We also test different dependent variables including a separate measure of donation likelihood for each charity and a decision regarding whether to buy a candy bar to support the fundraiser or to keep the money for oneself. This latter measure helps us understand how people choose to distribute money when they have the option to keep some for themselves. We also replicate the goal gradient effect in separate evaluation, but show the closing-the-gap effect in joint evaluation. Further, this study looks at the potential mediating role of several other variables examined in previous work and finds that perceptions of greater need for help for a charity leads to a need for progress in the donor that drives charitable giving. We replicate Study 2 in Supplemental Study S2 with similar results (see Web Appendix C).

## Study 3: Three Projects in Joint Versus Separate Evaluation

The previous studies looked at preference shifts in donations when two projects were presented together versus separately. In Study 3, we enhance ecological validity in several ways. First, this study tests the robustness of our effect when three projects are evaluated jointly (rather than the two-charity design used in Studies 1 and 2). Second, we use real projects from the same charity (UNICEF) that serve distinct outcomes: providing water pumps, providing vaccines, and stocking a nutrition center. This enables us to generalize our effect to a single charity promoting several projects. Third, we provide every participant with a bonus amount for taking part in the study that they can either donate to one of the projects or keep for themselves. Thus, this is a consequential, incentive-compatible decision for every participant. Fourth, this study tests our prediction that joint presentation makes it easier to evaluate need for help, as well as increases the gap in perceptions of need between charity projects. We also measure an alternative explanation: whether evaluation mode affects how justifiable need is, that is, the extent to which participants believe they should consider need in donation decisions (Li and Hsee 2019). If participants believe that the charity's need should only be considered in joint evaluation, and believe their decision should be based on other factors in separate evaluation, then justifiability rather than evaluability may drive our effects. However, we predict that participants will state that need should be considered in any donation decision and justifiability will not differ across conditions.

### Method

#### Participants and design

We recruited 601 U.K. participants (48.9% women, 49.9% men, .5% nonbinary, .2% prefer to specify, .5% prefer not to say; M_age_ = 40.64 years) from Prolific Academic. This study used a four-cell (evaluation type: joint vs. separate closest to goal vs. separate middle vs. separate farthest from goal) between-subjects design.

#### Procedure

Participants were informed at the start of the study that there would be an opportunity to donate to UNICEF^2^ and that they would be given 50 pence, in addition to their £.50 payment for participation. They could use the money to donate to the project(s) they would see or keep it for themselves. Thus, participants could choose to keep the bonus or donate it, making this a consequential decision. Participants were then shown either three charity projects (joint evaluation) or a single charity project (separate evaluation) from UNICEF. One of these projects was titled “Donate to buy a water pump” (adapted from Bonezzi, Brendl, and De Angelis [2011]). The other two projects were selected based on a pretest with six different charity projects, asking participants to compare each project to donating to buy a water pump (see Web Appendix A for details of the pretest). The projects titled “Donate to buy vaccines” and “Donate to restock a nutrition center” were rated most similar to the water pump project in impact and neediness and thus were used in this study. Thus, the three projects were for different, unrelated projects, but were all associated with the same charitable organization.

In the joint evaluation condition, the project farthest from the goal had made 10% progress, the project in the middle had made 50% progress, and the one closest to the goal had made 90% progress. In the separate evaluation conditions, participants saw one of each of these progress levels: far (10%) versus middle (50%) versus close (90%). We counterbalanced which of the three projects (water pump, vaccine, and nutrition center) were at each level of progress. Participants were then reminded that they had earned a bonus of £.50 and that they could donate their bonus to the UNICEF project(s) they just saw or keep the money for themselves, in addition to their payment. Unlike previous studies, participants were not able to split their donation in this study and thus would have to give the full bonus they had earned if they decided to donate. Participants indicated their choice, and the money was later distributed to the causes and participants according to their answers. As in the previous study, we measured participants’ perceptions of need for the projects they saw by asking “How much was each of the following projects in need?” (1 = “not at all,” and 7 = “very much”).

Participants also rated the information they saw in terms of evaluability and justifiability (adapted from Li and Hsee [2019]). For evaluability, participants saw questions reflecting the condition they were assigned to (e.g., those in the joint evaluation condition saw three charities and those in the separate evaluation condition saw one; “If an organization has raised £30 out of £300, do you have any idea how in need they are?”; 1 = “I don’t have any idea,” and 7 = “I have a clear idea”). For justifiability, participants all answered the same question (“When making donation decisions, do you think you should consider how much the organization is in need?”; 1 = “should not consider,” and 7 = “should definitely consider”). Last, participants completed an attention check.

### Results

#### Data preparation

After excluding participants who failed the attention check (as per our preregistered criteria), we were left with 586 participants (49.3% women, 49.5% men, .5% nonbinary, .2% prefer to specify, .5% prefer not to say; M_age_ = 40.80 years).

#### Donation

Average donation amounts and the percentage of people who chose to donate in each condition are summarized in Table 1. Overall donation levels (giving to at least one charitable project vs. keeping the money for oneself) were statistically higher in joint evaluation (M_donate_ = .32 and M_keep_ = .18) compared with separate evaluation (M_donate_ = .27 and M_keep_ = .23; t(584) = 2.00, *p *= .05). Moreover, the pattern of donations shifted, from giving roughly equal amounts to each charitable project in separate evaluation, to giving more to the project farthest from its goal in joint evaluation, supporting H_1_. To test this statistically, we ran two separate hybrid t-tests in joint versus separate evaluation for the project farthest from its goal compared with (1) the project closest to its goal and (2) the project in the middle, based on Hsee (1996). The hybrid t-test for the project closest to its goal revealed a significant preference shift in donations between joint and separate evaluation modes (t(441) = 2.62, *p *= .009). Following up with paired-samples t-tests in joint evaluation showed that participants also donated more to the project farthest from its goal (M = .16) than to the one closest to its goal (M = .08; t(147) = 2.79, *p *= .006, d = .31). In separate evaluation, there were no differences in donations to the project closest to (M = .27) versus farthest from (M = .26) its goal (t(293) = .38, *p *= .71).

Next, we compared the charitable project farthest from its goal to the project in the middle. The hybrid t-test for the project in the middle revealed a significant preference shift in donations between joint and separate evaluation modes (t(432) = 3.09, *p *= .002). Paired-samples t-tests in joint evaluation showed that participants donated more to the project farthest from its goal (M = .16) than to the project in the middle (M = .08; t(147) = 2.94, *p *= .004, d = .32). In separate evaluation, there were no differences in donations to the project in the middle (M = .28) versus the one farthest from its goal (t(284) = .83, *p *= .41).

#### Need

Next, we looked at participants’ perceptions of need for each of the charitable projects. As the study included a nominal choice between four options (in joint evaluation) or two options (in separate evaluation) as the dependent variable, traditional mediation analysis was not appropriate, and instead we analyzed differences in the gap in perceptions of need between the projects farthest from and closest to the goal in joint and separate evaluation. In joint evaluation, participants perceived the project farther from its goal (M = 5.92) to be more in need than the one closest to its goal (M = 4.70; M_diff_ = 1.22; t(147) = 6.14, *p *< .001). In separate evaluation, participants did not perceive a significant difference in need between the project farthest from its goal (M = 5.84) and the one closest to its goal (M = 5.59; M_diff_ = .25; t(293) = 1.50, *p *= .14).

In addition, we looked at assessments of evaluability and justifiability. Participants found it equally easy to evaluate need for the project farthest from its goal in joint (M = 4.52) and separate (M = 4.65) evaluation modes (t(288) = .58, *p *= .56). Participants also found it equally easy to evaluate need for the project in the middle in joint (M = 4.71) and separate (M = 4.53) evaluation (t(282) = .82, *p *= .41). However, participants found it easier to evaluate need for the project closest to its goal in joint evaluation (M = 4.95) than in separate evaluation (M = 4.41; t(297) = 2.39, *p *= .02). Across all conditions, need was seen as equally justifiable in joint and separate evaluation (all *p*s > .19).

### Discussion

Study 3 provides further support for our prediction that consumers give more to a charitable project farther from its goal than to one closer to its goal in joint evaluation, but not in separate evaluation. This finding remains robust to having three causes in the decision set. This study also shows that the effect holds for consequential decisions involving allocating real money to the self versus a cause. Consistent with Study 2, Study 3 shows that perceptions of need are heightened in joint (vs. separate) evaluation. Moreover, our results suggest that this may be due to need being easier to evaluate in joint evaluation.

In Supplemental Study S3 (see Web Appendix C), we test the robustness of presenting three charity projects by varying the progress of the project in the middle in a three-factor between-subjects design (middle project progress: low 20% vs. medium 50% vs. high 80% progress). We find that in all conditions participants donated more to the charity project farthest from its goal (M = 31.38) than to both the one closest to its goal (M = 18.19; t(290) = 6.26, *p *< .001) and the one in the middle (M = 22.14; t(290) = 4.23, *p *< .001). Thus, in both Study 3 and Supplemental Study S3, participants donated larger amounts to the charity project farthest from its goal when three projects were presented together, regardless of the progress level of the “middle” project.

## Study 4: Charities Versus For-Profit Businesses

Study 4 tested the moderating role of organization type. Our conceptualization proposes that people will give more to an organization farther from its goal under conditions where consumers are sensitive to the needs of others (e.g., when the context is more prosocial) and when it is made salient (i.e., in joint vs. separate evaluation). We examined this by framing products in a more versus less prosocial manner, either as part of a charitable cause positioned as a nonprofit or as part of a for-profit business. We once again tested the mediating role of perceived need.

### Method

#### Participants and design

We aimed to recruit 600 undergraduate students from a large North American university for course credit. Based on sign-up rates, we recruited 505 participants. After our preregistered exclusions (i.e., those who failed an attention check), 486 participants remained (58% women, 42% men; M_age_ = 19.28 years). This study used a 3 (evaluation mode: joint vs. separate close vs. separate far) × 2 (organization type: charity vs. business) between-subjects design.

#### Procedure

Participants were randomly assigned to see either charities or businesses that were pretested to be different in terms of organization type, but similar in terms of liking and other factors (see Web Appendix A for pretest). The organizations offered LifeLight, a solar-powered light, and Solar Bar, a foldable solar panel. In the charity condition, these products were framed as being given to those living in places without access to electricity, and in the business condition, the products were framed as for sale to travelers who are hiking, camping, and so forth. We chose to manipulate how the product was used while changing the charity versus business context so that it remained believable to participants.

In the joint evaluation condition, participants saw both organizations, with one closer to reaching its goal ($2,730 out of $3,000) and the other farther from reaching its goal ($300 out of $3,000). The order (left vs. right) and names of the organizations were counterbalanced. In the separate evaluation condition, participants saw a single organization either closer to or farther from its goal. Participants were then asked to imagine they had $100 left at the end of the month and how much of it they would give to each organization. They then rated the organizations in terms of perceived need and impact as in previous studies (as well as competence and warmth; detailed in the Web Appendix). Last, participants answered basic demographic questions.

### Results

#### Funding amount

**Figure 4.**
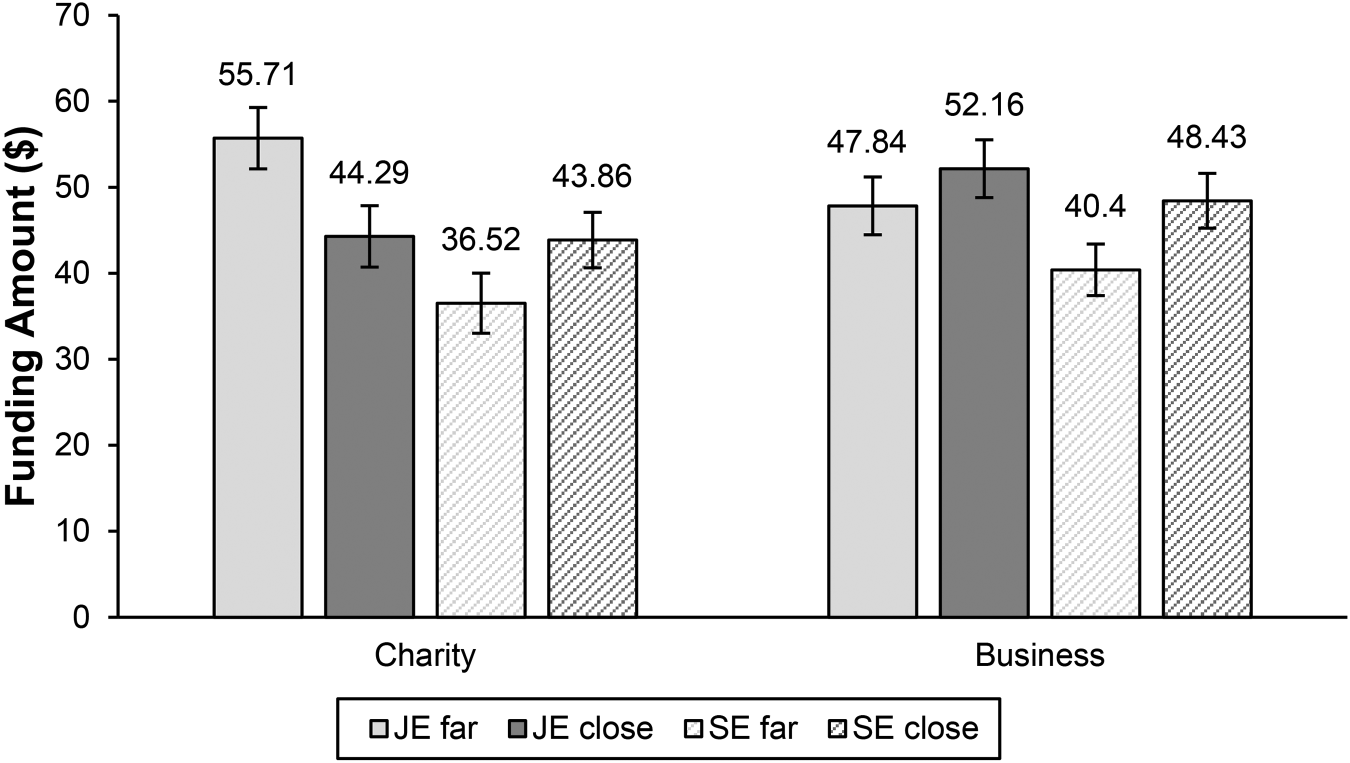
Mean Amount Given to Charity Versus Business in Joint Versus Separate Evaluation in Study 4.

*Notes:* JE = joint evaluation, SE = separate evaluation. Error bars represent ±1 standard error.

We predicted that joint evaluation would lead to higher funding intentions toward the charity farther from its goal than toward the business farther from its goal. To assess this, we ran a two-way ANOVA including evaluation type (joint vs. separate), organization type (charity vs. business), and their interaction on average amount allocated to the organization farther from its goal. This revealed a significant main effect of evaluation mode (F(1, 320) = 15.81, *p *< .001), no main effect of organization type (*p *= .55), and a marginally significant interaction between the two (F(1, 320) = 3.08, *p *= .08). Importantly, follow-up pairwise comparisons showed that funds for the charity farther from its goal were significantly higher in joint evaluation (M = 55.71) than in separate evaluation (M = 36.52; F(1, 320) = 15.74, *p *< .001), indicating that participants gave more to a charity farther from its goal in joint evaluation than in separate evaluation. Funds for the business farther from its goal were similar in joint (M = 47.84) and separate (M = 40.40) evaluation modes (F(1, 320) = 2.58, *p *= .11). See Figure 4 for means in each condition.

We then focused on the separate evaluation conditions. A two-way ANOVA predicting amount given to the target organization from organization type (business vs. charity), evaluation type (separate evaluation far vs. separate evaluation close), and their interaction showed that there was no main effect of organization type (*p *= .19) and no interaction between organization and evaluation type in separate evaluation (*p *= .92). Consistent with the goal gradient hypothesis, however, there was a main effect of evaluation type (F(1, 324) = 5.68, *p *= .02), and follow-up comparisons showed that participants gave more overall when they evaluated a single organization that was closer to its goal (M = 46.20) than when they evaluated a single organization farther from its goal (M = 38.55).

#### Mediation

To assess levels of need, we looked at the effect of separate versus joint evaluation for each type of organization individually. As expected, in joint evaluation, participants perceived the charity farther from its goal as being relatively more in need (M = 5.32) than the one closer to its goal (M = 4.47; t(75) = 2.50, *p *= .01). However, there were no differences in perceived need for the charity closer to its goal (M = 4.16) versus farther from its goal (M = 4.13) when they were evaluated separately (*p *= .89). Participants also thought the business farther from its goal was relatively more in need than the one closer to its goal in both joint evaluation (M_far_ = 5.15 vs. M_close_ = 4.23; t(81) = 3.27, *p *= .002) and separate evaluation (M_far_ = 4.25 vs. M_close_ = 3.67; t(168) = 2.26, *p *= .03). We analyzed the mediating role of perceptions of need on donation intentions using the MEMORE macro for within-subjects data with 5,000 bootstrap samples. This revealed a significant indirect effect; the charity farther from (vs. closer to) its goal was perceived as more in need, leading to greater likelihood of giving (B = 9.40, SE = 4.32, 95% CI: [1.77, 18.48]), in joint evaluation. We found no mediation by impact (see Web Appendix B).

### Discussion

This study demonstrates the moderating role of organization type, showing that the previously established effect of giving to an organization farther from its goal in joint evaluation emerges when the organization is a charitable one helping those without resources, but not when it is a for-profit business that is framed as selling products to consumers. We theorize that one condition under which people will base their resource-allocation decisions on relative levels of need is when the context is more prosocial in nature (as opposed to profit-based). The results of this study are consistent with the conceptualization that in contexts where the prosocial nature of the organizations is relatively more salient, people will give based on relative need more than they would in for-profit business contexts. In addition, this study further strengthens support for the closing-the-gap effect and finds support for the goal gradient effect in separate evaluations. Results suggest that while individuals and charities working to raise money for a cause can benefit from comparisons with another individual or charity that has made greater progress toward its goal, the same is not necessarily true for a for-profit business that positions itself on making a profit. In our context, a for-profit business trying to raise funds received more funds when it was closer to its goal, regardless of whether it was evaluated alone or with another start-up farther from its goal.

## Studies 5a and 5b: Robustness Tests and Boundary Conditions

We ran a series of additional studies to explore the robustness and boundary conditions of the closing-the-gap effect and to further test for generalizability. These studies were carried out in joint evaluation only, as we aimed to test whether participants would continue to donate more to the charity farther from the goal under varying conditions: with different combinations of goal progress (Study 5a), and when the project closer to the goal is at the tipping point (Study 5b). Greater detail on these studies is provided in Web Appendix C.

### Study 5a: Effect of Gap Size

One question is whether our effect will continue to emerge regardless of the size of the gap between the two charitable fundraisers. In this study, we test how the size of the gap between the charity closer to versus farther from the goal impacts the closing-the-gap effect. Given that our conceptualization suggests that our effects are driven by perceptions of need for help, it is possible that the effect will attenuate when the difference in goal progress between charities is lower, when need for help is less pronounced.

#### Method

This study recruited 400 participants from Prolific Academic, and after exclusions we were left with 394 participants (48.2% women, 48.7% men, 2.3% nonbinary, .8% prefer not to say; M_age_ = 38.29 years). We tested four different gap sizes in joint evaluation only (80% vs. 60% vs. 40% vs. 20% gap) in a between-subjects design. In each condition, participants read about two charity projects from UNICEF (water pump and vaccine) at different levels of goal progress: (1) 10% and 90%, (2) 20% and 80%, (3) 30% and 70%, and (4) 40% and 60%. After reading about the charities, participants were informed that one participant would be selected in a random draw after the study was completed, and a £100 prize would be distributed between the two charities and the winner according to their choice. After the study ended, the money was distributed as per the choices of one randomly selected participant.

#### Results

Overall, participants donated significantly more to the charity farther from its goal (M = £30.65, SD = 23.76) than to the one closer to its goal (M = £25.32, SD = 20.60; t(393) = 3.85, *p *< .001, d = .19). This was the case when the gap was 80% (M_far_ = £31.06, M_close_ = £16.80; t(94) = 5.46, *p *< .001) and when the gap was 60% (M_far_ = £34.25, M_close_ = £27.23; t(99) = 2.23, *p *= .03). However, there were no significant differences in donations to the two charity projects when the gap was smaller at 40% (M_far_ = £29.65, M_close_ = £26.88; t(99) = 1.15, *p *= .25) or 20% (M_far_ = £27.66, M_close_ = £29.98; t(99) = .90, *p *= .37). A regression analysis revealed a significant negative effect of gap size on extra donations to the project farther from its goal (b* *= −.22, t(392) = −4.44, *p *< .001). This indicates that, as the gap in goal progress between projects decreases, donations to the project farther from its goal become similar to donations to the one closer to its goal, indicating that gap size is a boundary condition of the closing-the-gap effect.

### Study 5b: Testing the Tipping Point

Study 5b further tests the robustness of our effect by examining it under conditions where the goal gradient effect should arguably emerge in joint evaluation contexts. Previous work demonstrates that donors are more likely to give to a project closer to its goal when they can be the one to complete the goal, sometimes referred to as being the tipping point (Anik and Norton 2020; Argo et al. 2020; Wash 2013). We examine the possibility that the closing-the-gap effect is attenuated or even reversed if the participant's donation can be the tipping point.

#### Method

We recruited 201 MTurk participants from the United States and had 160 participants after exclusions (45% women, 55% men; M_age_ = 35.41 years). The study was a one-factor, two-level (goal progress: high progress vs. tipping point) between-participants design. In both conditions, participants saw two charities, FoodShare and Second Harvest (counterbalanced). The charity farther from its goal had raised only $300 out of a goal of $3,000 (i.e., 10%). In the high-progress condition, the organization closer to its goal had raised 90%: $2,700 out of $3,000. In the tipping-point condition, the organization closer to its goal had raised 98% of its goal, or $2,930 out of $3,000, and thus needed only $70 to complete its goal. After reading about the organizations, participants were informed that there would be a consequential draw at the end of the study with the winner's allocation of the $100 being given to the two charities. Participants could thus give $70 of $100 to the charity at its tipping point to complete the goal.

#### Results

A one-sample t-test revealed that, overall, donations to the organization that was farther from its goal (M = $60.28) were greater than the midpoint (i.e., $50; M_diff_ = 10.28; t(159) = 4.42, *p *< .001, d = .32), replicating the closing-the-gap effect. An independent-samples t-test indicated that donations to the charity farther from its goal were similar in the tipping-point (M = $64.14) and high-progress (M = $56.79) conditions (t(158) = 1.59, *p *= .11, d = .25). In other words, and perhaps surprisingly, people continued to donate to the charity that was farther from its goal in joint evaluation contexts, even when the other charity was at its tipping point.

We retested this in Supplemental Study S5 (Web Appendix C) and found that preference for the charity farther from its goal over the one at its tipping point is attenuated when the donation is completion-contingent (i.e., the charity will only receive funds once it completes its goal). In this study, participants (N = 293) saw two organizations, one of which was at the tipping point. In the non-completion-contingent condition, where the charity projects received money even if they did not reach their goal, we replicated our previous effects and found that participants gave more to the charity farther from its goal (M = 32.62) than to the one closer to its goal (M = 26.81; t(146) = 2.45, *p *= .02). In the completion-contingent condition, where the charities would only receive funds if they reached their goal, there was no difference in donations to the charity farther from (M = 27.69) versus closer to (M = 29.60) its goal (t(145) = .75, *p *= .45), showing attenuation of the effect. Thus, while our effect is robust to donations to a charity that is close to its goal being at the tipping point, it is not robust when the donation is completion-contingent and the charity will only receive the funds if the goal is reached. Next, we expand the generalizability of the closing-the-gap effect in a field context where donors simultaneously consider thousands of different charities with different levels of goal progress.

## Study 6: Data from Kiva

Thus far, our studies have used relatively controlled experimental methods to provide evidence that, under conditions of joint evaluation, a charity farther from its goal tends to receive more donations than one closer to its goal. In Study 6, we use secondary data to assess the ecological validity of these findings in a context where people are donating their own money to real charitable projects. We collected data for over 10,000 projects from a nonprofit micro-crowdfunding site, Kiva (https://www.kiva.org/), to examine how progress toward a goal relates to donations using a regression framework that controls for several key project characteristics.

Kiva, a nonprofit organization that helps underserved communities, often in developing countries, raises money through micro-crowdfunding zero-interest loans. We collected data from the Kiva website using its API to pull data for 73 days (until Kiva planned to make a change to its lending policy) and collected over 32 million observations of more than 10,000 projects during this period. For the analyses presented here, we used a 10-minute-interval dataset (i.e., using observations collected every 10 minutes) and conducted robustness checks using 30- and 60-minute-interval datasets (see Web Appendix B for these additional analyses). The Kiva data consist of project-level information that remains stable over time, such as project ID and funding goal, along with project information that changes over time, including funded amount, presentation order (i.e., how projects are ordered in search results by default), and time remaining (i.e., the time left to collect funds).

We are thus able to follow a project as it raises funds and gets closer to reaching its goal. We use the change in funding amount (the total amount raised) over time for each project, as the dependent variable. We refer to this as Donation (the Donation variable indicates the amount raised during each 10-minute interval) and standardize it across projects of different sizes by calculating the change in funding for each project j from time t − 1 to time t, divided by the project's target funding amount, using the following metric:

(1) $${\mathrm{Donation}}_{j,t}=\frac{{\mathrm{Funding}}_{j,t}-\;{\mathrm{Funding}}_{j,t-1}}{{\mathrm{Target}}_{j}}.$$

Our key predictor was current fundraising goal-progress level, Progress_j,t−1_, of project j at time t − 1 (i.e., funding as a percentage of target), calculated as

(2) $${\mathrm{Progress}}_{j,t-1}=\frac{\;{\mathrm{Funding}}_{j,t-1}}{{\mathrm{Target}}_{j}}.$$

Following Cryder, Loewenstein, and Seltman’s (2013) Study 1, which also examined the effect of goal progress on donations in Kiva data, we first split the sample into three groups based on the projects’ level of progress at time t − 1, Progress_j,t−1_, as follows: (1) low progress (<33%), (2) medium progress (33%–66%), and (3) high progress (>66%), and then compared the dependent variable, Donation_j,t_, across each group. Simply comparing the mean donation in each group (as done in prior research) may be incomplete, and therefore we analyzed the effect of progress toward the goal on subsequent donations using a regression framework, which yields the following advantages: (1) the key predictor, Progress_j,t−1_, is treated as continuous rather than categorical, so that the impact of Progress_j,t−1_ on subsequent donations can be estimated in a continuous manner, revealing important patterns in the data; and (2) many potential confounds that may affect donations (such as presentation order in search results) are appropriately controlled for. In particular, presentation order may be an important confounding variable because donors are likely to focus on those projects that appear higher in the search rankings on the website and cannot consider projects they do not see. Notably, the algorithm used by Kiva rewards projects that receive funds by improving their presentation order ranking; projects that receive funds are likely to appear higher in the presentation order. This is important to understand and control for when using web data as the data context and user interface can affect consumer behavior (Boegershausen et al. 2022). Thus, a key contribution of this study over previous research (Cryder, Loewenstein, and Seltman 2013) is that it measures and controls for these confounding variables. We ran the following model (as detailed in Table 2) twice—once on the full sample and once on each group, separately:

(3) $$\log(1+{\mathrm{Donation}}_{j,t})=\beta_{1}\times \log(1+{\mathrm{Progress}}_{j,t-1})+\beta_{2}\times X_{j,t}\;+\mu_{j,\tau }+\kappa_{t}+\epsilon_{j,t}.$$

We used Donation_j,t_ as the dependent variable (with a log transformation due to its high skewness; analyses without log transformation are consistent and provided in Web Appendix B). The independent variable X_j,t_ included project characteristics that varied over time, such as lagged presentation order and remaining time until the expiration date. We also included a rich set of fixed effects to control for any unobserved project-level characteristics and/or borrower-level characteristics. Specifically, we first included project-level fixed effects that varied over time (i.e., μ_j,τ_, where τ is a higher-level time unit, described subsequently). This term controls for any unobserved project-level factors that vary over time, for example, a borrower's personal advertisement to their friends and acquaintances, or Kiva's limited-time promotion of a project (e.g., display of a project on the front page due to its urgency). Since we could not directly observe borrowers’ behavior, nor the platform's behavior, it was crucial to have this fixed-effect term to address these factors. (Note: τ is the aggregated four-hour time unit [i.e., date-hour] used in project and time-interacted fixed effects. We used τ because the time unit t of the analysis is at 10 minutes, and it cannot simply be included in a project-time interacted fixed effect. We conducted a robustness check by changing the level of the aggregated time unit τ, and the results are consistent. See Web Appendix B.) Moreover, we included a second fixed-effect term, κ_t_, which captured any unobserved factors that varied over time and were common across projects, such as holiday seasonality (e.g., greater donations during the holiday season).

**Table 2.** Summary of Results of Regression Analysis in Study 6.

	(1)	(2)	(3)	(4)
	All Samples	Low Progress: <33%	Medium Progress: 33%–66%	High Progress: >66%
log(1 + Progress_j,t−1_)	−.021*** (.001)	−.033*** (.001)	−.039*** (.002)	−.189*** (.003)
log(Presentation order_j,t_)	−.0003*** (.00001)	.00002** (.00001)	−.0005*** (.00002)	−.001*** (.00003)
log(Remaining no. of days_j,t_)	−.003*** (.0001)	−.002*** (.0001)	−.003*** (.0002)	−.005*** (.001)
Fixed effects				
Project-date-hour	Yes	Yes	Yes	Yes
Time	Yes	Yes	Yes	Yes
Borrower	Yes	Yes	Yes	Yes
Observations	36,581,629	29,312,352	6,050,667	1,218,610
R^2^	.234	.251	.375	.446

*p < .10. **p < .05. ***p < .01.

*Notes:* This table shows the parameter estimates of the regression analysis based on Equation 1. The dependent variable was log(1 + Donation_j,t_), and the independent variables were log(1 + Progress_j,t−1_), log(Presentation order_j,t_), and log(Remaining no. of days_j,t_). The regressions include various fixed-effects terms such as project and date-hour interacted, and time. The hour unit is aggregated to four hours in the project-date-hour interacted fixed-effects term. All standard errors are clustered at the project level.

Table 2 presents results of regression analyses based on Equation 1. Column 1 shows the parameter estimates from the analysis with the entire sample, and Columns 2 to 4 show the parameter estimates for the low, middle, and high groups, respectively. Notably, the coefficient of Progress_j,t−1_ is negative and significant, indicating that a 1% increase in goal progress has a negative effect on subsequent donations, reducing them by .02%. This impact is strongest for projects with higher goal progress (i.e., more than 66%), where a 1% increase in goal progress reduces subsequent donations by .19%. In contrast, projects with lower goal progress (i.e., less than 33%) show a smaller negative effect, reducing subsequent donations by only .03%. Thus, along with the results of our experimental studies, these findings provide converging evidence that charitable fundraising projects receive more funds when they are farther from rather than closer to their goal, after holding other factors constant.

## General Discussion

Across six main preregistered experiments, seven supplemental studies, and one secondary field dataset, we find converging evidence that people donate more to a charity farther from its goal when it is presented together with a charity closer to its goal. We find that this closing-the-gap effect is driven by perceptions of relative need (in Studies 1, 2, and 4 as well as replication in Supplemental Studies S1 and S2). We show that this pattern occurs when three projects from the same organization are presented together (Study 3 and Supplemental Study S3) and does *not* occur when an organization is presented on its own; in separate evaluation, individuals do not give more to a charity farther from its goal. In addition, we demonstrate moderation by organization type and show that the effect occurs in a prosocial context, but not in a for-profit context (Study 4). We find that the effect is attenuated when the gap in goal progress becomes smaller (Study 5a) and that it is robust to being able to personally complete the goal (i.e., the tipping point; Study 5b), but not when the charity will only receive funds if it reaches its goal (i.e., donations are completion contingent; Supplemental Study S5). Finally, we find evidence for this effect in a large, real-world dataset from a nonprofit micro-crowdfunding site, when controlling for presentation order and other confounding factors (Study 6). Throughout this work, we use preregistered studies, recruit large samples, and generalize across charities, individuals, and projects within a single charitable organization, using a mix of incentive-compatible, consequential donations and real-world data to increase ecological validity.

### Theoretical Contributions

This research makes several theoretical contributions. First, we build on existing research on goal pursuit in response to low versus high goal progress to reveal evaluation mode as an important moderator. While there has been much past research on goal pursuit (e.g., Cryder, Loewenstein, and Seltman 2013; Kivetz, Urminsky, and Zheng 2006; Koo and Fishbach 2008), work has not yet investigated the role of evaluation mode. We find converging evidence that consumers donate more to a charity farther from its goal when it is evaluated along with a charity closer to its goal, highlighting the importance of evaluation mode in shifting consumer decisions in charitable giving contexts. By showing that the effects of goal progress operate differently in joint versus separate evaluation in prosocial fundraising contexts, we contribute to existing work showing how consumers make inferences and decisions at different levels of goal progress (Dhar and Fishbach 2005; Fishbach and Koo 2012; Huang and Zhang 2011).

One interesting caveat is that we find statistically significant evidence of the goal gradient effect in separate evaluation in two studies (Studies 2 and 4), but no significant evidence of this in four other studies (Study 1 and Supplemental Studies S1, S2, and S7). These mixed results indicate that the strength of the goal gradient effect in separate evaluation in charitable giving contexts may be more nuanced than previously suggested. It is possible that goal gradient effects are stronger when individuals rather than charities are raising money (as in Study 2), perhaps because an identifiable target increases feelings of impact (Jenni and Loewenstein 1997). The inferences consumers make concerning goal progress in separate evaluation are likely to be context-dependent, possibly inferring greater competence at higher progress levels for for-profits, leading to higher donations (Study 4).

In addition, in Study 5a we find that the closing-the-gap effect was attenuated when the size of the gap was 40% (70%−30%), while in Study 3 we find that a similar gap of 40% between the charity farthest from its goal and the one in the middle (50%−10%) led to greater donations to the charity farthest from its goal. It is possible that the progress of the charity farthest from its goal needs to be below a certain threshold for the closing-the-gap effect to occur. Previous work supports the notion that projects tend to get “stuck in the middle” (Bonezzi, Brendl, and De Angelis 2011) and that a moderate level of goal progress can sometimes be the most demotivating. In addition, when the charities have 10% versus 50% progress, the second charity has raised five times as much, and this may make the difference feel larger than when the charities have 30% versus 70% progress, where the second charity has raised a little more than double that of the first.

Second, we illustrate the underlying psychological process for this effect. We theorize that the joint evaluation context makes relative need for help more salient and easier to evaluate and show that need for help mediates the effect (Studies 1, 2, and 4). Furthermore, we look at key theoretically relevant moderators of the closing-the-gap effect. The prosocial nature of a nonprofit (vs. for-profit) context heightens sensitivity and responsiveness to need for help (Study 4). Moreover, when the gap size between the goal progress of two charities is decreased (such that relative need is diminished) our observed effects are eliminated (Study 5a). This builds on existing work in inference making and need for progress (Fishbach, Henderson, and Koo 2011) by demonstrating that perceptions of the target’s degree of need for help can drive charitable giving.

Third, this work contributes to research on evaluation modes and the ways in which consumer behavior changes between joint and separate evaluation (Hsee 1996; Li and Hsee 2019). We build on work on the evaluability hypothesis used to explain preference shifts between joint and separate evaluation, by showing that joint presentation can make relative need easier to evaluate. Need is one of the most important criteria in donation decisions, yet consumers may not be able to accurately evaluate need in separate evaluation. Thus, when consumers consider giving to a focal charity (in separate evaluation), they may not realize the opportunity cost—that there is likely another, needier charity to which they could donate. Joint evaluation makes this salient and increases the gap in perceptions of need between charities closer to versus farther from their goal, thereby shifting donations toward charities with relatively lower levels of goal progress (and thus higher levels of need for help).

Fourth, this research contributes to the social-influence literature by illustrating a context in which consumers support a *less* popular option. Previous work shows that people tend to follow the actions of others and that communicating low norms can inadvertently discourage positive behaviors (Cialdini and Trost 1998; Habib, White, and Hoegg 2021; Schultz et al. 2007). In contrast, we show that comparative evaluation of charitable organizations can overcome the hesitancy that people often feel when asked to give to projects that lack social proof. In doing so, our research adds to the growing work in this area that addresses both theoretical and substantive challenges of promoting positive yet uncommon behaviors (Sparkman and Walton 2017).

### Directions for Future Research

There are a number of opportunities for future research to extend this work. The current work demonstrates that donations to charity were not driven by perceived impact in joint evaluation, but there may be situations in which concerns about making an impact play a more pivotal role in decision-making. Future research can investigate the effect of other information that consumers often have access to on perceptions of donation impact and how this affects decision-making in comparative evaluations. For instance, ratings of charities and projects, such as on the GiveWell or Charity Navigator websites, may play a role in decisions, particularly when two organizations have different levels of goal progress. One possibility is that such information will shift consumer preferences to the charity with more positive ratings when combined with higher goal progress. It is also possible that a cue signaling lower competence (such as less experienced employees or a lower rating on a third-party site) could be used by donors as a signal of greater need, and therefore (paradoxically) increase donations (Zhang et al. 2023). Future research can further explore several other cues that signal neediness, such as the wealth of the target group, or requests for donation of necessities rather than nonessential items. Moreover, when consumers are considering donating a large amount, this may also shift their decisions toward making an impact. The time remaining to fund the loan could also be a determining factor; donors may not wish to fund a loan that is farther from its goal if there is very little time remaining, opting instead to make an impact by funding one closer to its goal in order to ensure that it reaches the goal in time.

While we did not find evidence of the closing-the-gap effect when for-profit (vs. nonprofit) motivations were relatively more salient, it is possible that joint evaluation can drive support for for-profit organizations in certain situations. What we see as being most important for the closing-the-gap effect to emerge is whether the context activates a relative focus on need. For example, in the case of a for-profit venture, a narrative of trying to overcome adversity might be a condition under which a focus on need is activated for potential supporters. We expect that, under such conditions, we might see greater support for such a venture when it is jointly evaluated and is farther from (vs. closer to) its goal. In addition, other factors might heighten a focus on need in for-profit contexts, such as when the organization is engaged in meaningful corporate social responsibility or is owned by people from underrepresented or historically disadvantaged groups (e.g., those who are Black, Indigenous, and people of color; women; and people with disabilities). Future research can investigate the situations in which the closing-the-gap effect emerges in for-profit contexts.

Our work could also contribute to research on the underdog effect (Bradley, Lawrence, and Ferguson 2019; Kirmani et al. 2017). Underdogs are conceptualized as people, brands, or organizations facing disadvantage that have greater passion and determination to succeed (Paharia et al. 2011). Previous work on underdogs has typically used narratives and brand biographies to highlight struggles faced by smaller firms, their dedication and effort, and their competition with larger firms. In our studies, consumers are not provided with any information about the charities’ passion or determination. If consumers are making this interpretation on their own, then this indicates that underdog effects might arise when merely communicating goal progress information and could potentially be augmented under joint evaluation conditions and when the organization is more prosocial in nature. Future research could test these possibilities.

In the current research, we find the closing-the-gap effect when evaluating three projects jointly with different combinations of progress. While previous research shows that adding a third project can lead to compromise effects where preference shifts to the project in the middle (Kivetz, Netzer, and Srinivasan 2004), goal research has shown that consumers are sometimes least interested in a project when it is in the middle of goal progress (Bonezzi, Brendl, and De Angelis 2011). Thus, future research could test the circumstances under which adding a third project could lead to compromise effects.

### Practical Implications

From a managerial perspective, this work provides guidelines for charitable and nonprofit organizations, as well as crowdfunding sites. Fundraisers should carefully consider how other projects that consumers encounter simultaneously with the focal project might influence donations and create appeals that perform best when presented next to these other projects. In the case of nonprofit organizations (such as charities, governments, and social enterprises) trying to raise money for a cause that has low progress, our work suggests they can consider jointly presenting it with another, more successful cause or initiative within the organization, rather than presenting it alone. Such organizations can also adjust their strategy over time based on the project's progress toward its goal, switching to separate presentation when they are closer to reaching the goal. This is an easy, cost-effective strategy that can be implemented using the organization's existing interface or with promotional campaigns via email or social media.

Organizations can also tailor their crowdfunding pitches to their fundraising stage; in initial stages when they are farther from their goal they can consider highlighting the more prosocial aspects of their organization, and when they are closer to their goal they can focus on the profit-driven business aspects of the project. Crowdfunding sites can use insights gleaned from this research to more carefully consider how information presentation can impact outcomes. Many crowdfunding sites, including Kiva, use an algorithm that rewards projects that receive funds by moving them higher up in the search-order rankings. Our findings suggest that crowdfunding sites can adjust their algorithms to present projects farther from their goals with ones that are closer to their goals to help direct funds to the projects that need it the most.

The present research offers a robust and easy-to-implement strategy for individuals and organizations to shift donations toward a charitable cause that has made relatively little goal progress. We demonstrate that this occurs in prosocial contexts like charitable giving and when projects are presented jointly. In doing so, we provide both practitioners and academics with better insight into how consumers make decisions when donating and contribute to the literature on goal progress, evaluation mode, social norms, and charitable giving.

## Supplemental Material

sj-pdf-1-mrj-10.1177_00222437241270225 - Supplemental material for The Closing-the-Gap Effect: Joint Evaluation Leads Donors to Help Charities Farther from Their Goal Supplemental material, sj-pdf-1-mrj-10.1177_00222437241270225 for The Closing-the-Gap Effect: Joint Evaluation Leads Donors to Help Charities Farther from Their Goal by Rishad Habib, David J. Hardisty, Katherine White and Baek Jung Kim in Journal of Marketing Research

## Coeditor

Karen Page Winterich

## Associate Editor

Szu-Chi Huang

## Notes

1 Hybrid t-test formula for calculating t-value: t = ((M_JA_ − M_JB_) − (M_SA_ − M_SB_))/(S_J_^2^/N_J_ + S_SA_^2^/N_SA_ + S_SB_^2^/N_SB_)^1/2^.

2 We calculated the total donated to UNICEF in Study 3, Study 5a, Supplemental Study S5, and Supplemental Study S7 as 650 USD and donated that amount to purchase a solar-powered water pump (https://shop.unicef.ca/page/139915/shop/3?productId=3442).
