# Supplementary material for: The Closing-the-Gap Effect: Joint Evaluation Leads Donors to Help Charities Farther from Their Goal
Source: J Mark Res. 2024 Oct 30;62(1):77–96. doi: 10.1177/00222437241270225 (PMC13038148; doi:10.1177/00222437241270225)
Supplement: sj-pdf-1-mrj-10.1177_00222437241270225 - Supplemental material for The Closing-the-Gap Effect: Joint Evaluation Leads Donors to Help Charities Farther from Their Goal [file sj-pdf-1-mrj-10.1177_00222437241270225.pdf]

WEB APPENDIX

The Closing-the-Gap Effect:
Joint Evaluation Leads Donors to Help Charities Farther from Their Goal

Rishad Habib, David J. Hardisty, Katherine White and Baek Jung Kim

RISHAD HABIB
Assistant Professor, Marketing Management
Ted Rogers School of Management
Toronto Metropolitan University
Toronto, Ontario, Canada
rishad.habib@torontomu.ca

DAVID J. HARDISTY
Associate Professor, Marketing and Behavioural Science
Sauder School of Business
University of British Columbia
Vancouver, British Columbia, Canada
david.hardisty@sauder.ubc.ca

KATHERINE WHITE
Professor in Consumer Insights, Prosocial Consumption, and Sustainability
Professor, Marketing and Behavioural Science
Sauder School of Business
University of British Columbia
Vancouver, British Columbia, Canada
katherine.white@sauder.ubc.ca

BAEK JUNG KIM
Assistant Professor, Marketing
Korea University Business School
Seoul, Korea
baekjungkim@korea.ac.kr

These materials have been supplied by the authors to aid in the understanding of their paper.
The AMA is sharing these materials at the request of the authors.

WEB APPENDIX

Table of Contents

WEB APPENDIX A ... 3
Study 1 Pretest ... 3
Study 1 and Supplemental Study 1 Stimuli ... 4
Study 2 and Supplemental Study 2 Stimuli ... 5
Study 3 Pretest ... 6
Study 3 and Supplemental Study 3 Stimuli ... 7
Study 4 Pretest ... 8
Study 4 Stimuli ... 9
Study 5A Stimuli ... 10
Study 5B Stimuli ... 12
Supplemental Study S4 Stimuli ... 13
Supplemental Study S5 Stimuli ... 14
Supplemental Study S6 Stimuli ... 15
Supplemental Study S7 Stimuli ... 16
WEB APPENDIX B ... 18
Study 1 Supplementary Analyses ... 18
Study 2 Supplementary Analyses ... 21
Study 3 Supplementary Analyses ... 22
Study 4 Supplementary Analyses ... 23
Study 6 Supplementary Analyses ... 26
Distribution of Donation Data in Experimental Studies ... 33
Means for Additional Variables ... 38
Goal Progress Levels Used Across Studies ... 39
List of Links to Preregistrations ... 40
Mediation models ... 41
WEB APPENDIX C ... 43
Study 5A: Effect of Gap size ... 43
Study 5B: Testing the Tipping Point ... 45
Supplemental Study S1: Replication of JE vs. SE charities ... 49
Supplemental Study S2: Replication of JE vs. SE individuals ... 50
Supplemental Study S3: Three Projects in JE ... 52
Supplemental Study S4: Main effect ... 54
Supplemental Study S5: Tipping Point Completion Contingent in JE ... 55
Supplemental Study S6: Relative vs. Consistent goal progress and goal amount ... 56
Supplemental Study S7: To-date vs. To-go Framing ... 61
REFERENCES ... 64

WEB APPENDIX A

Study 1 Pretest

TABLE W1: RESULTS FOR FOODSHARE VS. SECOND HARVEST PRETEST

	N	Mean	SD	SE	M_{Diff}	t	df	*p*
liking	50	3.76	1.82	.26	-.24	-.93	49	.36
need	50	4.06	1.50	.21	.06	.28	49	.78
impact	50	4.34	1.52	.21	.34	1.58	49	.12
competent	50	4.14	1.36	.19	.14	.73	49	.47
capable	50	4.10	1.30	.18	.10	.54	49	.59
skillful	50	4.04	1.48	.21	.04	.19	49	.85
efficient	50	4.22	1.46	.21	.22	1.07	49	.29
warm	50	3.90	1.63	.23	-.10	-.43	49	.67
friendly	50	3.82	1.29	.18	-.18	-.99	49	.32
kind	50	4.08	1.28	.18	.08	.44	49	.66
sincere	50	4.02	1.48	.21	.02	.10	49	.92

Overall attitude towards FoodShare vs. Second Harvest:

		Mean	SD	SE	t	df	*p*
Pair 1	unfavorable:favorable	.14	1.21	.17	.82	49	.42
Pair 2	dislike:like	.22	1.35	.19	1.16	49	.25
Pair 3	bad:good	.18	1.24	.18	1.03	49	.31

Study 1 and Supplemental Study 1 Stimuli

Separate Evaluation Closer to Goal

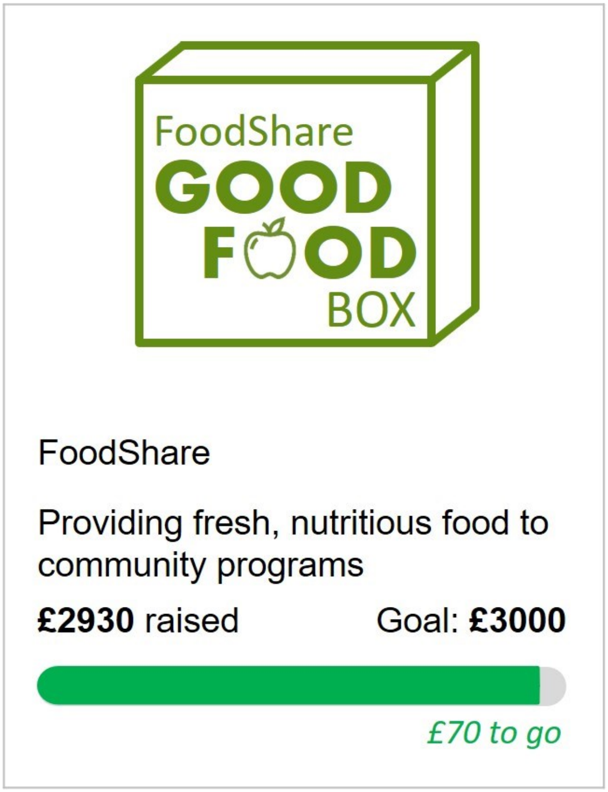

Separate Evaluation Farther from Goal

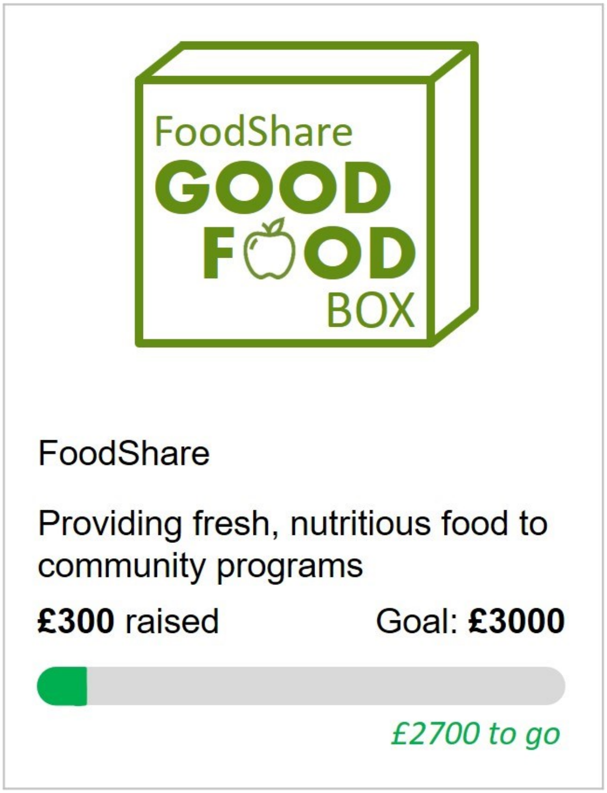

Joint Evaluation

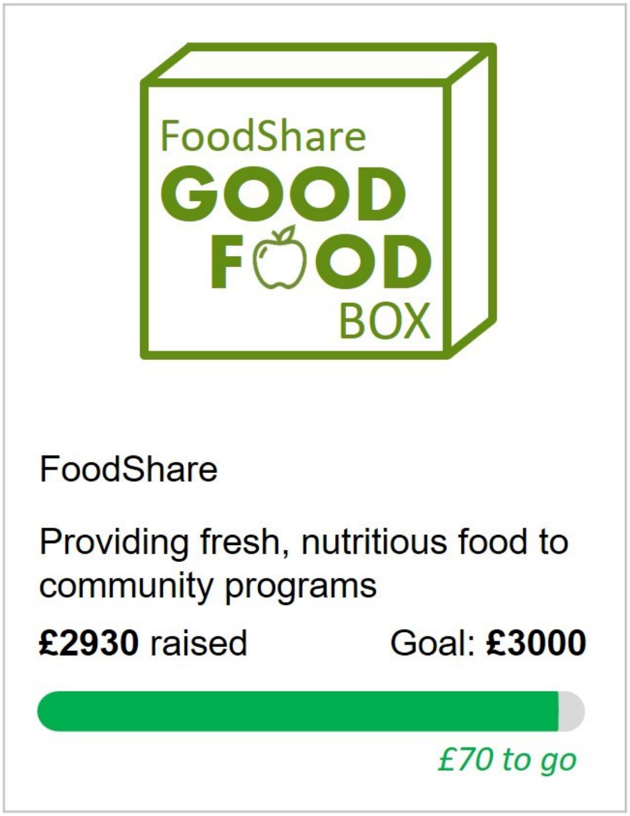

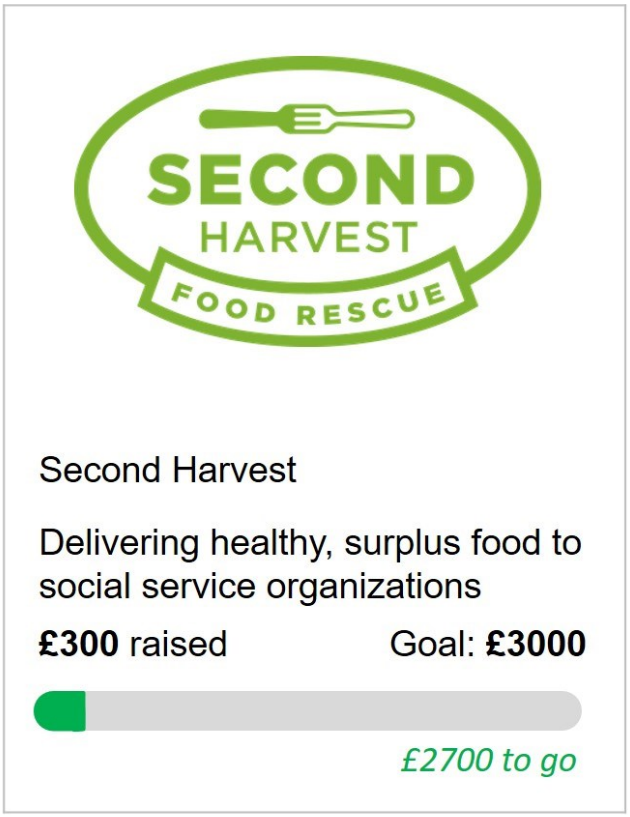

Study 2 and Supplemental Study 2 Stimuli

Separate Evaluation

Sienna is a 7th-grade student who needs to sell 100 candy bars (cost: $1 each) to meet a quota for her school sports team fundraiser. If you buy a candy bar, you will help Sienna reach her goal. She needs to sell 2 more candy bars to meet her quota, and asks you if you would be willing to buy one. (Note: Sienna is certain to reach her quota by the end of the day).

Joint Evaluation

Sienna and Olivia are two 7th-grade students who each need to sell 100 candy bars (cost: $1 each) to meet a quota for their school sports team fundraiser. If you buy a candy bar, you will help Sienna or Olivia reach her goal. Sienna needs to sell 32 more candy bars to meet her quota, and Olivia needs to sell 2 more candy bars to meet her quota. They ask you if you would be willing to buy one. (Note: They are certain to reach their quota by the end of the day).

Study 3 Pretest

We pretested six charity projects from UNICEF, comparing each of them to the “Donate to buy a Water Pump” project used by Bonezzi, Brendl, and Angelis (2011). The projects were taken from https://shop.unicef.ca/ so that we could make actual donations to the causes based on participants’ decisions. We recruited 100 participants (50% women, M = 34.46) from Prolific Academic to take part in this study.

Participants were shown two projects in pairs; one project was always the Water pump project and the other was randomly selected from our six pretest projects: Donate to buy vaccines, buy emergency shelter, buy school essentials, buy emergency medicines, restock a nutrition center, buy emergency responder kits. After seeing each pair of projects participants were asked to indicate their likelihood of donating to the projects from 1 = “water pump” to 7 = “the name of the other project”. They also indicated how they would spend $1 if they had an extra dollar by donating to either charity or keeping the money for themselves. They then rated perceptions of impact and need for each project compared to the water pump project. The means for these variables are given in the tables below. As the vaccine and nutrition center projects were rated closest to the midpoint 4 on a 7-point scale compared to the water pump project, we selected these projects for further studies.

TABLE W2: MEANS FOR UNICEF PROJECTS PRETEST FOR STUDY 3

	**N**	**Likely_donate**		**Impact**		**Need**	
		Mean	SD	Mean	SD	Mean	SD
Vaccine	100	3.59	2.050	3.76	2.046	3.70	1.997
Shelter	100	3.07	1.822	3.07	1.788	3.11	1.734
School	100	2.26	1.404	2.37	1.475	2.17	1.400
Medicine	100	3.35	1.850	3.48	1.899	3.35	1.877
Nutrition	100	3.61	1.786	3.65	1.777	3.60	1.717
Responder	100	3.25	1.806	3.30	1.861	3.25	1.806

	**Donate $1 Waterpump**	**Donate $1 Other**	**Keep for self**
Vaccine	48	40	10
Shelter	62	27	11
School	80	10	10
Medicine	54	35	11
Nutrition	53	37	10
Responder	59	31	10

Study 3 and Supplemental Study 3 Stimuli

DONATE TO BUY VACCINES

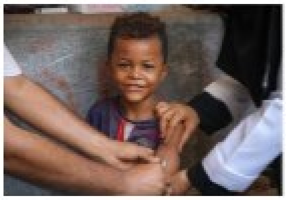

By contributing to purchase vaccines, you will help prevent serious illnesses. The life-saving combination of polio, tetanus and measles vaccines protects children against common, deadly diseases so they can grow up healthy and strong.

MONEY COLLECTED

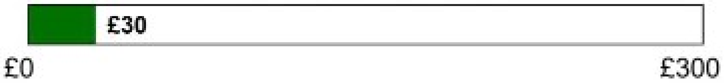

DONATE TO RESTOCK A NUTRITION CENTER

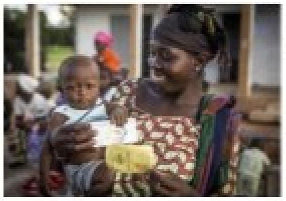

By contributing you will help reduce malnutrition. With the right nutrients early in life, a child's brain and body are able to grow to their full potential. A stock of food, therapeutic milk and oral rehydration salts helps protect malnourished children.

MONEY COLLECTED

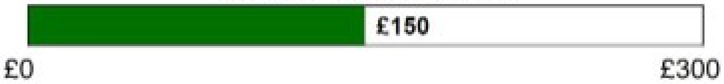

DONATE TO BUY A WATER PUMP

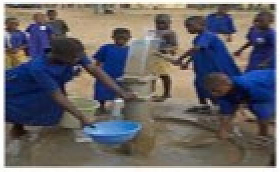

By contributing to purchase a water pump you will help provide clean, safe drinking water for an entire community. A water pump not only provides safe drinking water, it also stops women and children from having to walk miles to fetch water for their families.

MONEY COLLECTED

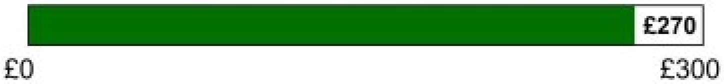

Study 4 Pretest

We pretested two products made by two different organizations: LifeLight, a solar-powered light, and Solar Bar, a foldable solar panel (stimuli below). We recruited 102 participants on Amazon Mechanical Turk in exchange for payment (50% female, M_{age} = 36.28, SD_{age} = 11.50). Participants saw two organizations, framed as either a non-profit charity or a start-up business. When the organization was framed as a charity, participants read that LifeLight is "A portable, waterproof solar light that brings light to mobile clinics, businesses, and homes without access to electricity," and that Solar Bar is "A compact, foldable solar panel that can charge an LED light, a mobile phone, and other devices in places without access to power." In the business condition, participants read that LifeLight is "A portable, waterproof solar light that is perfect for the outdoors, whether you're in the backyard or travelling off-the-grid," and that Solar Bar is "A compact, foldable solar panel that can charge an LED light, a mobile phone, and other devices while camping, hiking, and travelling."

Participants then reported how much they liked each organization ("Which charity/start-up do you like more overall?"); their perceptions of the organization's need ("Which charity/start-up do you think is more in need?"), warmth (4 items; "Which charity/start-up do you think is more warm/friendly/kind/sincere?"), and competence (4 items; "Which charity/start-up do you think is more competent/capable/skillful/efficient?"); and where they would be able to make more impact ("At which charity/start-up do you think your funds would make a bigger impact?"). All items used 7-pont scales ranging from 1 - Definitely Solar Bar to 7 – Definitely LifeStraw. Finally, participants answered an attention check question on whether they had encountered a charity or a start-up organization and completed demographic measures.

The manipulation was successful, and more than 81% of participants correctly stated the type of organization they had seen. In the charity condition, the two organizations LifeLight and Solar Bar were equivalently liked, and they were perceived as similarly in need, impactful, competent, and warm, all $ps > .13$. Likewise, in the business condition, the two organizations LifeLight and Solar Bar were equivalently liked, and they were perceived as similarly in need, impactful, competent, and warm, all $ps > .15$.

Study 4 Stimuli

A: Charity framing

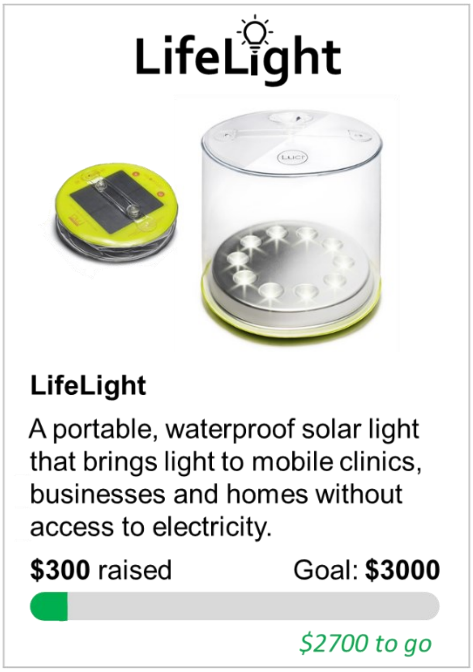

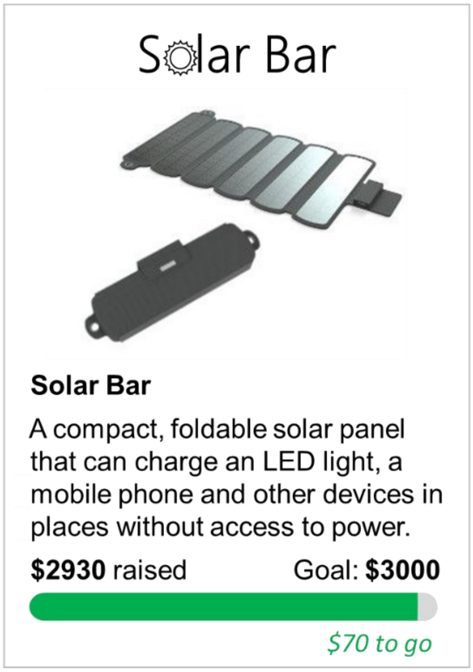

B: Business framing

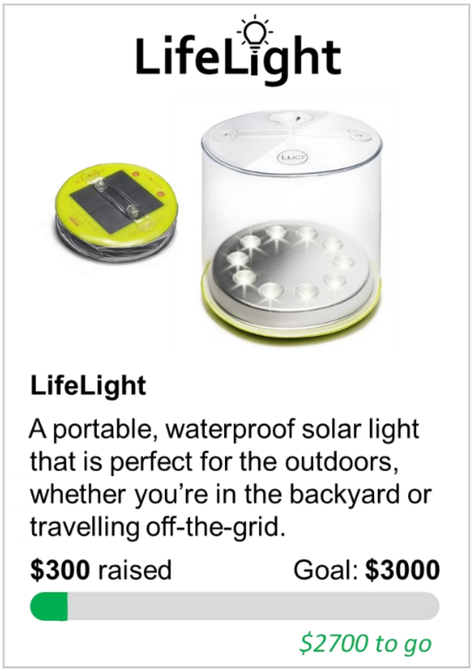

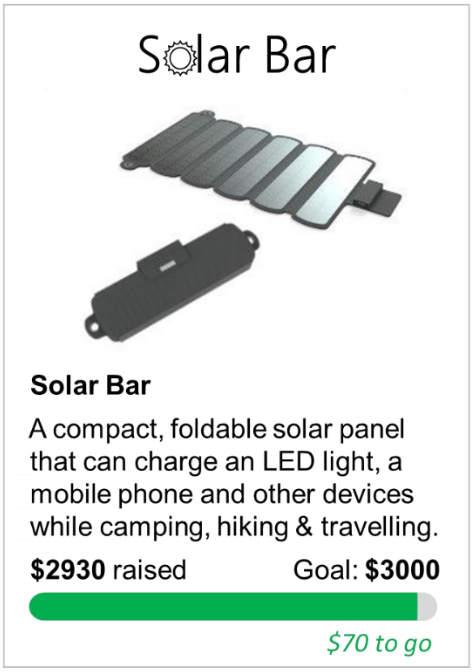

Study 5A Stimuli

Combinations in JE

Donate to UNICEF

unicef

On the next page you will see two of UNICEF's charity projects that we are raising money for. Please read the descriptions carefully.

Please continue to the following page to read the description of the projects that are collecting money.

90% & 10% progress

DONATE TO BUY VACCINES

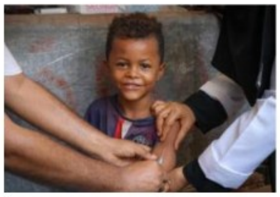

By contributing to purchase vaccines, you will help prevent serious illnesses. The life-saving combination of polio, tetanus and measles vaccines protects children against common, deadly diseases so they can grow up healthy and strong.

MONEY COLLECTED

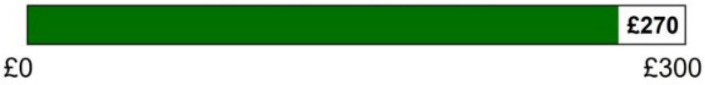

DONATE TO BUY A WATER PUMP

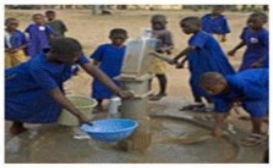

By contributing to purchase a water pump you will help provide clean, safe drinking water for an entire community. A water pump not only provides safe drinking water, it also stops women and children from having to walk miles to fetch water for their families.

MONEY COLLECTED

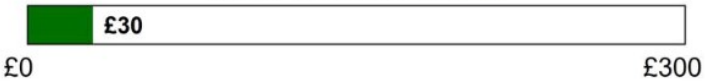

70% & 30% progress

DONATE TO BUY VACCINES

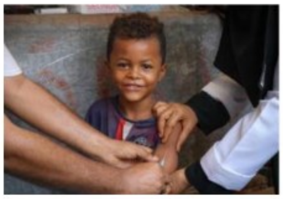

By contributing to purchase vaccines, you will help prevent serious illnesses. The life-saving combination of polio, tetanus and measles vaccines protects children against common, deadly diseases so they can grow up healthy and strong.

MONEY COLLECTED

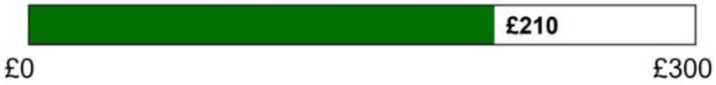

DONATE TO BUY A WATER PUMP

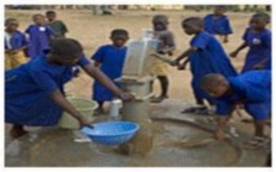

By contributing to purchase a water pump you will help provide clean, safe drinking water for an entire community. A water pump not only provides safe drinking water, it also stops women and children from having to walk miles to fetch water for their families.

MONEY COLLECTED

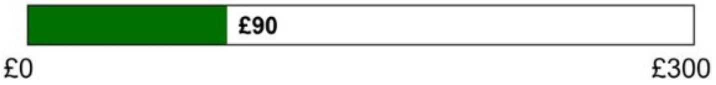

Study 5B Stimuli

High-Progress Condition

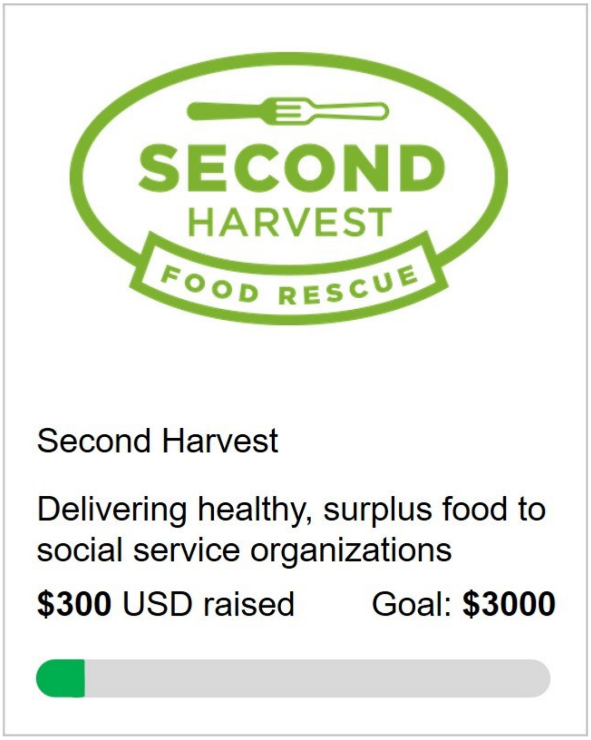

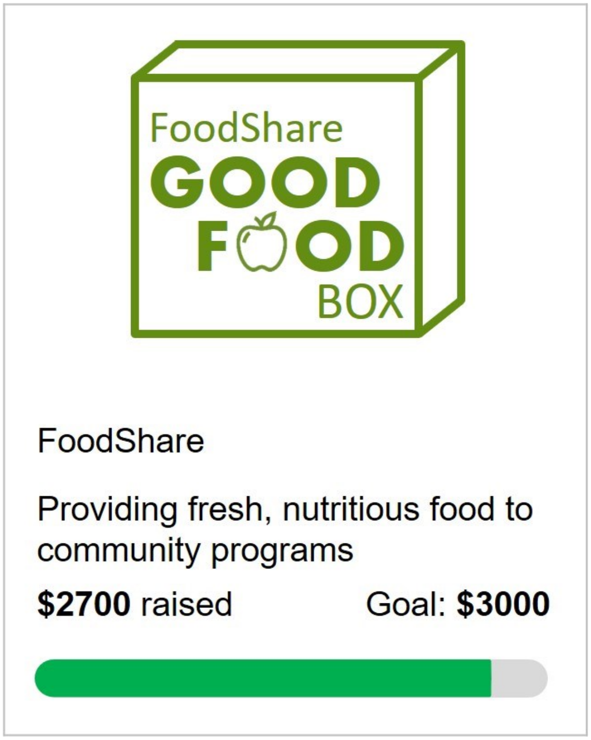

Tipping Point Condition

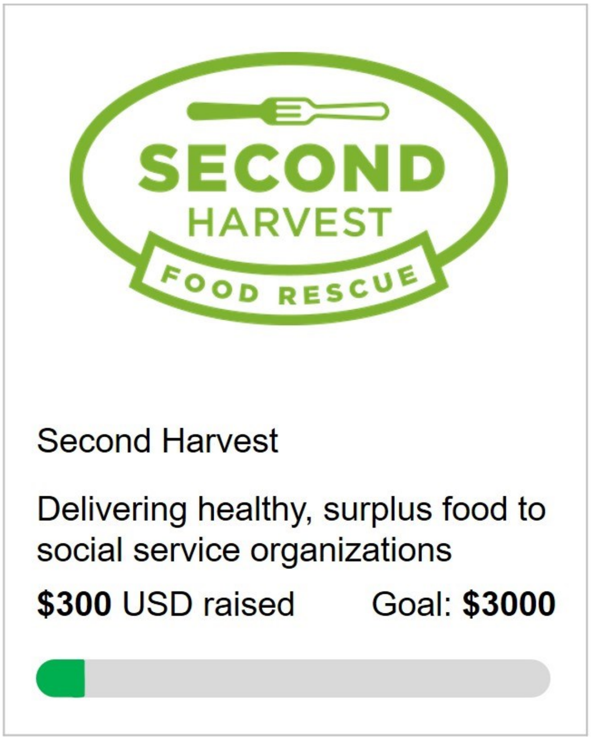

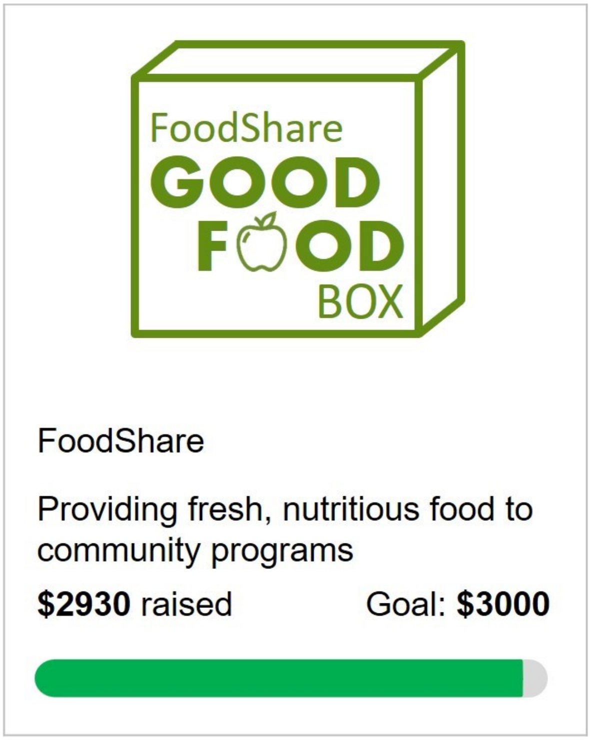

Supplemental Study S4 Stimuli

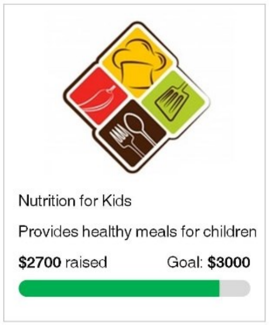

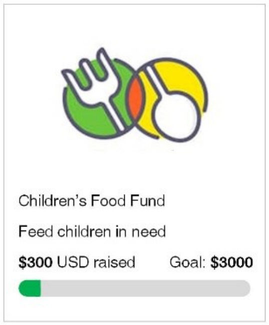

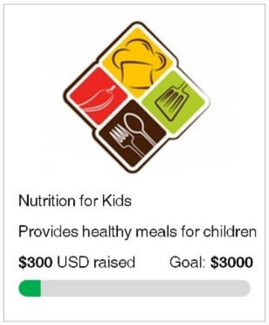

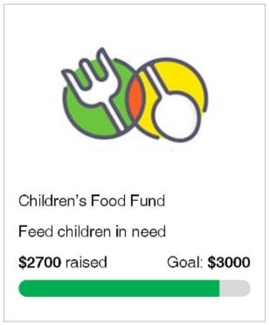

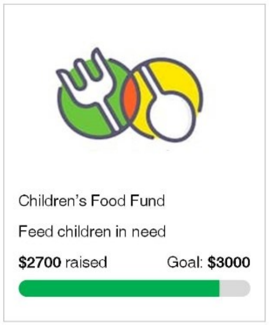

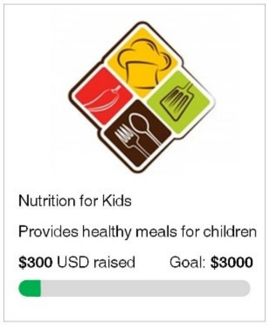

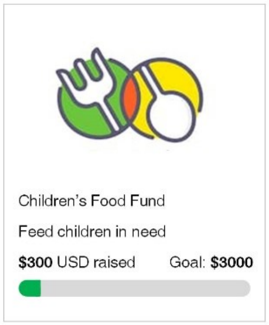

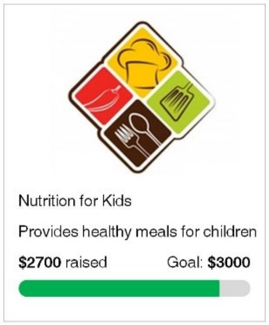

Supplemental Study S5 Stimuli

Completion contingent

Donate to UNICEF

unicef

We are collecting money for two of UNICEF's charity projects.

For your participation in today's study session, you will be entered into a prize draw to win £100. You can donate this reward to one of the charity projects or keep it for yourself.

If projects do not reach their funding goals, they will NOT receive money and you will be refunded.

Please continue to the following page to read the description of the projects we are currently collecting money for.

Not completion contingent

If projects do not reach their funding goals, they will still receive the amount they have raised.

Supplemental Study S6 Stimuli

Control

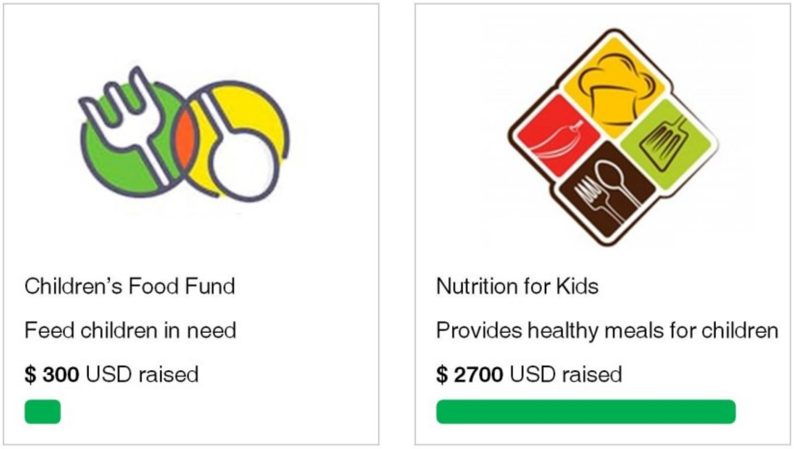

Relative Progress (medium: 10% vs. 90%)

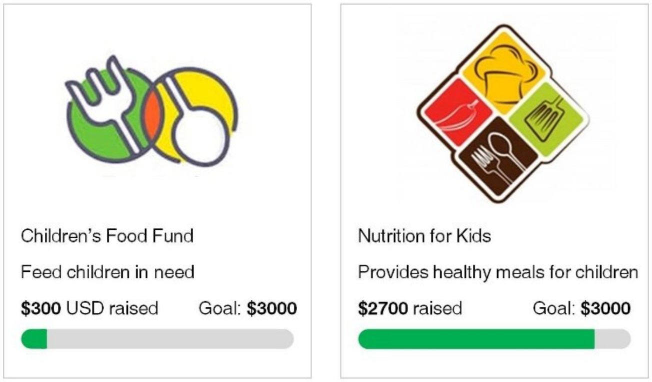

Relative Progress (low: 5% vs. 50%)

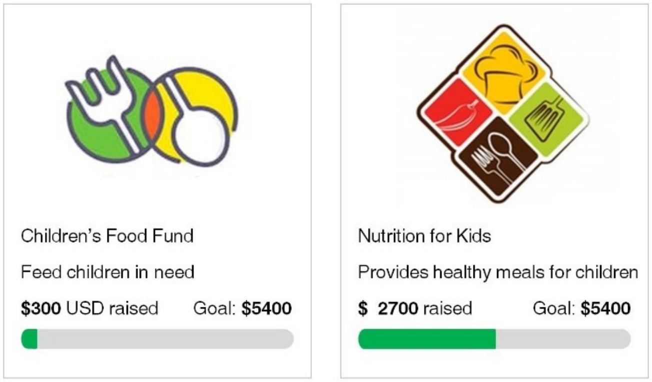

Consistent Progress (medium: 50%)

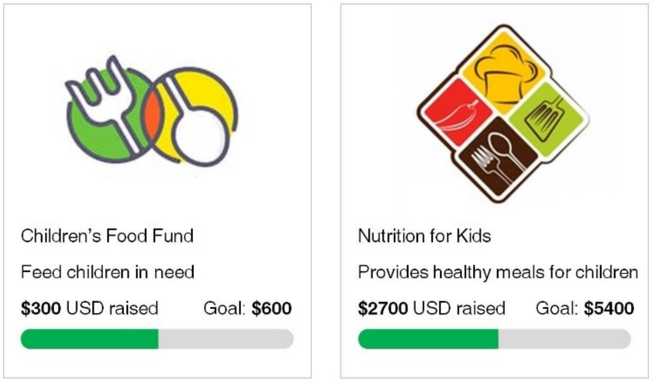

Consistent Progress (low: 10%)

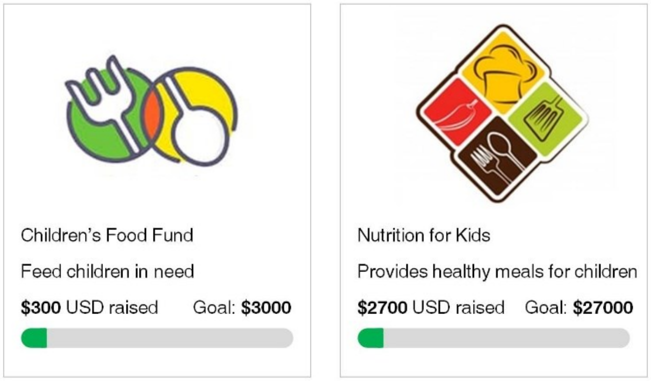

Supplemental Study S7 Stimuli

To-Date Frame

DONATE TO BUY A WATER PUMP

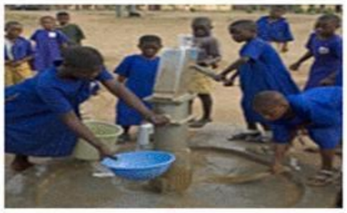

By contributing to purchase a water pump you will help provide clean, safe drinking water for an entire community. A water pump not only provides safe drinking water, it also stops women and children from having to walk miles to fetch water for their families.

AMOUNT TO DATE

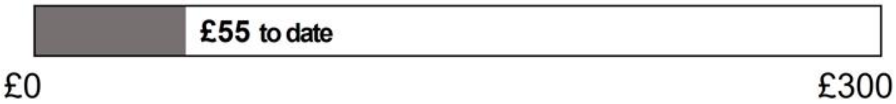

DONATE TO BUY VACCINES

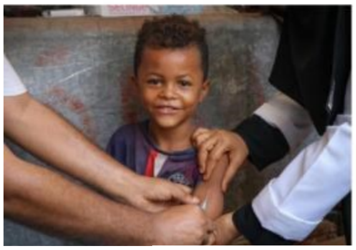

By contributing to purchase vaccines, you will help prevent serious illnesses. The life-saving combination of polio, tetanus and measles vaccines protects children against common, deadly diseases so they can grow up healthy and strong.

AMOUNT TO DATE

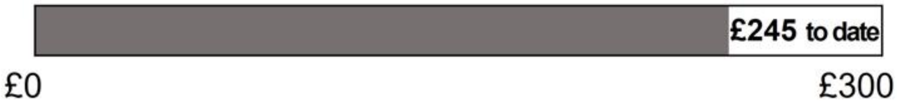

To-Go Frame

DONATE TO BUY A WATER PUMP

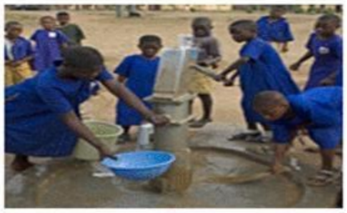

By contributing to purchase a water pump you will help provide clean, safe drinking water for an entire community. A water pump not only provides safe drinking water, it also stops women and children from having to walk miles to fetch water for their families.

AMOUNT TO GO

£245 to go

£0 £300

DONATE TO BUY VACCINES

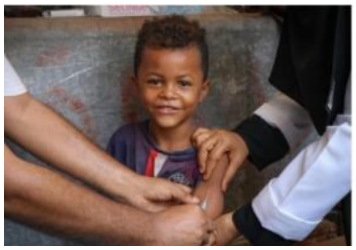

By contributing to purchase vaccines, you will help prevent serious illnesses. The life-saving combination of polio, tetanus and measles vaccines protects children against common, deadly diseases so they can grow up healthy and strong.

AMOUNT TO GO

£55 to go

£0 £300

WEB APPENDIX B

Study 1 Supplementary Analyses

Open-ended data coding.

TABLE W3: INSTRUCTIONS TO RESEARCH ASSISTANTS AND CHATGPT 3.5

	Which of these represents the reason the writer made a decision in the following text? Please mark 1 if it seems supportive of the writer's choices (i.e., the category should be mentioned as a reason *to* donate to the charity rather than a reason *not to*).	
1	Charity Need	The writer considered how one charity is more in need of funds than other charities or that the charity needed their help.
2	Fairness/Equality	The writer felt that charities should be treated fairly and equally.
3	Impact	The writer recognized the charity's potential to create a meaningful and positive change.
4	Personal need	The writer's decision to donate was significantly influenced by their own financial circumstances, as they were also in need of funds at that time.
5	Goal completion	The writer aimed to contribute to the charity's specific fundraising target, to help the charity reach its set goals.
6	Other	The decision doesn't neatly fit into the other categories, it may be due to factors such as insufficient information, personal preferences/values, or unique circumstances.

TABLE W4: SUMMARY OF OPEN-ENDED DATA CODING

	**N**	**1. Charity Need**	**2. Fairness/ Equality**	**3. Impact**	**4. Personal Need**	**5. Goal Completion**	**6. Other**
Research Assistant Coders							
JE	90	48	35	22	0	36	4
SE Far	96	16	30	40	2	10	41
SE Close	94	23	19	14	0	45	28
Chat GPT							
JE	90	49	28	17	0	47	2
SE Far	96	30	42	7	6	29	12
SE Close	94	32	28	0	0	51	19

TABLE W5: INTER-RATER RELIABILITY AND ACCURACY FOR STUDY 1

	Inter-rater reliability					
	1. Charity Need	2. Fairness/ Equality	3. Impact	4. Personal Need	5. Goal Completion	6. Other
IRR Research Assistant Coders	79%	87%	85%	98%	93%	79%
Accuracy ChatGPT	71%	84%	74%	99%	79%	78%

Sample responses:

1. Charity Need.
"The first organisation was close to their target, so although they are a good organisation they don't need the full amount. Therefore I would donate a small section to the first organisation and the remainder to the second."
"I chose to donate in full to the first option at Second Harvest had almost reached their goal, and was in less need of financial support. I felt both were worthy causes."
"I donated more to the one who needed more money to try help them reach their goal."

2. Fairness/ Equality.
“I would like to share my winning equally between my two chosen charities”
“A 50 50 share seemed fair and I could choose a charity I support if I won”
“Both charities were as deserving a each other and i thought it would be fairer to split my donation equally.”

3. Impact.
“Using food that would otherwise spoil and be sent to landfill to feed people in need scores on two fronts - 1 preventing waste, 2 feeding those in need. Whereas Foodshare doesn't prevent food wastage.”
“Given the current global situation I felt perhaps delivering food to the vulnerable was a good cause, and frankly who am I to decide to turn that away and give it to another charity.”
“I feel like the charity is doing a good thing and are helping people in need of food in the community.”

4. Personal Need.
“Whilst Foodshare is a laudable cause I prefer to give money to medical charities that have directly supported my family.”
“I thought about kind of selfishly, to be honest. I know that I am struggling too and could use the money but I still wanted to donate something that was a substantial amount of the £100.”

5. Goal Completion.
“As it was easier to help Second Harvest reach the goal I decided to put £70 to them and then the remainder helps foodshare.”
“I decided to give food share £70 so they could reach their goal”

6. Other
"I don't give to go fund mes or anything like that because of the high rate of fraud"
"Don't know anything about 'Foodshare'. I like to do a little research on companies before I would donate, including those who have a declared charitable status."
"I know nothing about this charity and would prefer the majority to go to a registered charity with a stronger background"

Competence

We ran two serial mediation analyses to test the role of competence on a) donations to the charity farther from its goal and b) donations to the charity closer to its goal. We first compared the joint evaluation condition with the separate evaluation far condition on donations to the charity farther from its goal. Mediation analysis using PROCESS model 4 and 5,000 bootstrap samples found that the effect of evaluation condition (coded JE = 1 and SE far = 0) on donations to the charity farther from its goal was mediated by perceptions of need for help, B = 3.10, SE = 1.47, 95% CI [.57, 6.27] but not perceptions of competence, B = .37, SE = .72, 95% CI [-.90, 2.04]. Next, we carried out the same analysis to compare the joint evaluation condition with the separate evaluation close condition on donations to the charity closer to its goal. This revealed that the effect of evaluation condition (coded JE = 1 and SE close = 0) on donations to the charity closer to its goal was mediated by competence, B = 2.59, SE = 1.38, 95% CI [.43, 5.74] but not by perceptions of need, B = -1.33, SE = 1.09, 95% CI [-3.61, .66]. This shows that the joint evaluation condition increased perceptions of need for the charity farther from its goal but had no effect on perceptions of need for the charity closer to its goal. In addition, this shows that the joint evaluation condition had no effect on the perceived competence of the charity farther from its goal but increased the perceived competence of the charity closer to its goal, driving donations towards the charity closer to its goal. These results indicate that competence perceptions for the charity closer to its goal may provide an indirect effect in the opposite direction.

Study 2 Supplementary Analyses

We note that we view our mediator—*need for help*—as being distinct, but related, to the construct of *need for progress* that has been examined in previous work (Fishbach, Henderson, and Koo 2011; Fishbach, Koo, and Finkelstein 2014; Koo and Fishbach 2008, 2012, 2014). Need for progress has been conceptualized as a negative affective state, on the part of the focal actor, resulting from a discrepancy between actual and expected progress that motivates goal pursuit (Fishbach, Henderson, and Koo 2011; Koo and Fishbach 2008). This construct is typically measured with a focus on the individual's levels of satisfaction with goal progress (e.g., "the progress of the campaign thus far seems satisfying." 1 - not at all, 7 - very satisfying; Fishbach, Henderson, and Koo 2011, Study 4B) and is driven by individual factors such as personal goal commitment and group identification (Fishbach, Henderson, and Koo 2011; Koo and Fishbach 2008). In contrast, we view need for help as the perception of the degree of assistance that the *target* requires to reach their goal. Thus, while need for progress is more focused on the focal actor's own degree of satisfaction and desire for goal progress, our focal construct of need for help focuses on perceptions of the target. We do, however, see these two constructs as being related and contend that an increased need for help of the target can lead to a greater need for progress in the donor that motivates future goal pursuit.

While not the focus of the current investigation, on a more granular level we expect that the perceived need for help of the charity can affect the need for progress or psychological satisfaction of the donor with the goal progress (Fishbach, Henderson, and Koo 2011). When consumers perceive a greater need for help in the target, they may focus more on making future progress, leading to a greater need for progress in turn influencing the donor's donation decision, a possibility we explore through serial mediation.

We tested whether an increased perception of need for help leads to a greater need for progress by looking at our 3-item measure of satisfaction in Study 2 (e.g., "How satisfying would it be to help X reach her goal?"). We find that need for help and satisfaction are moderately correlated for both the student closer to their goal, $r = .54$, $p < .001$ and the student farther from their goal, $r = .58$, $p < .001$. Importantly, in joint evaluation a serial mediation model reveals that a greater distance from goal leads to greater need for help and this in turn leads to higher satisfaction (i.e., need for progress) and charitable giving, 95% CI [.36, 1.41]. This supports the notion that perceptions of need for help lead to a need for progress.

Study 3 Supplementary Analyses

FIGURE W1: AVERAGE DONATIONS IN JE VS. SE IN STUDY 3

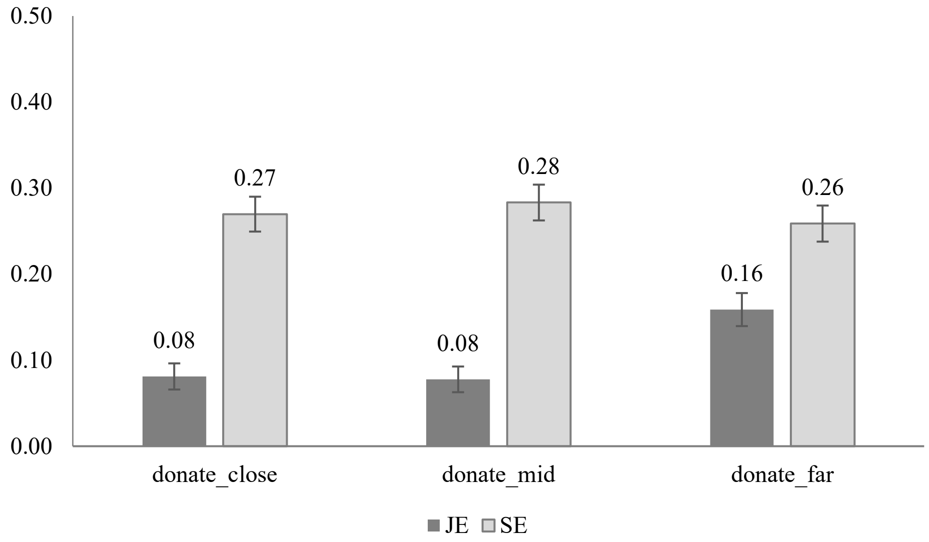

Note: Error bars represent ±1 standard error.

Study 4 Supplementary Analyses

Impact. There were no differences in perceptions of impact between charities evaluated separately, (M_{far} = 4.22 and M_{close} = 4.39), t(156) = .64, *p* = .52, or jointly, (M_{far} = 5.05 and M_{close} = 5.04), t(75) = .04, *p* = .97. There were also no differences in perceptions of impact between businesses evaluated separately, (M_{far} = 3.78 and M_{close} = 3.81), t(168) = .10, *p* = .92, or jointly, (M_{far} = 4.29 and M_{close} = 4.73), t(82) = 1.54, *p* = .13.

Next, we included perceptions of impact in a mediation model for within-subject data using the MEMORE package for SPSS to test the mediating role of impact on the relationship between distance from goal and donations in joint evaluations. This revealed no indirect effect of perceived impact on giving to the charity farther from its goal, B = .19, SE = 4.37, 95% CI [-8.4102, 8.7513]. In the case where participants saw a business organization, there was no indirect effect of perceived need, B = -4.29, SE = 2.89, 95% CI [-10.2913, 1.0453], on amount given to the business farther from its goal.

Warmth. First, we looked at differences in warmth between the two organization types, charity vs. business, in separate evaluation. This revealed that ratings of warmth for the target organization that participants encountered in separate evaluation were equal for the charity (M = 4.24) and the business (M = 4.11), t(326) = .92, *p* = .36. A two-way ANOVA looking at the organization type (charity vs. business), distance from goal (SE far vs. SE close), and their interaction in separate evaluation revealed no main effect of organization type, no main effect of distance from goal, and no interaction on perceptions of warmth (all *p*s > .35).

Next, we looked at the difference in joint evaluation between the two organization types. A two-way ANOVA including evaluation type (JE vs. SE), organization type (charity vs. business), and their interaction on perceptions of warmth of the organization farther from its goal revealed a marginally significant effect of evaluation mode, F(1, 320) = 2.91, *p* = .09, a marginal effect of organization type, F(1, 320) = 3.52, *p* = .06, but no interaction between the two, F(1, 320) = 1.71, *p* = .19. The marginal interactions indicate that participants perceived the organization to be higher in warmth when it was a charity and when they viewed it in joint evaluation. However, the lack of interaction indicates that perceptions did not differ as a function of both organization type and evaluation mode.

We also analyzed the data for charity and business conditions separately. For the charity, a paired sample t-test in joint evaluation showed no differences in warmth perceptions between the charity closer to (M = 4.73) vs. farther from (M = 4.61) its goal, t(75) = .93, *p* = .36. Similarly, an independent samples t-test also showed no differences in warmth perceptions in separate evaluation for the charity closer to (M = 4.28) vs. farther from (M = 4.21) its goal, t(156) = .35, *p* = .73. When looking at the business condition, a paired sample t-test in joint evaluation showed significantly higher warmth perceptions for the charity closer to (M = 4.44) vs. farther from (M = 4.18) its goal, t(81) = 2.15, *p* = .04. However, there were no differences when comparing the charity closer to its goal (M = 4.10) with the charity farther from its goal (M = 4.13) in separate evaluation, t(168) = .17, *p* = .87.

These analyses reveal that perceptions of warmth largely did not differ across our various conditions. Only when two businesses were evaluated jointly was there a significant difference in perceptions of warmth.

Competence. We looked at differences in competence between the two organization types, charity vs. business, in separate evaluation. This revealed that ratings of competence for the target organization that participants encountered in separate evaluation were not different for the charity (M = 4.79) or the business (M = 4.59), t(326) = 1.63, $p = .11$. A two-way ANOVA looking at the organization type, distance from goal and their interaction in separate evaluation revealed no main effect of organization type, no main effect of distance from goal and no interaction on perceptions of competence (all ps > .11).

Next, we looked at perceptions of competence in joint evaluation. A two-way ANOVA including evaluation type (JE vs. SE), organization type (charity vs. business), and their interaction on perceptions of competence of the organization farther from its goal revealed a marginal effect of evaluation mode, F(1, 320) = 2.69, $p = .10$, no effect of organization type, F(1, 320) = .90, $p = .34$, and no interaction between the two, F(1, 320) = .02, $p = .90$. Thus, perceptions of competence were not different for the charity vs. the business when the organization was closer to vs. farther from its goal.

We also analyzed the data for charity and business conditions separately. For the charity, a paired sample t-test in joint evaluation showed higher competence perceptions for the charity closer to its goal (M = 5.25) vs. farther from its goal (M = 4.88), t(75) = 2.99, $p = .004$. An independent samples t-test also showed no differences in competence perceptions in separate evaluation for the charity closer to (M = 4.91) vs. farther from (M = 4.67) its goal, t(156) = 1.44, $p = .15$. For the business a paired sample t-test in joint evaluation showed higher competence perceptions for the charity closer to (M = 5.17) vs. farther from (M = 4.75) its goal, t(81) = 2.75, $p = .007$. An independent samples t-test also showed no differences in competence perceptions in separate evaluation for the business closer to (M = 4.60) vs. farther from (M = 4.57) its goal, t(168) = .18, $p = .86$. This indicates that perceived competence was higher in joint evaluation both for the charity and for the business closer to its goal, while this difference did not exist when the organizations were evaluated separately.

TABLE W6: SUMMARY OF MEANS FOR STUDY 4

**Study 4** *Charity vs Business*								
	Charity				Business			
	JE		SE		JE		SE	
	Close	Far	Close	Far	Close	Far	Close	Far
Need	4.47	5.32	4.16	4.13	4.23	5.15	3.67	4.25
Impact	5.04	5.05	4.39	4.22	4.73	4.29	3.81	3.78
Competence	5.25	4.88	4.91	4.67	5.17	4.75	4.60	4.57
Warmth	4.73	4.61	4.28	4.21	4.44	4.18	4.10	4.13

Mediation. We looked at the effect of distance from goal overall, and for each type of organization. First, we focused on joint evaluation and analyzed the mediating role of perceptions of impact, warmth and competence separately as mediators. This revealed that perceptions of warmth (B = -3.33, 95%CI [-7.16, -.28]) was a significant mediator but neither competence (B = -5.58, 95% CI [-9.54, -2.14] nor impact (B = -2.69, 95%CI [-7.67, 2.27]) were significant mediators.

Second, we focused on the charity condition. Separate mediation models revealed that impact (B = -.19, 95%CI [-8.11, 8.93]) and warmth (B = -2.06, 95%CI [-7.44, 2.19]) were not significant mediators. However, competence (B = -5.94, 95%CI [-11.92, -.72]) was a significant mediator between distance from goal and donation intentions in joint evaluation when it was included in the model on its own. Third we focused on the business condition. In this case, impact (B = -4.29, 95%CI [-10.57, 1.23]) was not a significant mediator, but both warmth (B = -4.26, 95%CI [-10.26, -.15]) and competence (B = -5.35, 95%CI [-10.66, -.65]) were significant mediators.

This indicates that consumers may be basing their decisions on competence when it comes to giving to charities and businesses. For both charities and businesses, competence was higher when the organization was closer to reaching its goal and this led to lower funding for the organization.

.

Study 6 Supplementary Analyses

1. Robustness checks with different time intervals

TABLE W7: ROBUSTNESS CHECKS WITH 10-MINUTE INTERVAL DATASET

	Dependent variable:							
	**log(1+share-amount$_{j,t}$(percentage))**				**log(1+share-amount$_{j,t}$(dollars))**			
	All samples	Low	Middle	High	All samples	Low	Middle	High
**log(1+Progress$_{j,t-1}$)**	-0.021***	-0.033***	-0.039***	-0.189***	-0.741***	-2.101***	-3.284***	-9.174***
	(0.001)	(0.001)	(0.002)	(0.003)	(0.023)	(0.030)	(0.061)	(0.140)
log(Popularity$_{j,t}$)	-0.0003***	0.00002**	-0.0005***	-0.001***	-0.010***	0.004***	-0.025***	-0.063***
	(0.00001)	(0.00001)	(0.00002)	(0.0000)	(0.001)	(0.001)	(0.001)	(0.002)
log(Remaining # days$_{j,t}$)	-0.003***	-0.002***	-0.003***	-0.005***	-0.170***	-0.113***	-0.156***	-0.388***
	(0.0001)	(0.0001)	(0.000)	(0.001)	(0.008)	(0.006)	(0.016)	(0.077)
**Fixed Effects**								
Project * Date * Hour FE (4 hours)	Yes	Yes	Yes	Yes	Yes	Yes	Yes	Yes
Time (30 mins) FE	Yes	Yes	Yes	Yes	Yes	Yes	Yes	Yes
Borrower FE	Yes	Yes	Yes	Yes	Yes	Yes	Yes	Yes
Observations	36,581,629	29,312,352	6,050,667	1,218,610	36,581,629	29,312,352	6,050,667	1,218,610
R^2	0.234	0.251	0.375	0.446	0.239	0.188	0.307	0.397

Note: $^{*}p < 0.1$; $^{**}p < 0.05$; $^{***}p < 0.01$; *all standard errors are clustered at the loan level.*

TABLE W8: ROBUSTNESS CHECKS WITH 30-MINUTE INTERVAL DATASET

	Dependent variable:							
	**log(1+share-amount$_{j,t}$(percentage))**				**log(1+share-amount$_{j,t}$(dollars))**			
	All samples	Low	Middle	High	All samples	Low	Middle	High
**log(1+Progress$_{j,t-1}$)**	-0.138***	-0.124***	-0.172***	-0.359***	-5.924***	-9.006***	-12.822***	-18.609***
	(0.003)	(0.004)	(0.005)	(0.007)	(0.073)	(0.107)	(0.172)	(0.316)
log(Popularity$_{j,t}$)	-0.001***	0.00002	-0.001***	-0.001***	0.003***	0.017***	-0.033***	-0.041***
	(0.00003)	(0.00003)	(0.00005)	(0.0001)	(0.001)	(0.001)	(0.002)	(0.004)
log(Remaining # days$_{j,t}$)	-0.006***	-0.005***	-0.007***	-0.021***	-0.334***	-0.285***	-0.419***	-1.147***
	(0.0004)	(0.0005)	(0.001)	(0.003)	(0.023)	(0.028)	(0.041)	(0.158)
**Fixed Effects**								
Project * Date * Hour FE (4 hours)	Yes	Yes	Yes	Yes	Yes	Yes	Yes	Yes
Time (30 mins) FE	Yes	Yes	Yes	Yes	Yes	Yes	Yes	Yes
Borrower FE	Yes	Yes	Yes	Yes	Yes	Yes	Yes	Yes
Observations	9,715,027	1,998,818	391,719	12,105,564	9,715,027	1,998,818	391,719	12,105,564
R^2	0.461	0.589	0.605	0.444	0.366	0.479	0.507	0.403

Note: $^{*}p < 0.1$; $^{**}p < 0.05$; $^{***}p < 0.01$; *all standard errors are clustered at the loan level.*

TABLE W9: ROBUSTNESS CHECKS WITH 60-MINUTE INTERVAL DATASET

	Dependent variable:							
	**log(1+share-amount$_{j,t}$(percentage))**				**log(1+share-amount$_{j,t}$(dollars))**			
	All samples	Low	Middle	High	All samples	Low	Middle	High
**log(1+Progress$_{j,t-1}$)**	-0.193***	-0.186***	-0.307***	-0.676***	-6.669***	-10.740***	-17.086***	-34.595***
	(0.003)	(0.004)	(0.006)	(0.012)	(0.064)	(0.094)	(0.187)	(0.534)
log(Popularity$_{j,t}$)	0.001***	0.001***	0.00002	-0.001***	0.099***	0.099***	0.047***	0.014**
	(0.00004)	(0.00005)	(0.00010)	(0.0001)	(0.002)	(0.002)	(0.003)	(0.006)
log(Remaining # days$_{j,t}$)	-0.016***	-0.009***	-0.014***	-0.010**	-0.744***	-0.482***	-0.772***	-0.645**
	(0.0010)	(0.0010)	(0.001)	(0.004)	(0.030)	(0.027)	(0.070)	(0.261)
**Fixed Effects**								
Project * Date * Hour FE (4 hours)	Yes	Yes	Yes	Yes	Yes	Yes	Yes	Yes
Time (30 mins) FE	Yes	Yes	Yes	Yes	Yes	Yes	Yes	Yes
Borrower FE	Yes	Yes	Yes	Yes	Yes	Yes	Yes	Yes
Observations	6,752,555	5,475,449	1,075,115	201,991	6,752,555	5,475,449	1,075,115	201,991
R^2	0.592	0.62	0.707	0.727	0.532	0.522	0.601	0.645

Note: $^{*}p < 0.1$; $^{**}p < 0.05$; $^{***}p < 0.01$; *all standard errors are clustered at the loan level.*

2. Robustness checks with different aggregated time units in the fixed-effects term

TABLE W10: ROBUSTNESS CHECKS WITH 10-MINUTE INTERVAL DATASET (WITH 3-HOUR AGGREGATION)

	Dependent variable:							
	**log(1+share-amountj,t(percentage))**				**log(1+share-amountj,t(dollars))**			
	All samples	Low	Middle	High	All samples	Low	Middle	High
**log(1+Progressj,t-1)**	-0.036***	-0.048***	-0.058***	-0.230***	-1.145***	-2.690***	-4.225***	-11.090***
	(0.001)	(0.001)	(0.002)	(0.003)	(0.024)	(0.032)	(0.067)	(0.155)
log(Popularityj,t)	-0.00003**	0.0002***	-0.0003***	-0.001***	0.011***	0.020***	-0.006***	-0.044***
	(0.00001)	(0.00001)	(0.00002)	(0.00003)	(0.001)	(0.001)	(0.001)	(0.002)
log(Remaining # daysj,t)	-0.004***	-0.002***	-0.003***	-0.005***	-0.186***	-0.124***	-0.179***	-0.319***
	(0.0001)	(0.0001)	(0.0003)	(0.001)	(0.007)	(0.007)	(0.017)	(0.081)
**Fixed Effects**								
Project * Date * Hour FE (3 hours)	Yes	Yes	Yes	Yes	Yes	Yes	Yes	Yes
Time (30 mins) FE	Yes	Yes	Yes	Yes	Yes	Yes	Yes	Yes
Borrower FE	Yes	Yes	Yes	Yes	Yes	Yes	Yes	Yes
Observations	36,581,629	29,312,352	6,050,667	1,218,610	36,581,629	29,312,352	6,050,667	1,218,610
R2	0.261	0.280	0.399	0.465	0.263	0.212	0.307	0.415

Note: $^{*}p < 0.1$; $^{**}p < 0.05$; $^{***}p < 0.01$; *all standard errors are clustered at the loan level.*

TABLE W11: ROBUSTNESS CHECKS WITH 10-MINUTE INTERVAL DATASET (WITH 5-HOUR AGGREGATION)

	Dependent variable:							
	**log(1+share-amountj,t(percentage))**				**log(1+share-amountj,t(dollars))**			
	All samples	Low	Middle	High	All samples	Low	Middle	High
**log(1+Progressj,t-1)**	-0.014***	-0.024***	-0.027***	-0.160***	-0.483***	-1.602***	-2.482***	-7.658***
	(0.001)	(0.001)	(0.001)	(0.003)	(0.019)	(0.024)	(0.051)	(0.134)
log(Popularityj,t)	-0.0004***	-0.00002**	-0.0005***	-0.001***	-0.021***	-0.002***	-0.031***	-0.069***
	(0.00001)	(0.00001)	(0.00002)	(0.00003)	(0.001)	(0.0005)	(0.001)	(0.002)
log(Remaining # daysj,t)	-0.002***	-0.002***	-0.002***	-0.004***	-0.141***	-0.097***	-0.123***	-0.330***
	(0.0001)	(0.0001)	(0.0002)	(0.001)	(0.006)	(0.006)	(0.013)	(0.066)
**Fixed Effects**								
Project * Date * Hour FE (5 hours)	Yes	Yes	Yes	Yes	Yes	Yes	Yes	Yes
Time (30 mins) FE	Yes	Yes	Yes	Yes	Yes	Yes	Yes	Yes
Borrower FE	Yes	Yes	Yes	Yes	Yes	Yes	Yes	Yes
Observations	36,581,629	29,312,352	6,050,667	1,218,610	36,581,629	29,312,352	6,050,667	1,218,610
R2	0.221	0.239	0.365	0.435	0.227	0.176	0.296	0.386

Note: $^{*}p < 0.1$; $^{**}p < 0.05$; $^{***}p < 0.01$; *all standard errors are clustered at the loan level.*

3. Robustness checks without log transformation.

TABLE W12: ROBUSTNESS CHECKS WITHOUT LOG TRANSFORMATION

	**(1)**<br>**All Samples**	**(2)**<br>**Low Progress**<br>**< 33%**	**(3)**<br>**Medium Progress**<br>**33 - 66%**	**(4)**<br>**High Progress**<br>**> 66%**
log(1+ $Progress_{j,t-1}$)	-0.015***	-0.026***	-0.023***	-0.108***
	(0.001)	(0.001)	(0.001)	(0.002)
log(Presentation $order_{j,t}$)	0.000***	0.000***	-0.000***	-0.000***
	(0.000)	(0.000)	(0.000)	(0.000)
log(Remaining # $days_{j,t}$)	-0.713	-0.078	-9.804***	0.341
	(0.560)	(0.419)	(3.033)	(2.461)
Fixed effects				
Project-Date-Hour	Yes	Yes	Yes	Yes
Time	Yes	Yes	Yes	Yes
Borrower	Yes	Yes	Yes	Yes
Observations	36,581,629	29,312,352	6,050,667	1,218,610
R^2	0.234	0.261	0.381	0.448

This table presents results of regression analyses based on equation (1) without log transformation. Column (1) shows the parameter estimates from the analysis with the entire sample, and columns (2) to (4) show the parameter estimates for the low, middle, and high groups, respectively. The main result is consistent with the one from log transformation: the coefficient of $Progress_{j,t-1}$ is negative and significant, indicating that a 1% increase in goal progress has a negative effect on subsequent donations. This impact is strongest for projects with higher goal progress (i.e., more than 66%), where a 1% increase in goal progress reduces subsequent donations by 0.11%. Although projects with lower goal progress show a greater negative effect than middle progress, the difference could be driven by the skewed distribution of the DV and the IV. Thus, we believe the analysis with the log transformation is more robust and accurate.

4. Distribution of Residuals.

FIGURE W2: NON-NORMALITY OF THE RESIDUALS IN STUDY 6

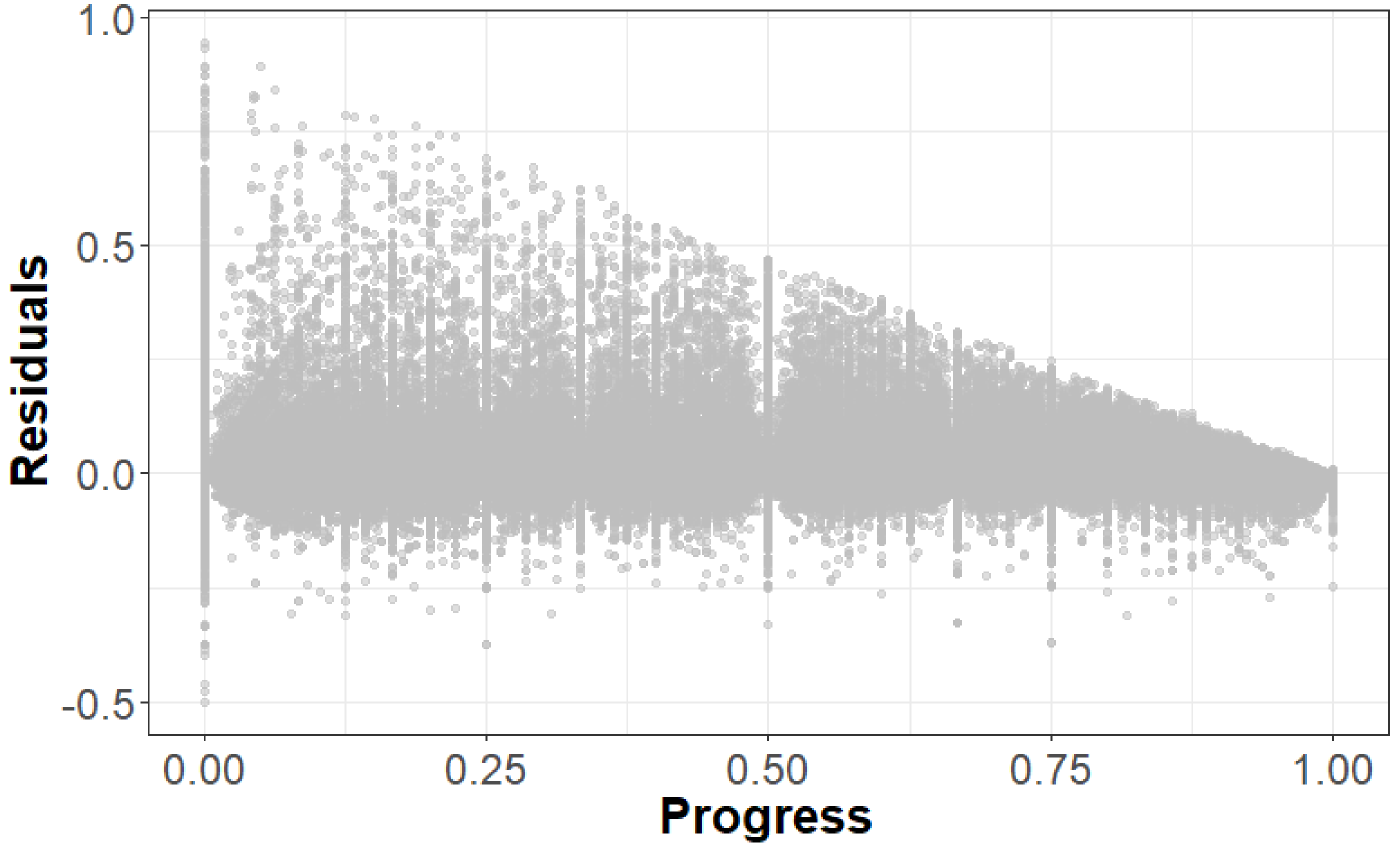

Distribution of Donation Data in Experimental Studies

Study 1

FIGURE W3: DISTRIBUTION OF DONATIONS TO THE CHARITY CLOSER TO ITS GOAL IN STUDY 1

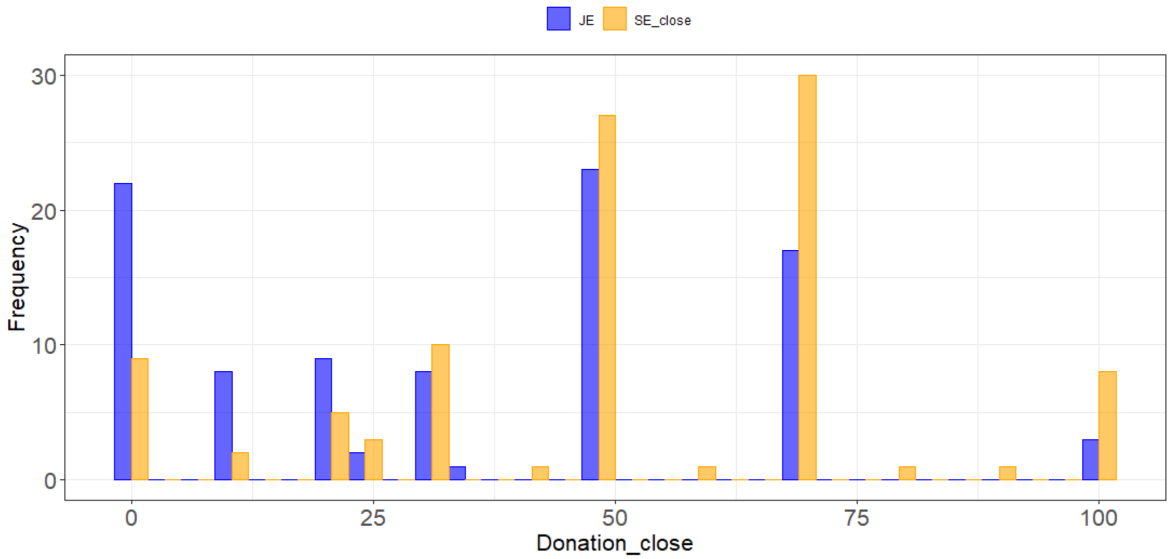

FIGURE W4: DISTRIBUTION OF DONATIONS TO THE CHARITY FARTHER FROM ITS GOAL IN STUDY 1

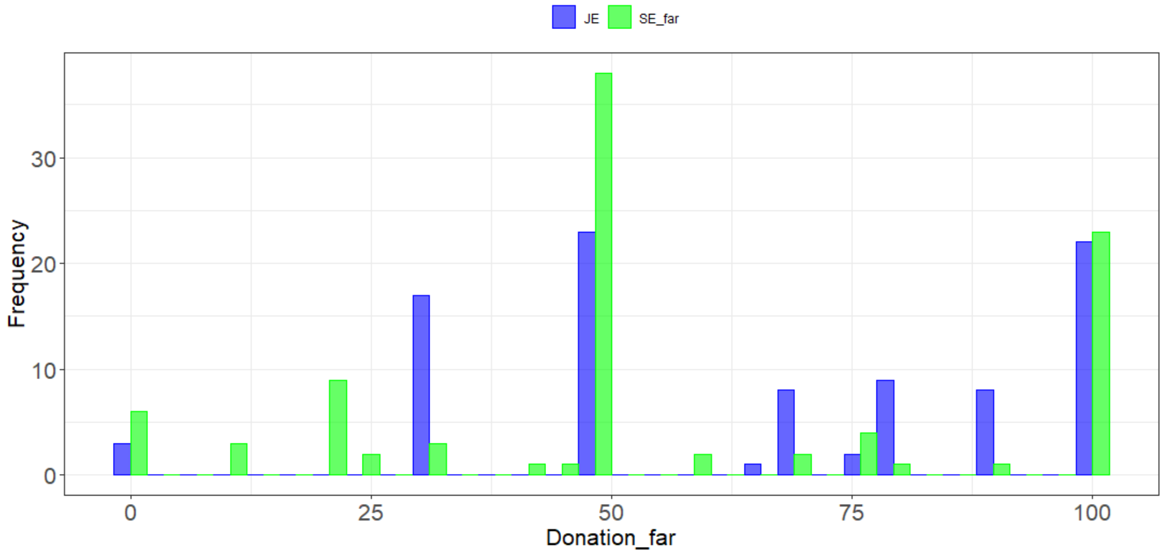

Study 2

FIGURE W5: DISTRIBUTION OF LIKELIHOOD OF DONATING TO THE CHARITY CLOSER TO ITS GOAL IN STUDY 2

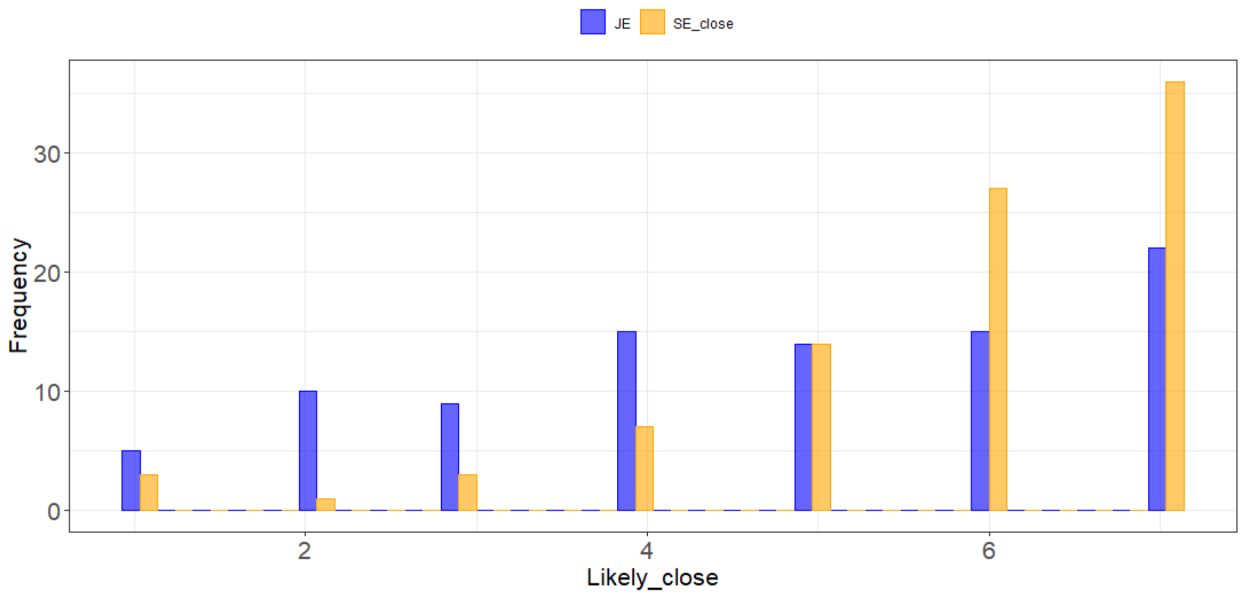

FIGURE W6: DISTRIBUTION OF LIKELIHOOD OF DONATING TO THE CHARITY FARTHER FROM ITS GOAL IN STUDY 2

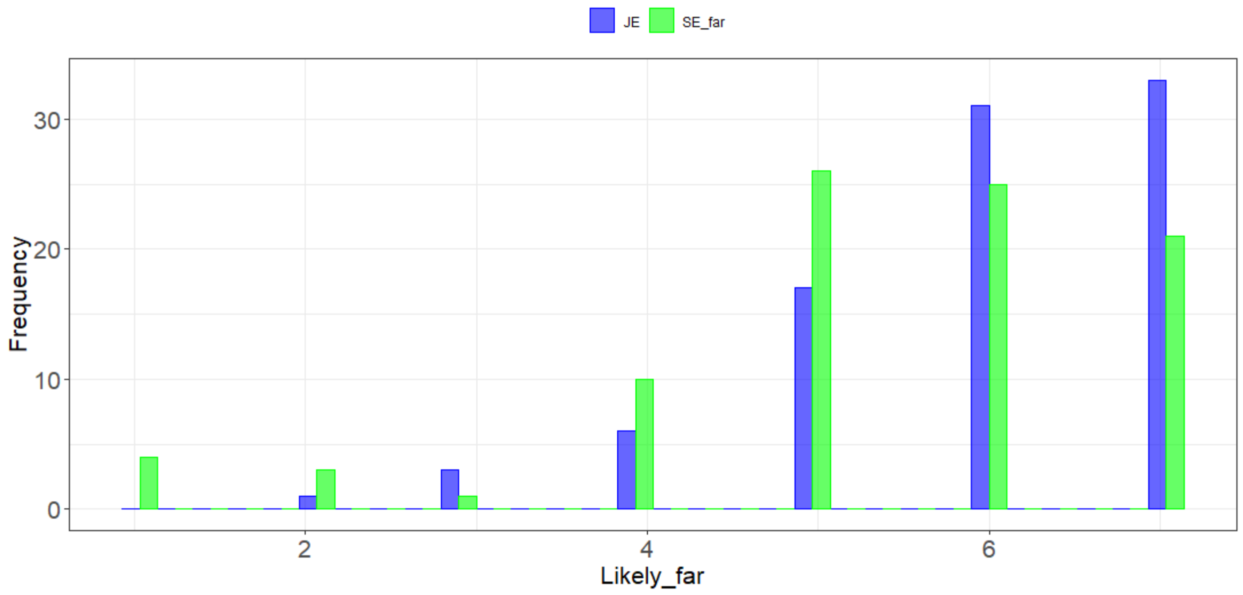

Study 4

FIGURE W7: DISTRIBUTION OF DONATIONS TO THE CHARITY FARTHER FROM ITS GOAL IN STUDY 4

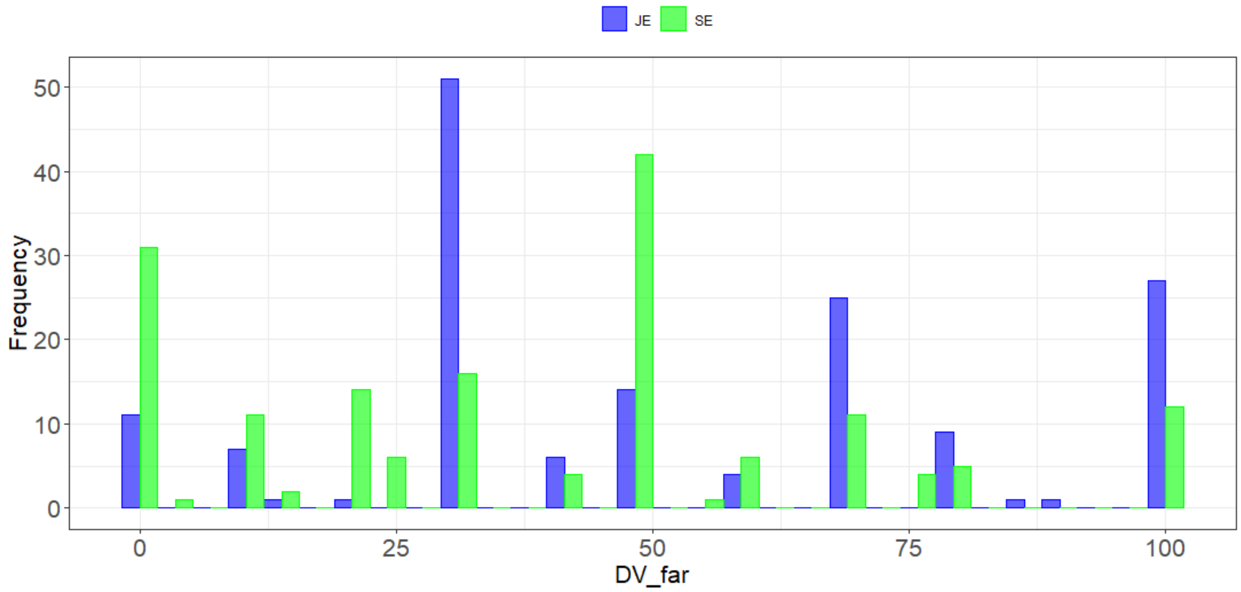

FIGURE W8: DISTRIBUTION OF DONATIONS TO THE CHARITY IN SE IN STUDY 4

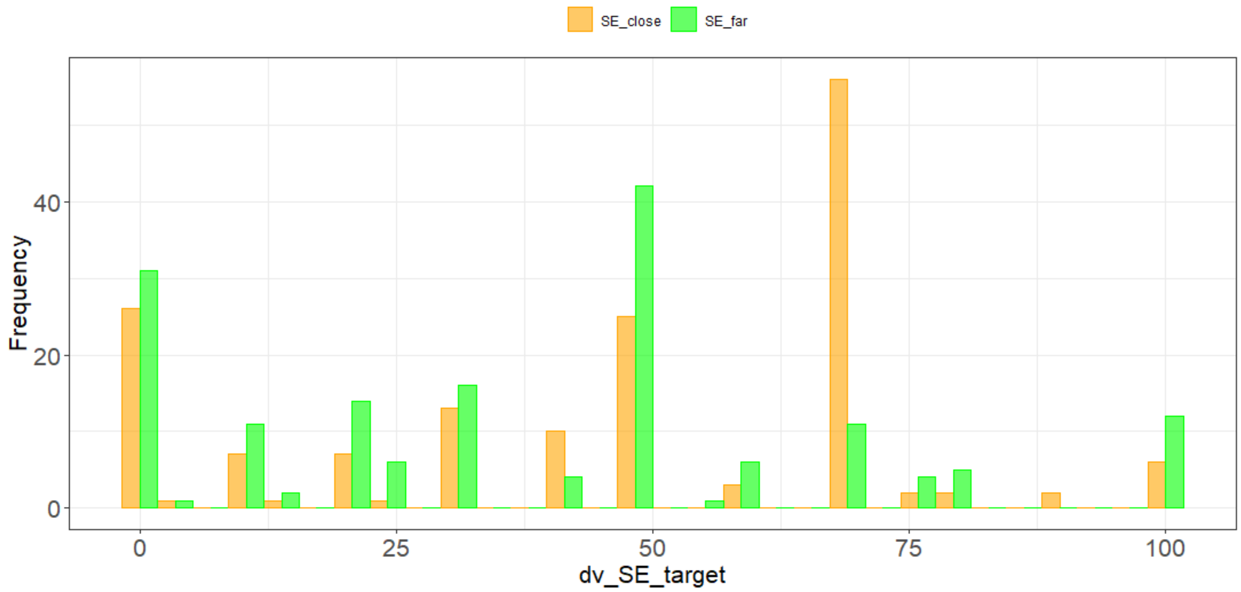

Study 5A

FIGURE W9: DISTRIBUTION OF DONATIONS TO THE CHARITY CLOSER TO ITS GOAL IN STUDY 5A

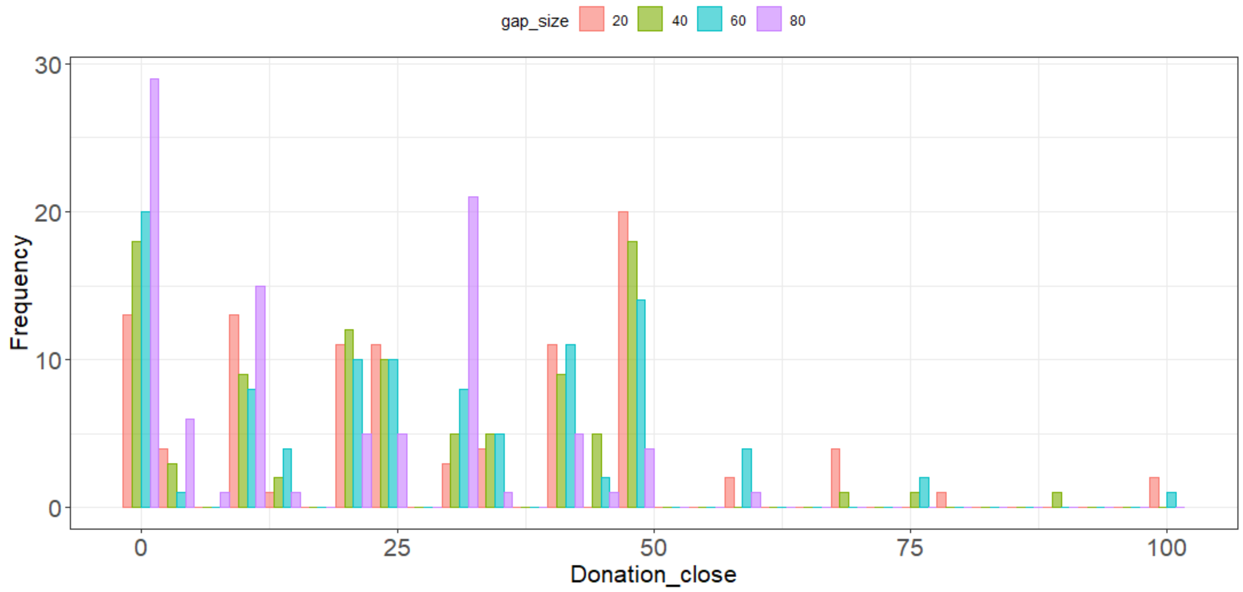

FIGURE W10: DISTRIBUTION OF DONATIONS TO THE CHARITY FARTHER FROM ITS GOAL IN STUDY 5A

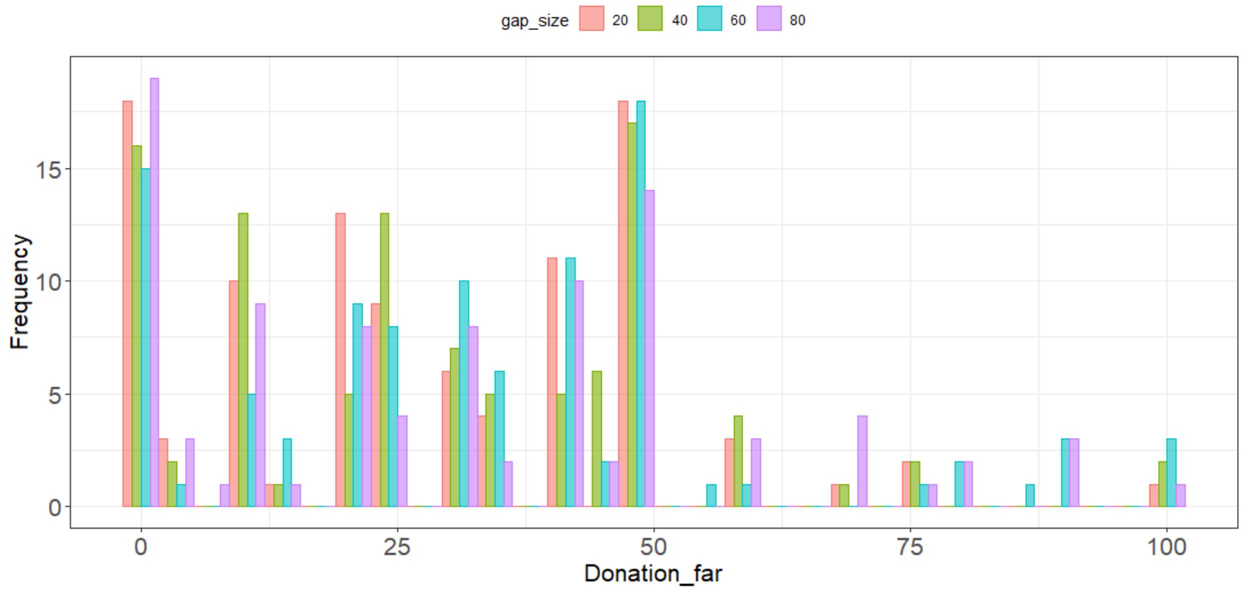

Study 5B

FIGURE W11: DISTRIBUTION OF DONATIONS TO THE CHARITY CLOSER TO ITS GOAL IN STUDY 5B

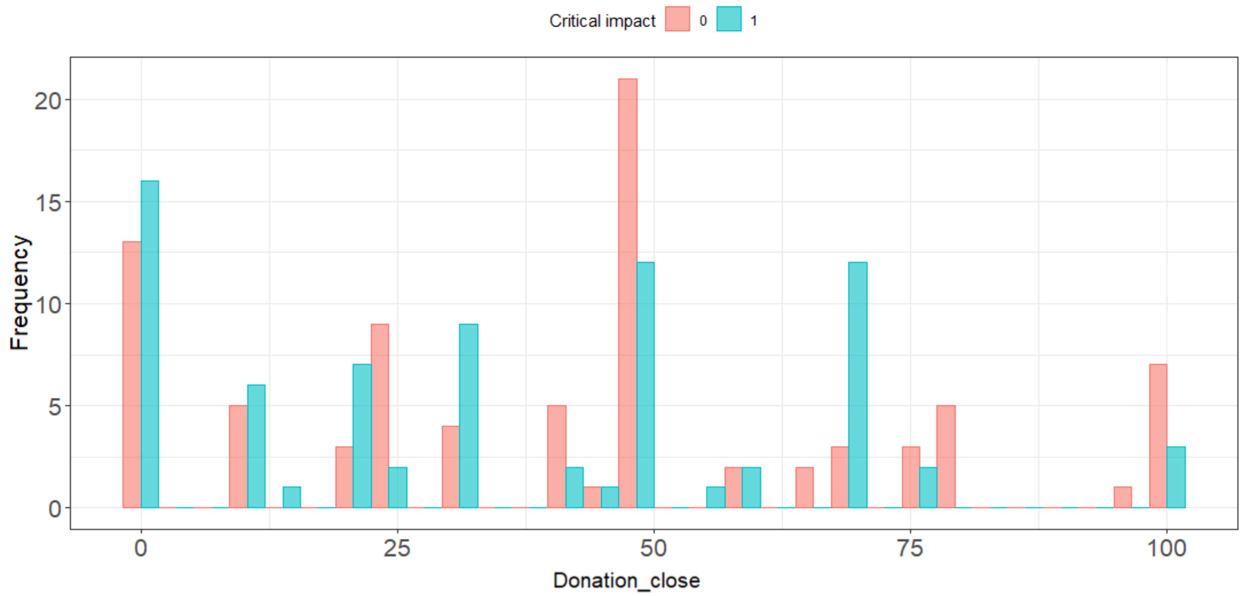

FIGURE W12: DISTRIBUTION OF DONATIONS TO THE CHARITY FARTHER FROM ITS GOAL IN STUDY 5B

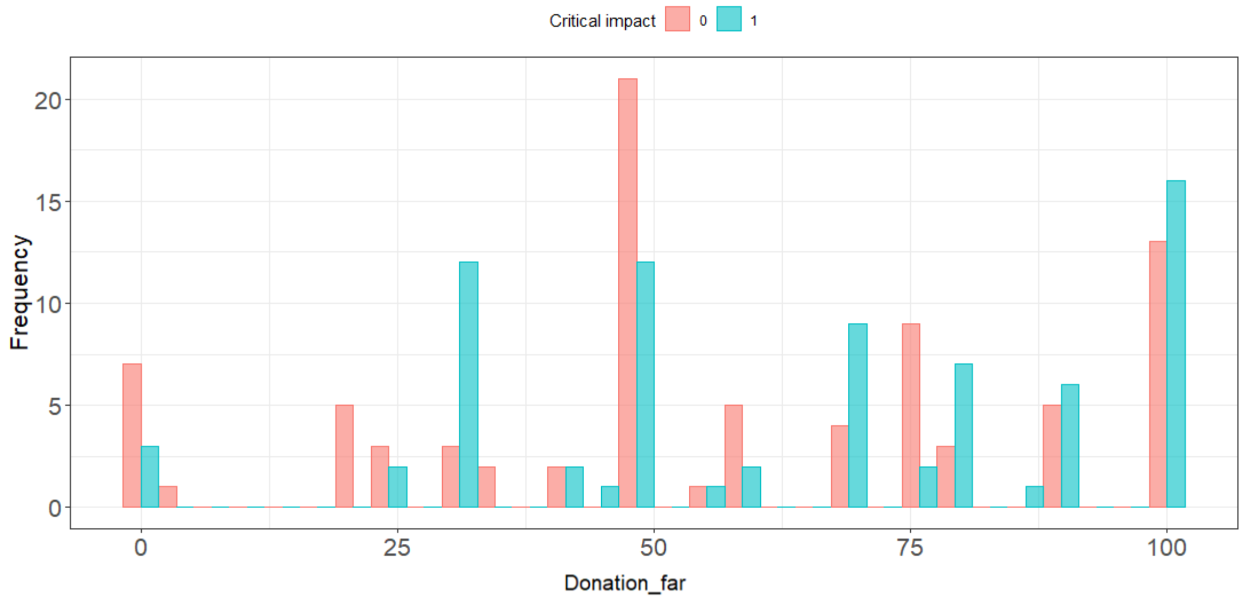

Means for Additional Variables

TABLE W13: MEANS FOR ADDITIONAL VARIABLES ACROSS STUDY CONDITIONS

Means for Additional Variables Across Study Conditions

Study 1 *JE vs SE Charity*

	JE Close	JE Far	SE Close	SE Far
Need	4.35	6.17	4.65	5.60
Impact	4.80	5.75	4.86	4.99
Warmth	5.38	5.41	5.04	5.19
Competence	5.25	4.84	4.85	4.73

Study 2 *JE vs SE Individual*

	JE Close	JE Far	SE Close	SE Far
Need	3.99	6.01	5.00	4.89
Impact	5.26	4.92	5.06	4.60
Sympathy	4.18	5.12	4.54	4.36
Satisfaction	5.18	5.52	5.54	5.18

Study 3 *Three Projects JE vs. SE*

	JE			SE		
	Close	Mid	Far	Close	Mid	Far
Need	4.70	5.38	5.92	5.59	5.48	5.84
Evaluability	4.95	4.71	4.52	4.41	4.53	4.65
Justifiability	5.41			5.29	5.31	5.20

Study 4 *Charity vs Business*

	Charity				Business			
	JE		SE		JE		SE	
	Close	Far	Close	Far	Close	Far	Close	Far
Need	4.47	5.32	4.16	4.13	4.23	5.15	3.67	4.25
Impact	5.04	5.05	4.39	4.22	4.73	4.29	3.81	3.78
Competence	5.25	4.88	4.91	4.67	5.17	4.75	4.60	4.57
Warmth	4.73	4.61	4.28	4.21	4.44	4.18	4.10	4.13

Study 5A *Gap size*

	80%	60%	40%	20%
Need	4.65	4.38	4.43	4.07
Fairness	4.28	4.61	4.86	4.99
Effort	2.89	2.95	3.18	3.09

Study 5B *Tipping Point*

	High progress ($2700 out of $3000)		Tipping point ($2930 out of $3000)	
	SH Close to Goal	SH Far from Goal	SH Close to Goal	SH Far from Goal
Need	2.07	5.14	2.06	5.18
Competence	4.09	3.39	4.35	3.04
Warmth	3.99	3.58	4.17	3.48

Goal Progress Levels Used Across Studies

TABLE W14: LEVEL OF GOAL PROGRESS ACROSS STUDIES

**Level of Goal Progress Across Studies**					
	Closer		Farther		Closing-the-Gap Effect
**Study 1** *JE vs SE Charity*	£2930 out of 3000	98%	£300 out of 3000	10%	Yes
**Study 2** *JE vs SE Individual*	98 out of 100	98%	68 out of 100	68%	Yes
**Study 3** *Three Projects JE vs. SE*	£270 out of 300	90%	£30 out of 300	10%	Yes
	£150 out of 300	50%	£30 out of 300	10%	Yes
**Study 4** *Charity vs Business*	$2730 out of $3000	98%	$300 out of $3000	10%	Yes
**Study 5A** *Gap size*	£270 out of 300	90%	£30 out of 300	10%	Yes
	£240 out of 300	80%	£60 out of 300	20%	Yes
	£210 out of 300	70%	£90 out of 300	30%	No
	£180 out of 300	60%	£120 out of 300	40%	No
**Study 5B** *Tipping Point*	£2700 out of 3000	90%	£300 out of 3000	10%	Yes
	£2930 out of 3000	98%	£300 out of 3000	10%	Yes
**SS1** *JE vs SE Charity*	£2930 out of 3000	98%	£300 out of 3000	10%	Yes
**SS2** *JE vs SE Individual*	98 out of 100	98%	68 out of 100	68%	Yes
**SS3** *Three Projects in JE*	£270 out of 300	90%	£30 out of 300	10%	Yes
	£150 out of 300	50%	£30 out of 300	10%	Yes
	£240 out of 300	80%	£30 out of 300	10%	Yes
	£60 out of 300	20%	£30 out of 300	10%	Yes
**SS4** *Main Effect*	£2700 out of 3000	90%	£300 out of 3000	10%	Yes
**SS5** *Completion contingent Tipping Point*	£240 out of 300	80%	£60 out of 300	20%	Yes
**SS6** *Relative vs. Consistent*	$2700 out of 3000	90%	$300 out of 3000	10%	Yes
	$2700 out of 5400	50%	$300 out of 5400	5.6%	Yes
	$2700 out of 5400	50%	$300 out of 600	50%	No
	$2700 out of 27000	10%	$300 out of 3000	10%	No
**SS7** *To-go vs. To-date*	£245 out of 300	82%	£55 out of 300	18%	Yes

List of Links to Preregistrations

TABLE W15: LINKS TO PREREGISTRATIONS FOR EACH STUDY

**Study**	**Link to Preregistration**
**Study 1** *JE vs SE Charity*	https://osf.io/ym4nu
**Study 2** *JE vs SE Individual*	https://osf.io/nyevm
**Study 3** *Three Projects JE vs. SE*	https://aspredicted.org/T33_6B7
**Study 4** *Charity vs Business*	https://osf.io/jvpz2
**Study 5A** *Gap size*	https://aspredicted.org/GGM_NMX
**Study 5B** *Tipping Point*	https://osf.io/zh2ue
**SS1** *JE vs SE Charity*	NA
**SS2** *JE vs SE Individual*	https://osf.io/7ydh5
**SS3** *Three Projects in JE*	https://aspredicted.org/L7K_RW4
**SS4** *Main Effect*	https://osf.io/b5ryc
**SS5** *Completion contingent Tipping Point*	https://aspredicted.org/TFJ_KS2
**SS6** *Relative vs. Consistent*	NA
**SS7** *To-go vs. To-date*	https://aspredicted.org/RGV_8WD

Mediation models

FIGURE W13: MEDIATION OF DISTANCE FROM GOAL ON DONATIONS THROUGH PERCEIVED NEED IN JE IN STUDY 2

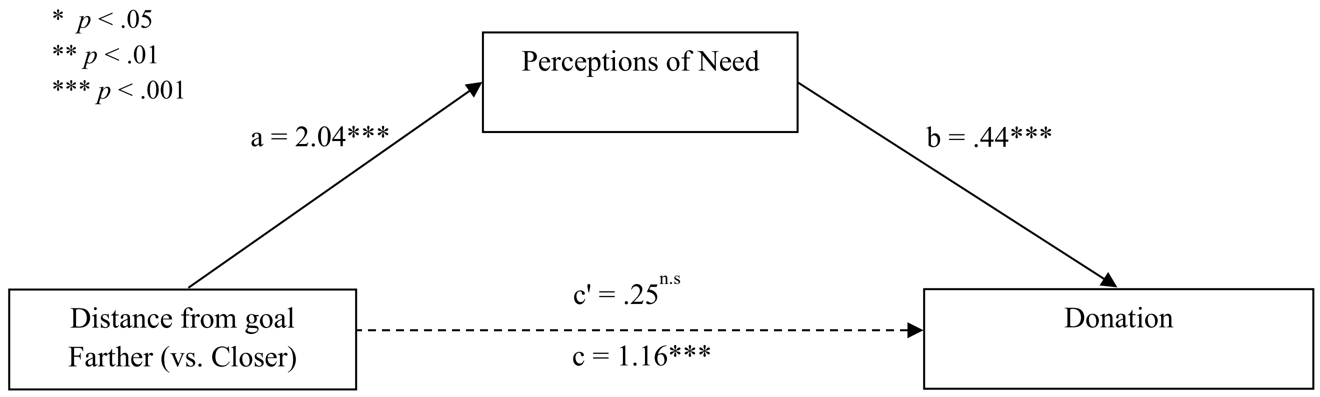

FIGURE W14: MEDIATION OF DISTANCE FROM GOAL ON DONATIONS THROUGH PERCEIVED NEED IN SE IN STUDY 2

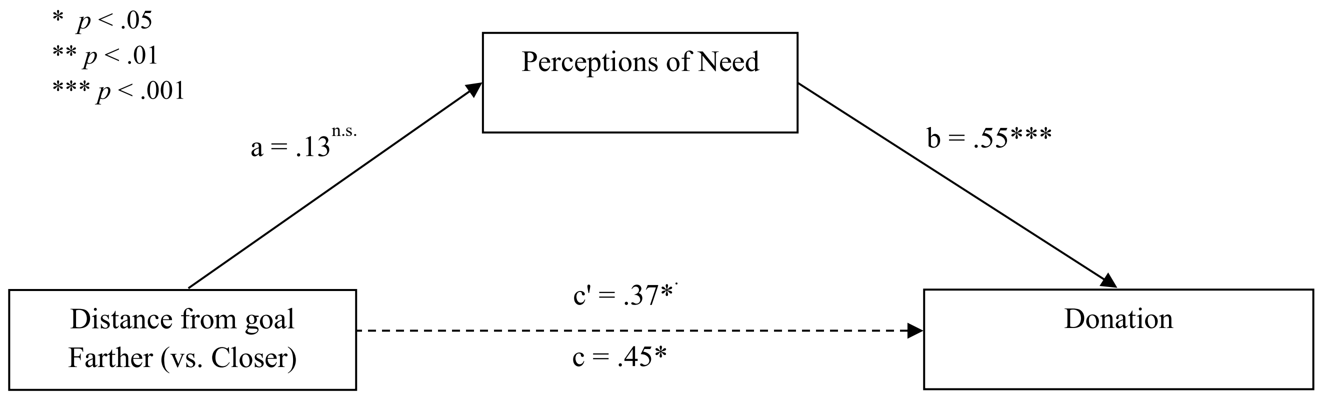

FIGURE W15: MEDIATION OF JE VS. SE ON DONATIONS THROUGH PERCEIVED NEED FOR CHARITIES IN STUDY 4

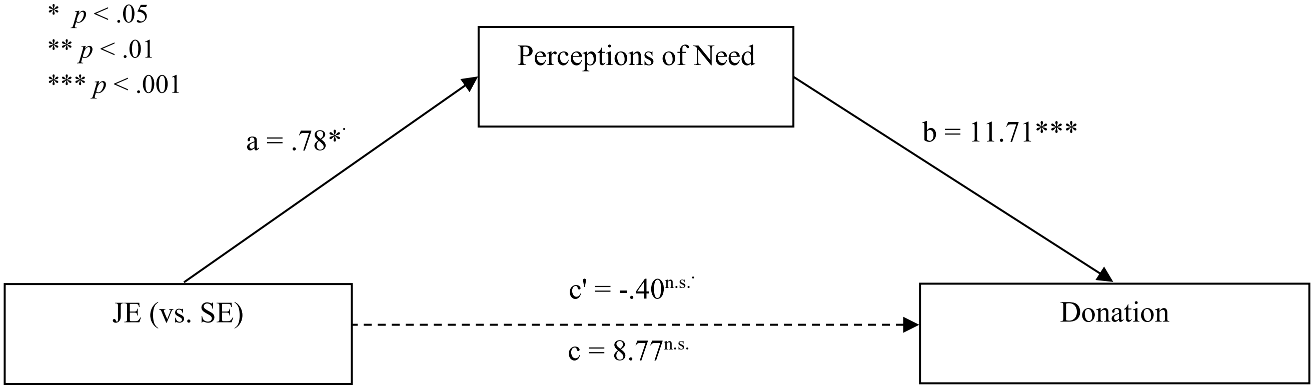

WEB APPENDIX C

Study 5A: Effect of Gap size

This study delves into joint evaluation mode with different combinations of goal progress and manipulates the gap size between the charity further from and closer to its goal to further test the generalizability of our effect.

Method

Participants and design. We recruited 400 US participants from Prolific and after excluding those who failed an attention check asking them to select both "always" and "never", we were left with 394 participants (48.2% Women, M_{age} = 38.29, SD_{age} = 14.15). In previous studies we found that participants chose to give more to a charity further from its goal. In this study we test how the size of the gap between the charity closer to vs. farther from the goal affects the size of this effect found in previous studies. We tested four different gap sizes (80% vs 60% vs. 40% vs. 20% gap) in a between-subject design. This study was preregistered on AsPredicted.

Procedures. Participants were informed at the start of the study that we were collecting money for UNICEF projects and that they would be shown two charity projects on the next page. They saw both the "Donate to buy a water pump" and "Donate to buy vaccines" projects from the previous study at different distances from their goal. Participants were assigned to one of four conditions with varying gap sizes between the charity closer to versus farther from its goal (80% vs 60% vs. 40% vs, 20% gap) as shown in the table below. This meant that in each condition, participants read about 2 charity projects from UNICEF at different levels of goal progress: 1) 10% and 90%, 2) 20% and 80%, 3) 30% and 70%, and 4) 40% and 60%.

TABLE W16: CONDITIONS IN STUDY 5A

Gap size	Progress for project *closer* to goal	Progress for project *farther* from goal
80%	90%	10%
60%	80%	20%
40%	70%	30%
20%	60%	40%

After reading about the charities, participants were informed that one participant would be selected in a random draw after the study was completed, and a £100 prize would be distributed between the two charities and themselves according to their choice. This distribution was our key dependent variable. After the study ended, the money was distributed as per the choices of one randomly selected participant. We also asked participants their likelihood of

donating to each charity separately on a scale of 1 – very unlikely to 7 – very likely. Participants then rated their motivations behind their decision in terms of fairness ("My decision was based on fairness"), need ("My decision was based on how much the organization was in need") and effort ("My decision was based on how much work was put into raising money") using a scale of 1 - Strongly disagree and 7 - Strongly agree. They also rated the information they saw in terms of evaluability (e.g., "If an organization has £X left to raise out of £300, do you have any idea how in need they are?", 1 - I don't have any idea and 7 – I have a clear idea) and justifiability ("When making donation decisions, do you think you should consider how much the organization is in need?", 1 - Should not consider and 7 - Should definitely consider). Lastly, participants completed an attention check asking them to select both always and never and answered basic demographic questions including age, gender, income, and ethnicity.

Results

Amount. Overall, participants kept £44.02 for themselves and donated and average of £55.98. Participants donated significantly more to the charity farther from its goal (M = £30.65, SD = 23.76) than the one closer to its goal (M = £25.32, SD = 20.60), t(393) = 3.85, $p < .001$, d = .19. This was the case when the gap was 80%, (M_{far} = £31.06, M_{close} = £16.80), t(94) = 5.46, $p <$ .001, and when the gap was 60%, (M_{far} = £34.25, M_{close} = £27.23), t(99) = 2.23, $p = .03$. However, there were no differences in donations to the two charity projects when the gap was smaller at 40%, (M_{far} = £29.65, M_{close} = £26.88), t(99) = 1.15, $p = .25$, or 20%, (M_{far} = £27.66, M_{close} = £29.98), t(99) = .90, $p = .37$.

We also ran a regression analysis predicting the difference in donation amount (i.e., donations to the project farther from goal - donations to the project closer to goal) from the size of the gap in goal progress. The model was significant, $R^2 = .05$, F(1, 392) = 19.70, $p < .001$, and revealed a significant positive effect of gap size on extra donations to the project farther from its goal, $b = .22$, t(392) = 4.44, $p < .001$. This indicates that as the gap in goal progress increases, donations to the project further from its goal compared to the one closer to its goal increases and as the gap in goal progress decreases, donations to the project further from its goal becomes similar to the one closer to its goal, attenuating our main hypothesis.

Likelihood. Participants were more likely to donate to the charity farther from its goal (M = 5.02, SD = 1.88) than the one closer to its goal (M = 4.84, SD = 1.90), t(389) = 2.09, $p = .04$, d = .10. This was the case when the gap was 80%, (M_{far} = 4.86, M_{close} = 4.36), t(95) = 2.58, $p =$ .01. However, there were no differences in donation likelihood when the gap was 60%, (M_{far} = 5.42, M_{close} = 5.21), t(99) = 1.27, $p = .21$, or 40%, (M_{far} = 4.85, M_{close} = 4.68), t(97) = .93, $p =$ .35, or when the gap was 20% (M_{far} = 4.93, M_{close} = 5.07), t(96) = .92, $p = .36$.

A regression analysis predicting the difference in donation likelihood from the size of the gap in goal progress found a significant overall model, $R^2 = .01$, F(1, 388) = 6.51, $p = .01$. This revealed a significant positive effect of gap size on higher likelihood to donate to the project farther from its goal, $b = .13$, t(388) = 2.55, $p = .01$. This suggests that as gap size increases,

participants were more likely to donate to the charity further from its goal compared to the one closer to its goal.

Study 5B: Testing the Tipping Point

This study tests the robustness of our effect by pitting it against a strong test of the goal gradient hypothesis. Previous work demonstrates that the goal gradient hypothesis is stronger and donors are more likely to give to a project closer to its goal when they can be the one to complete the goal, sometimes referred to as being the tipping point (Anik and Norton 2020; Argo et al. 2020; Wash 2013). If the goal gradient hypothesis drives decisions in joint evaluations, we would expect donations to shift to the organization closer to its goal when it is at a tipping point and the donor can complete the goal. This study was pre-registered at https://osf.io/zh2ue/?view_only=7ea2e726eac747218df0a1d80382ae4d. We initially predicted (and pre-registered) that the tipping-point condition would reverse our effects and that participants would donate more to the charity closer to the goal in this condition. Yet surprisingly, the results of this study indicate that in the charitable context, people focus on closing the gap between the two charities and still donate more to the charity farther from its goal even when the other one is at its tipping point, thus illustrating the robustness of the focal effect. The study used real charities in a consequential design where participant choices affect actual donations to their selected charitable organization.

Method

Participants and design. We recruited 201 Amazon Mechanical Turk participants from the US (42.3% female, M_{age} = 35.00) in exchange for payment. The study was a one-factor, two-level (Goal progress: high progress vs. tipping point) between-participants design.

Procedures. Participants were shown two similar organizations, FoodShare and Second Harvest from Study 1. Participants were randomly assigned to either the high-progress or tipping-point condition. In both conditions, the organization farther from its goal had raised only $300 out of a goal of $3000 (i.e., 10%). In the high-progress condition, the organization closer to its goal had raised 90%: $2700 out of $3000. In the tipping-point condition, the organization closer to its goal had raised 98% of its goal, or $2930 out of $3000, and thus needed only $70 to complete its goal.

After reading about the organizations, participants were informed that there would be a consequential draw at the end of the study with the winner's allocation of the $100 being given to the two charities. Participants could thus give $70 of $100 to the charity at its tipping point to complete the goal. This donation served as our key dependent variable, and the money was distributed after the survey ended based on the selected participant's choice. Participants then rated the charities in terms of need ("Which organization did you think was more in need?"), impact ("At which organization do you think your donation would make a bigger impact?"), competence ("Which organization did you think was more competent/ capable/ skillful/efficient?"), and warmth ("Which organization did you think was more warm/ kind/

friendly/ sincere?") on 7-point scales ranging from 1- Definitely Second Harvest to 7 – Definitely FoodShare (this corresponded with the left-right positioning of the organization). They then completed a manipulation check asking them which organization was closer to reaching its goal and answered demographic questions including age, gender, and income.

Results

Data preparation. After excluding participants who failed the attention checks we were left with 160 participants in the final analyses (45% female, M_{age} = 35.41). The results are consistent without exclusions and are available below.

Donation. A one-sample t-test revealed that, overall, donations to the organization that was farther from its goal (M = \$60.28, SD = \$29.44) were greater than the midpoint, i.e., \$50, M_{diff} = 10.28, t(159) = 4.42, $p < .001$, d = 0.32. This replicates our results from Study 1 in a context with actual giving, showing that charities farther from their goal receive more donations.

A one-way ANOVA revealed that donations to the charity farther from its goal were directionally higher in the tipping-point condition (M = \$64.14, SD = \$28.47) than the high-progress condition (M = \$56.79, SD = \$30.03), F(1, 158) = 2.52, $p = .11$, d = 0.25. Contrary to predictions from the goal gradient hypothesis, we did not find that participants donated more to help an organization complete its goal at the tipping point. In both conditions we found a consistent tendency to donate to the charity that was farther from its goal—this was directionally stronger in the tipping-point condition where the difference between progress levels was greater.

FIGURE W16: MEDIATION ANALYSIS OF DISTANCE FROM GOAL ON DONATIONS THROUGH PERCEPTIONS OF NEED

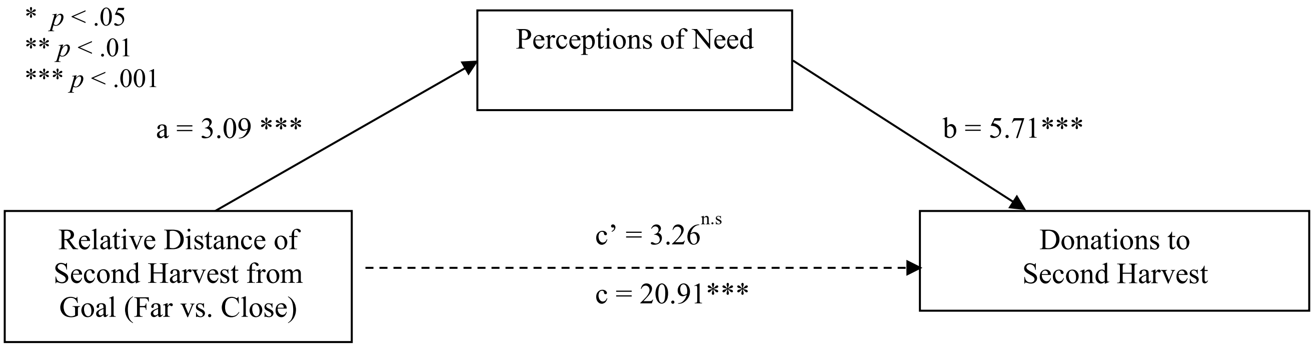

Mediation. We carried out mediation analysis using PROCESS model 4 and 5,000 bootstrap samples (Hayes 2013) to test the effect of distance from goal (dummy coded as 0 = FoodShare farther from goal and 1 = Second Harvest farther from goal) on donations to Second Harvest through perceived need (higher values indicate Second Harvest was perceived as more needy). This revealed a significant indirect effect of need, where perceptions of need mediated the relationship between distance from goal and donations to the charity—when Second Harvest was farther from its goal, it was also perceived as more needy and this led to greater donations to the charity, B = 17.65, SE = 3.62, 95% CI [10.7822, 25.1074] (see Figure W16). Multiple mediation analysis including perceptions of need, warmth, competence, impact, and number of

donors as potential mediators revealed only a significant indirect effect of need, B = 14.43, SE = 4.88, 95% CI [5.3252, 24.5221], on the effect of distance from goal on donations to the charity.

Analyses without Exclusions

Donation: A one-sample t-test revealed that, overall, donations to the organization that was farther from its goal (M = \$58.56, SD = \$27.80) were greater than the midpoint, i.e., \$50, M_{diff} = 8.56, t(200) = 4.37, $p < .001$, d = 0.30.

A one-way ANOVA tested the impact of goal-progress condition (high progress vs. tipping point) on donations to the charity farther from its goal, which were higher in the tipping-point condition (M = \$62.47, SD = \$26.82) than the high-progress condition (M = \$54.75, SD = \$28.33), F(1, 199) = 3.93, $p = .049$, d = 0.28. Contrary to predictions from the goal gradient hypothesis, we did not find that participants donated more to help an organization complete its goal in the tipping-point condition than in the high-progress condition. In both conditions we found a consistent tendency to donate to the charity that was farther from its goal, and this was even stronger in the tipping-point condition where the gap was larger (see figure W17).

FIGURE W17: DONATION AMOUNT BASED ON TIPPING-POINT AND HIGH-PROGRESS CONDITIONS

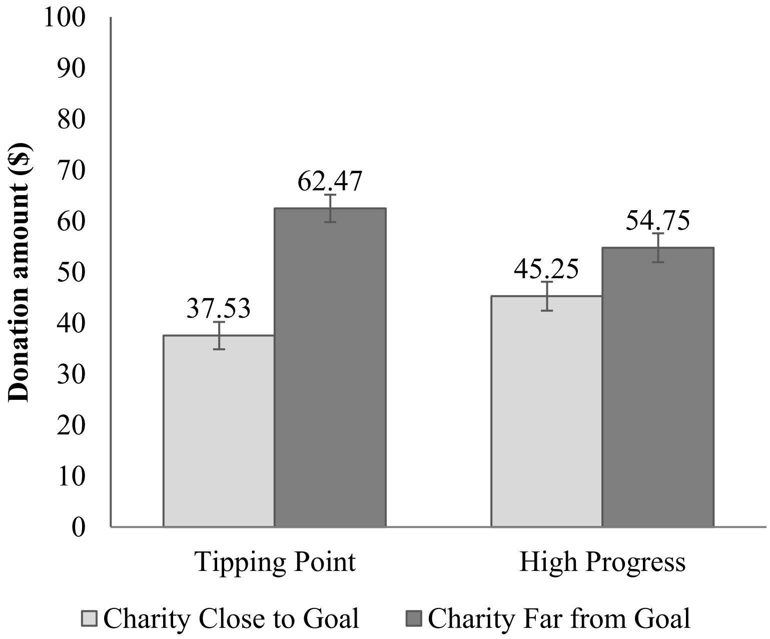

Mediation. We carried out mediation analysis using PROCESS model 4 and 5,000 bootstrap samples (Hayes 2013) to test the effect of distance from goal (dummy coded as 0 = FoodShare farther from goal and 1 = Second Harvest farther from goal) on donations to Second Harvest through perceived need (higher values indicate that Second Harvest was perceived as more needy). This revealed a significant indirect effect of need, where perceptions of need mediated the relationship between distance from goal and donations to the charity; when Second

Harvest was farther from its goal, it was also perceived as more needy and this led to greater donations to the charity, B = 12.58, SE = 2.75, 95% CI [7.5118, 18.4375] (see figure W18).

Multiple-mediation analysis including perceptions of need, warmth, competence, impact, and number of donors as potential mediators revealed only a significant indirect effect of need, B = 9.35, SE = 3.71, 95% CI [2.6555, 17.1736] on the relationship between distance from goal and donations to the charity.

FIGURE W18: MEDIATION ANALYSIS OF DISTANCE FROM GOAL ON DONATIONS THROUGH PERCEPTIONS OF NEED.

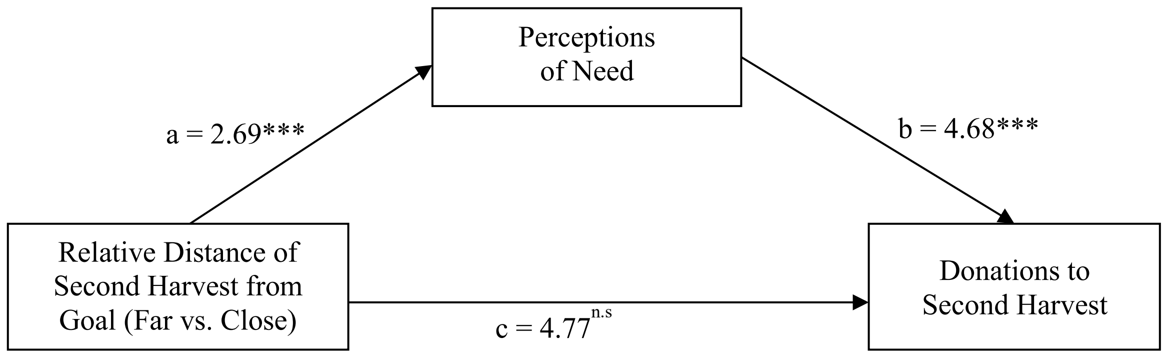

Supplemental Study S1: Replication of JE vs. SE charities

Method. This study replicates Study 1 with 293 undergraduate students (59% female, M_{age} = 19.92) from a large North American university who participated in exchange for course credit. The study was a 3-factor (Evaluation type: joint vs. separate far vs. separate close) between-subjects design. Participants were presented with information about an organization close to its goal (SE close), an organization far from its goal (SE far), or both simultaneously (JE). The organization closer to the goal had raised $2930 out of $3000 and the organization farther from the goal had raised $300 out of $3000. They then indicated how they would divide $100 between the two charities they read about in joint evaluations (JE) or between the charity they read about and another charity of their choice in separate evaluations (SE). As in previous studies, they rated the organization(s) in terms of need, impact, competence, and warmth.

Results. A hybrid t-test as proposed by Hsee (1996) to analyze joint-separate evaluations revealed that respondents were more sensitive in JE than SE to whether an organization was closer to versus farther from its goal, t(290) = 3.31, *p* = .001; in SE, participants donated directionally more to the organization closer to its goal (M = $54.57, SD = 31.66) than the one farther from its goal (M = $48.24, SD = 27.06), t(143) = 1.29, *p* = .20, though this difference was not significant. In joint evaluations, the results were reversed, and participants donated more to the organization that was farther from its goal (M = $56.25, SD = 35.59) than the one closer to its goal (M = $43.75, SD = 35.59), t(147) = 2.14, *p* = .03, d = .35. Contrasts revealed that participants donated more to the organization that was farther from its goal in the joint evaluation than in the separate evaluation condition, M_{diff} = 9.44, t(291) = 2.47, *p* =.01.

FIGURE W19: DONATIONS TO AN ORGANIZATION IN JOINT VS. SEPARATE EVALUATION BASED ON DISTANCE FROM GOAL IN SS1

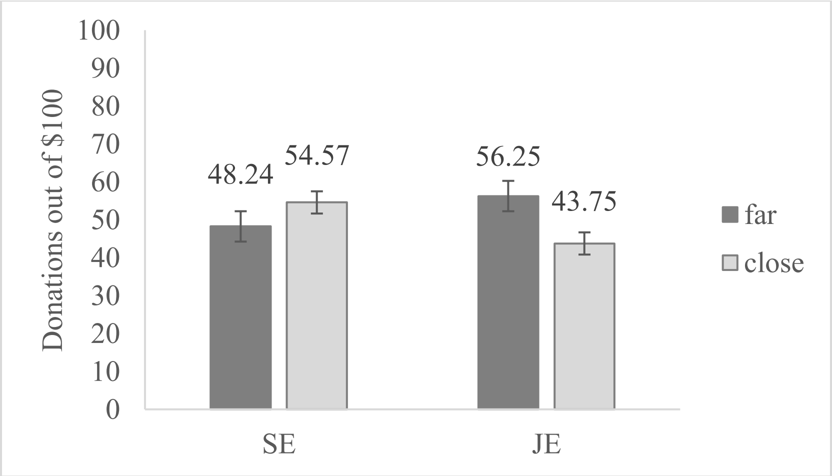

Note: Error bars represent ±1 standard error.

Supplemental Study S2: Replication of JE vs. SE individuals

This study replicates Study 2 using the same scenario from previous work where a student sells candy (Cryder, Loewenstein, and Seltman 2013). This study was preregistered on the Open Science Framework: https://osf.io/7ydh5/?view_only=e2b953e2f527410eb1994cc5170b9eb4.

Method

Participants and design. We recruited 541 participants from the US for a study on Prolific in exchange for payment (51.2% female, M_{age} = 33.52). The study had a 3-factor design (Evaluation type: joint vs. separate far vs. separate close). A total of 519 participants passed an attention check asking them to select both "always" and "never". Of these, 486 correctly answered a question about how many students were in the story they read based on the preregistered exclusion criteria and were included in the following analyses (53.7% female, M_{age} = 32.93).

Procedures. All participants were presented with information about Olivia and/or Sienna (counterbalanced), 7th-grade students who need to sell 100 candy bars (cost: $1 each) to meet a quota for their school sports team fundraiser. The student(s) needed to sell either 2 or 32 more candy bars to meet the goal (stimuli from Cryder, Loewenstein, and Seltman 2013). In the joint evaluation condition both students were presented, and one student was close to her goal (needs to sell 2 more candy bars) while the other was far from her goal (needs to sell 32 more candy bars). In the separate evaluation conditions only one student was presented, either close to or far from her goal, which is exactly like previous research using these scenarios.

Participants then indicated their likelihood of buying a candy bar from each student (1 = not likely at all, to 7 = very likely). They also answered questions on perceived need: "To what extent did X need your help?" (1 = not at all, to 7 = to a great extent); and impact: "How much progress would your potential candy bar purchase make toward X's goal?", "How big would your contribution be toward X's goal if you purchased a candy bar?", and "How substantial would your contribution be toward X's goal if you purchased a candy bar?" (1 = not at all, to 7 = a lot of progress/very big/very substantial). X was replaced with the name of the student(s) in the scenario. Participants also answered 3 items to measure how satisfying it would be to help the student reach her goal: "How satisfying would it be to help X reach her goal?", "How excited would you be to help X reach her goal?", and "How happy would you feel to be able to help X reach her goal?" (1 = not at all, to 7 = very satisfying/excited/happy). An additional 3 items measured sympathy for the student: "To what extent do you feel sympathy for X?", "To what extent do you feel compassion for X?", and "To what extent do you feel distress for X?" (1 = not at all, to 7 = to a great extent). Lastly, they answered 1 item to measure likelihood of reaching the goal: "How likely is it that X will reach her goal?" (1 = not likely at all, to 7 = very likely).

Results

Purchase likelihood. A hybrid t-test revealed a significant shift in preferences between JE and SE on likelihood of buying from the student close to her goal. Respondents were more sensitive in JE than in SE to whether the student was close to or far from her goal, t(483) = 7.33, $p < .001$. We followed this up with analyses for each condition. In JE, a paired-samples t-test showed that respondents were more likely to buy from the student who was farther from her goal (M = 5.92, SD = 1.33) than closer to her goal (M = 4.64, SD = 2.04), t(169) = 7.40, $p < .001$, d = .63. In SE, an independent-samples t-test revealed that there was no difference between respondents' likelihood of buying from the student who was closer (M = 5.88) versus farther (M = 5.66) from her goal, t(314) = 1.24, $p = .22$.

Mediation. To determine the role of perceived need as a mechanism in the joint evaluation condition, we followed Montoya and Hayes' (2017) procedure for mediation analysis with within-subject data using the MEMORE macro for SPSS with 10,000 bootstrap samples. This revealed a significant indirect effect of distance from goal (far vs. close) on likelihood of purchase through perceived need, B = .75, 95% CI [.1596, 1.3819].

Impact did not mediate the effect of distance from goal on likelihood of buying in JE, B = -.07, 95% CI [-.1954, .0836]. Including both need and impact in a parallel multiple-mediation model, we find that need significantly mediated the relationship between distance from goal and likelihood of donations to the student farther from her goal, B = .76, 95% CI [.1686, 1.3786] but impact did not mediate this relationship, B = -.06, 95% CI [-.1782, .0814].

Discussion

This study uses a situational vignette from previous work to provide additional support for our predictions and replicates study 3. Consistent with our conceptual framework, people were more likely to purchase candy from a student who is farther from a goal than closer to a goal when they evaluated both students jointly.

Supplemental Study S3: Three Projects in JE

Method

Participants and design. We recruited 300 participants from Prolific and after excluding those who failed an attention check asking them to select both "always" and "never" as preregistered, we were left with 291 participants (50.2% Women, $M_{age} = 38.12$, $SD_{age} = 13.05$). In this study we test if donations to the charity farthest from its goal remains highest when three projects are evaluated jointly. As our previous studies had only looked at two projects this helps show the generalizability of the effect. Participants saw three projects jointly at different distances from the goal. The project farthest from the goal had made 10% progress and the one closest to the goal had made 90% progress. We varied the progress of the project in the middle in a 3 factor (low 20% vs. medium 50% vs. high 80% progress) between-subjects design. We counterbalanced which of three projects (Water pump, Vaccine, and Nutrition Center) were at different levels of progress. Participants then distributed $100 between the three organizations they saw and themselves. They then rated their motivation for donating in terms of need, fairness and effort on 7-point scales as in previous studies.

Results

Overall, paired samples t-tests showed that participants donated more to the charity project farthest from its goal (M = 31.38, SD = 28.89) than the one closest to its goal (M = 18.19, SD = 16.57), t(290) = 6.26, $p < .001$, and the project in the middle (M = 22.14, SD = 19.75), t(290) = 4.23, $p < .001$. A one-way ANOVA revealed no effect of condition on donation intentions to the project farthest from its goal, F(2, 288) = .08, $p = .92$, closest to the goal, F(2, 288) = .32, $p = .73$, or the project in the middle, F(2, 288) = .28, $p = .76$.

In each condition, donations were higher to the project farthest from its goal (i.e., at 10% progress). In the 10%/20%/90% condition participants donated more to the charity project farthest from its goal (M = 31.49, SD = 26.90) than the one closest to its goal (M = 18.77, SD = 17.58), t(98) = 3.72, $p < .001$, and the project in the middle (M = 23.16, SD = 19.28), t(98) = 2.43, $p = .02$. In the 10%/50%/90% condition participants donated more to the charity project farthest from its goal (M = 30.48, SD = 28.73) than the one closest to its goal (M = 17.08, SD = 15.14), t(95) = 3.81, $p < .001$, and the project in the middle (M = 22.18, SD = 18.69), t(95) = 2.22, $p = .03$. In the 10%/80%/90% condition participants donated more to the charity project farthest from its goal (M = 32.15, SD = 32.21) than the one closest to its goal (M = 18.71, SD = 16.97), t(95) = 3.34, $p = .001$, and the project in the middle (M = 21.05, SD = 21.35), t(95) = 2.64, $p = .01$.

Paired sample t-tests also revealed that participants were motivated by need (M = 4.58, SD = 1.96) more than effort (M = 2.94, SD = 1.70), t (290) = 12.75, $p < .001$. They were also motivated by fairness (M = 4.40, SD = 1.92) more than effort, t(289) = 12.55, $p < .001$. There were no differences overall between need and fairness motivations, t(289) = 1.40, $p = .16$, though need was directionally higher than fairness. In the 10%/20%/90% condition participants were more strongly motivated by need (M = 4.79, SD = 1.85) than both fairness (M = 4.33, SD = 1.77), t(97) = 2.32, $p = .02$, and effort (M = 2.86, SD = 1.60) , t(98) = 9.54, $p < .001$. In the

10%/50%/90% condition participants were motivated by need (M = 4.49, SD = 2.06) more than effort (M = 3.04, SD = 1.77), t (95) = 5.99, $p < .001$. They were also more motivated by fairness (M = 4.48, SD = 1.90) than effort, t(95) = 7.01, $p < .001$. There were no differences overall between need and fairness motivations in this condition, t(95) = .05, $p = .96$. Lastly, in the 10%/80%/90% condition participants were again motivated by need (M = 4.45, SD = 1.97) more than effort (M = 2.93, SD = 1.74), t (95) = 6.88, $p < .001$. They were also more motivated by fairness (M = 4.40, SD = 2.09) than effort, t(95) = 6.78, $p < .001$. There were no differences overall between need and fairness motivations in this condition, t(95) = .22, $p = .83$. Thus, when we presented three projects simultaneously, need was only a stronger motivator than fairness in the condition where there were two projects at low levels of progress (10% and 20%) and one project at a high level of progress (90%).

Discussion

This study helps show the robustness of our results when three projects are presented simultaneously, by varying the progress levels of the project in the middle. We find that participants continue to give the most to the charity farthest from its goal across various combinations of three projects in joint evaluation.

Supplemental Study S4: Main effect

This study provides a simple test of how people choose to donate when they see two charities at different distances from their goals. We predict that they will tend to give more to the charity farther from its goal. In this study we created names, logos, and descriptions for two fictional charities working in a similar domain to ensure that participants' prior familiarity with and their existing impressions of the charity did not influence their answers. This study was pre-registered.

Method

Participants. Ninety-nine US participants took part in this study on Prolific Academic. After excluding the 2 participants who failed an attention check (i.e., they failed to select "strongly disagree") as per our pre-registration, we were left with 97 participants (39.2% female, M_{age} = 36.44).

Procedure. All participants saw information about two organizations, Children's Food Fund and Nutrition for Kids, and read that "both of them have enough funding to cover fixed expenses so any money you give will go straight to the cause." Participants then saw each charity's logo, a brief description, and each charity's progress towards its goals. One organization was farther from its goal and had raised $300 out of $3000, and the other was closer to its goal and had raised $2700 out of $3000 (see Web Appendix A or OSF for stimuli). In this and subsequent studies the stimuli were counterbalanced to minimize any effects of order or organization name. Specifically, the order of the charities (left vs. right) and name of the charity farther from its goal (in this case, Children's Food Fund or Nutrition for Kids) were counterbalanced to create four different versions of the stimuli.

Participants then indicated their likelihood of donating to the two charities ("Which of the above charities would you be most likely to give to?" 1 = definitely Children's Food Fund, to 7 = definitely Nutrition for Kids). Participants were also asked to imagine that they had $100 left over at the end of the month and to indicate the amount they wished to donate ("How would you like to divide $100 between the two organizations?"). They then answered an attention check asking them to select "strongly disagree" and completed demographic information including gender, age, and income.

Results

Data preparation. Donation likelihood and amount data were recoded such that higher scores represented giving to the charity farther from its goal.

Order effects. There was no effect of presentation order on likelihood of donating, $t(95) = .81$, $p = .42$, or amount given to each charity, $t(95) = .10$, $p = .92$. Thus, we collapsed results across these counterbalanced conditions.

Donation. A one-sample t-test revealed that participants were *more likely* than the midpoint (i.e., 4 on a 7-point scale) to donate to the organization farther from its goal ($M = 4.60$,

SD = 1.99), t(96) = 2.96, *p* = .004, d = 0.30; and that they *donated more* than the midpoint of $50 to the charity farther from its goal (M = $58.14, SD = 28.79), t(96) = 2.79, *p* = .006, d = .28.

Discussion

This study provides a simple demonstration that when two charities are shown together, people report being more willing to donate to and intend to donate more to the one that is farther from its goal. However, this study featured only a single combination of amount raised and total goal amount for the charities closer to and farther from their goals.

Supplemental Study S5: Tipping Point Completion Contingent in JE

This study aimed to test the strength of the tipping point effect. We had previously found our effect when a charity was at the tipping point and there was no mention of the conditions under which the charity would receive the money. Thus, in this study we explicitly informed participants if the charity would receive the money they had raised regardless of whether they reach the goal (i.e., not completion contingent condition) or if they would only receive the money if they reach their goal (i.e., completion contingent condition). This study was pre-registered.

Method. We recruited 300 participants from Prolific (49.3% women, M_{age} = 40.17). They were randomly assigned to one of two conditions, completion contingent vs. not completion contingent, in a between-subjects design. In each condition they read about two charity projects from UNICEF, one of which involved donating to buy a water pump and the other donating to buy vaccines for children (counterbalanced). The charity farther from its goal had raised £60 out of £300 and the one closer to its goal had raised £240 out of £300. In the completion contingent condition participants were informed that "If projects do not reach their funding goals, they will NOT receive money and you will be refunded" while in the completion not contingent condition they were informed that "If projects do not reach their funding goals, they will still receive the amount they have raised". They were also informed that they would be entered into a prize draw to win £100 which they could distribute between the charities they saw and themselves. After seeing the charities they then proceeded to distribute the money. They then answered questions about the motivations behind their donation decision and whether it was based on need, fairness or effort. They also answered two manipulation check questions asking about completion contingency and which of the projects was closer to reaching their goal.

Results

Data preparation. After exclusions based on our pre-registered criteria, 293 participants remained (49.5% women, M_{age} = 40.07).

Donation amount. In the non-completion contingent condition where the charity projects receive money even if they do not reach their goal, we replicate our previous effects; participants

give more to the charity further from its goal (M = 32.62) than closer to its goal (M = 26.81), t(146) = 2.45, $p = .02$. In the completion contingent condition where the charity will *only* receive funds if it reaches its goal there is no difference in donations, t(145) = .75, $p = .45$, between the charity further from its goal (M = 27.69) and closer to its goal (M = 29.60). We also find that donations to the charity further from its goal is marginally higher in the non completion contingent condition, t(291) = 1.75, $p = .08$, compared to the completion contingent condition.

Supplemental Study S6: Relative vs. Consistent goal progress and goal amount

This study explores the robustness of the closing-the-gap effect using different levels of goal progress and goal amount. Based on our conceptual framework, we predict that people will donate more to the charity that has raised less money only when it also has relatively lower levels of goal progress. However, when consumers are given information indicating that both charities have the same consistent levels of goal progress, we predict that donations to both charities will be similar. This is because need is a function of both the target goal and the amount raised, and this is most concisely represented by percentage of progress. Previous work demonstrates that consumers process values, such as discounts, relatively rather than absolutely (Grewal and Marmorstein 1994; Grewal, Marmorstein, and Sharma 1996). For instance, a $10 discount is seen as large when it is for a cheaper product and small when it is for an expensive product, as people calculate the percentage of discount. Thus, we predict that consumers will focus on relative differences between charities' goal progress over absolute amounts raised in order to determine which one is more in need. For example, if one charity has raised 10% of a $1,000 goal, and a second charity has raised 10% of a $100,000 goal, we predict that consumers will perceive them to be roughly equally needy.

We also measure our focal mediator, perceived need, along with several potential alternative explanations. Importantly, we measure perceived impact, as previous research has shown that people give to organizations closer to their goal in order to feel like they are having a greater impact (Jensen, King, and Carcioppolo 2013). In addition, it is possible that greater progress will influence how competent or warm an organization seems. We include measures of both warmth and competence in this study because these are fundamental components of social perception and have been considered especially important in the donation context (Aaker, Vohs, and Mogilner 2010; Fiske, Cuddy, and Glick 2007).

Method

Participants and design. Five hundred Amazon Mechanical Turk participants (48.8% female, $M_{age} = 37.81$) took part in this study in exchange for payment. We varied the goal-progress type in a 2 (Progress Type: relative vs. consistent) between-subjects design with two replicate levels each and an additional control condition with no goal information specified.

Procedures. Participants first read about two organizations involved in distributing food to children, Children's Food Fund (CFF) and Nutrition for Kids (NFK), as in Study 1. The conditions are shown in the table below:

TABLE W17: CONDITIONS IN SS6

**Condition**	Goal for charity that has raised **$300**	Progress level	Goal for charity that has raised **$2700**	Progress level
Control	No information		No information	
Relative Progress Medium	$3000	10%	$3000	90%
Relative Progress Low	$5400	5.6%	$5400	50%
Consistent Progress Medium	$600	50%	$5400	50%
Consistent Progress Low	$3000	10%	$27000	10%

In all conditions the amount raised was the same; the charity that was farther from its goal had raised $300 and the one closer to its goal had raised $2700. This was the only information given in the no-goal-information control condition. In the relative-goal-progress conditions, the goal amount was the same for both organizations, and so their relative progress levels varied. There were two replicates for this condition, with a total goal of either $5400 or $3000. This meant that in the $5400-goal condition the charities were at 5.6% and 50% progress, and in the $3000-goal condition they were at 10% and 90% progress levels.

In the consistent-goal-progress conditions, both charities had the same level of progress towards their goal, but their goal amounts varied. There were two replicates in this condition, with the charities having either 10% or 50% progress. In order to maintain consistent progress levels, we varied the goal amounts for each organization while keeping the amount raised the same. This meant that in the 10%-progress condition the charities had goals of $3000 and $27000, and in the 50%-progress condition they had goals of $600 and $5400. Thus, the percentage of goal progress for both charities was the same regardless of the dollar amount raised.

After reading information about each organization, participants indicated how they would divide a $100 donation between the two organizations. We also asked participants their likelihood of donating to the charities (1 = definitely CFF, to 7 = definitely NFK, counterbalanced). Participants then answered questions about their perceptions of the organizations in terms of need ("Which organization did you think was more in need?"), impact ("At which organization do you think your donation would make a bigger impact?"), competence ("Which organization did you think was more competent/capable/skillful/efficient?"), and warmth ("Which organization did you think was more warm/kind/friendly/sincere?") on a scale of 1 = definitely CFF, to 7 = definitely NFK (counterbalanced). Participants then answered manipulation checks about which organization had raised more money, had a higher goal, and

greater goal progress. Lastly participants completed demographic information including gender, age, income, and ethnicity.

Results

Order effects. We tested to see if there was any effect of order (left vs. right) on likelihood of giving and amount given. We found that participants were equally likely to give, $F(3, 496) = 0.13$, $p = .94$, and donated the same amount, $F(3, 496) = 1.89$, $p = .13$, regardless of order. Thus, we collapsed across order in our analyses.

Replicates. Next, we looked to see if there were any differences between the two replicates for each progress-type condition. In the relative-progress condition, there was no difference between the two replicates on likelihood of donating, $t(198) = 0.33$, $p = .74$, and amount donated, $t(198) = 0.90$, $p = .37$, to the organization that had raised less money. Similarly, in the consistent-goal-progress conditions, there was no difference between the two replicates in likelihood of donating, $t(199) = 0.50$, $p = .62$, and amount donated, $t(199) = 0.01$, $p = .996$, to the organization that had raised less money. We therefore collapsed across the two progress-level conditions when conducting the remaining analyses.

Manipulation checks. Overall, 81.6% of participants (N = 408) correctly chose the organization that had raised more money. We found no effect of order and no difference between replicate conditions, and so we collapsed across replicate conditions.

FIGURE W20: DONATION AMOUNT BASED ON AMOUNT RAISED IN SS3

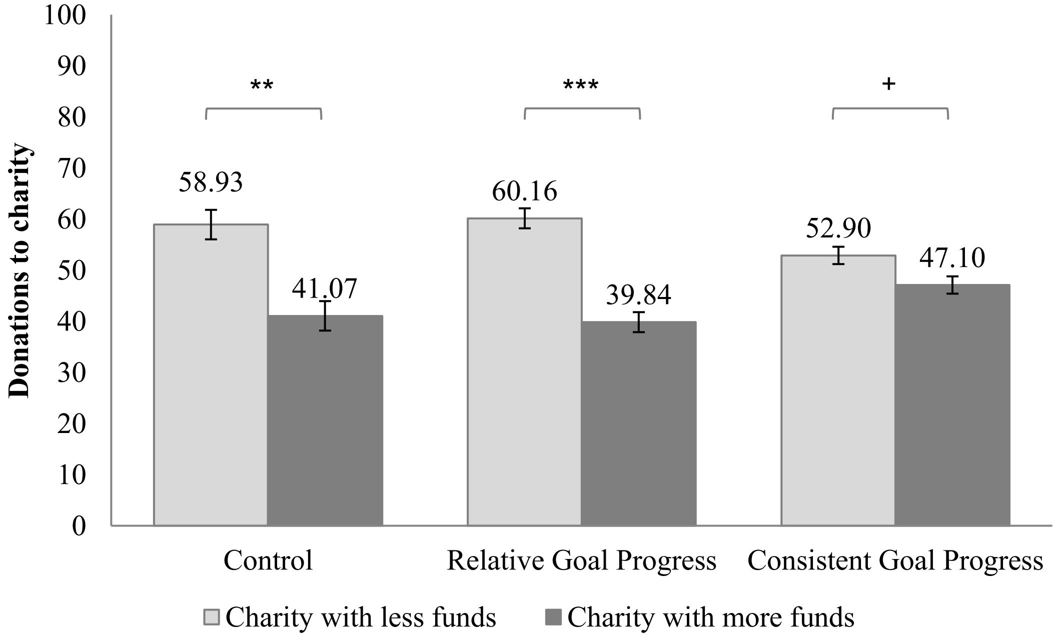

Note: Error bars represent ±1 standard error. $^{+}p < .10$, * $p < .05$, ** $p < .01$, *** $p < .001$

Donation amount. A one-way ANOVA revealed a significant difference in donations to the charity farther from its goal among the control, relative-, and consistent-goal-progress conditions, F(2, 497) = 4.10, *p* = .02. Follow-up contrasts showed that donations were higher for the relative-goal-progress condition (M = 60.16, SD = 27.66) than the consistent-progress condition (M = 52.90, SD = 24.02), t(497) = 2.75, *p* = .006. Similarly, donations were marginally higher for the control condition (M = 58.93, SD = 28.71) than the consistent-progress condition, t(497) = 1.86, *p* = .06. There were no differences between the control and relative-goal-progress conditions, t(497) = .38, *p* = .71. This further demonstrates that individuals give more donations to the charity that has raised less money when they see a relative difference in goal progress, but not when both charities have the same consistent levels of goal progress. This means that when the two charities have similar levels of goal progress (e.g., both are at 10% goal progress), participants are equally likely to donate to either charity and donate the same amount to each. However, when goal progress is different (e.g., 10% vs. 90%), people are more likely to donate to the charity that is relatively far from reaching its goal.

Donation intentions. A one-way ANOVA revealed a marginal difference in likelihood of donations to the charity farther from its goal among the control, relative-, and consistent-goal-progress conditions, F(2, 497) = 2.30, *p* = .10. Follow-up contrasts showed that participants were less likely to donate to the charity farther from its goal in the consistent-goal-progress condition (M = 4.16, SD = 2.00) than the control condition (M = 4.69, SD = 2.05), t(497) = 2.08, *p* = .04. This indicates that, in line with our predictions, likelihood of donating to a charity that has raised less money was not greater when absolute amounts raised were different but percentage goal progress was consistent, as shown in the figure below.

FIGURE W21: DONATION LIKELIHOOD WITH LESS VS. MORE FUNDS IN EACH CONDITION IN SS3

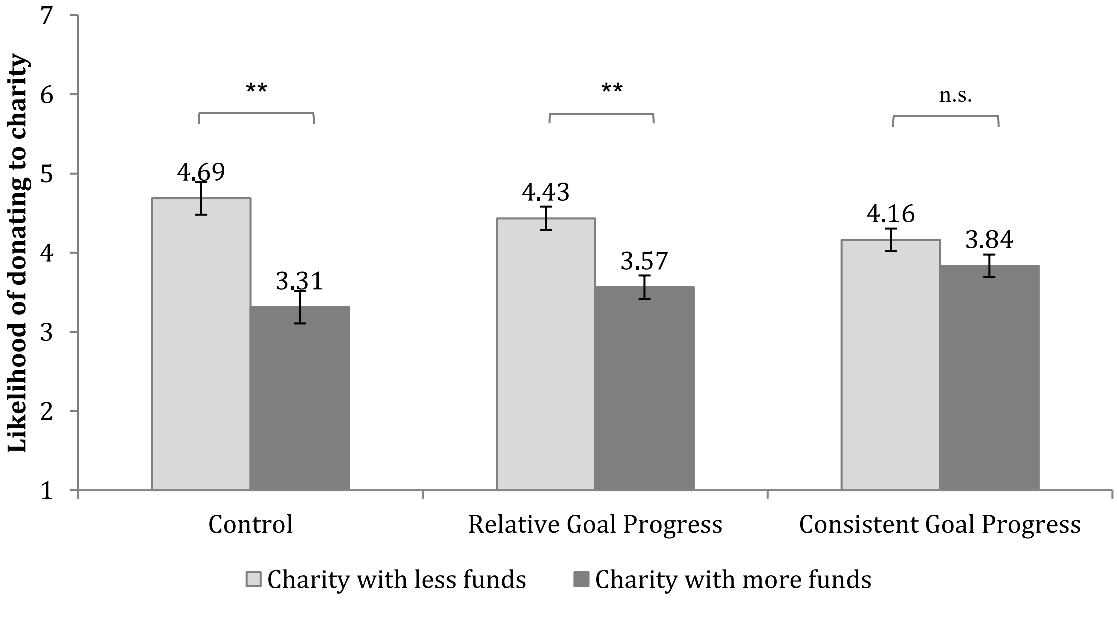

*Note: Error bars represent ±1 standard error. * $p < .05$, ** $p < .01$, *** $p < .001$*

Mediation. We carried out mediation analysis using PROCESS model 4 (Hayes 2013) with condition as a multi-categorical independent variable using the consistent-goal-progress condition as the baseline, need as the mediator, and amount given to the charity with less funds as the dependent variable. This revealed that the higher donations to charity were driven by higher perceptions of need in the control, B = 4.05, SE = 1.34, 95% CI [1.4901, 6.7701], and relative-goal-progress conditions, B = 4.31, SE = 1.15, 95% CI [2.2168, 6.6981], compared to the consistent-goal-progress condition.

Similarly, intentions to donate were driven by higher perceptions of need in the control, B = .43, SE = .14, 95% CI [.1769, .7238], and relative-goal-progress conditions, B = .46, SE = .11, 95% CI [.2515, .6926], compared to the consistent-goal-progress condition.

The indirect effect of need continued to be significant in a parallel mediation analysis including need, impact, competence, and warmth perceptions as mediators in the control, B = .24, SE = .08, 95% CI [.0862, .4035], and relative-progress conditions, B = .25, SE = .07, 95% CI [.1268, .4011], compared to the consistent-progress condition. There were two additional significant mediators when comparing the relative- and consistent-progress conditions; in the relative-progress condition, perceptions of higher impact for the charity farther from its goal led to greater likelihood of giving to this charity, B = .15, SE = .07, 95% CI [.0233, .2933]. Additionally, in the relative-progress condition, perceptions of lower competence for the charity farther from its goal led to lower likelihood of giving to this charity, B = -10, SE = .05, 95% CI [-.2110, -.0054]. No other effects were significant.

Parallel mediation results showed that participants perceived the charity farther from its goal as more in need in the control, B = 2.17, SE = .88, 95% CI [.7204, 4.1459], and relative-progress conditions, B = 2.32, SE = .87, 95% CI [.8660, 4.2824], compared to the consistent-progress condition, and that this drove donation amounts. In addition, perceptions of higher impact increased donations, B = 1.88, SE = .89, 95% CI [.2986, 3.7680], and perceptions of lower competence decreased donations, B = -.87, SE = .53, 95% CI [-2.0735, -.0362], in the relative- compared to the consistent-goal-progress conditions.

Discussion

This study replicates the results of previous studies with varying levels of progress and goal amounts and demonstrates that people are more willing to donate to the charity that has lower levels of goal progress compared to one with higher levels of goal progress. This study indicates that it is the percentage of progress towards the goal that determines the focal effect and not the absolute amount raised by the charity. Further, this supports the idea that participants judge levels of need in a relative manner, by comparing the progress made by the charities presented rather than looking only at absolute amounts. In addition, this study provide support for the mediating role of perceptions of relative need on donations to charity, and test the roles of impact, warmth, and competence perceptions.

Supplemental Study S7: To-date vs. To-go Framing

This study tests if the closing-the-gap effect is robust to framing. Previous work on the goal gradient effect has found that compared to to-date frames, to-go frames help increase the allocation of funds to an organization closer to its goal by shifting the standard of reference to the final goal and directing focus to the distance remaining to reach the goal (Bonezzi, Brendl, and De Angelis 2011; Wiebenga and Fennis 2014). In line with this work, we test whether the use of to-go frames attenuates the "closing the gap" effect in the charitable context by directing attention towards the small amount left to complete the goal for the charity closer to its goal. In this study we also enhance ecological validity in several ways. First, we use real charitable projects that serve distinct outcomes—i.e., providing water pumps versus providing vaccines . Second, we include projects that are part of the same overall organization and could therefore be promoted jointly or separately by a single organization raising money for different projects, thus enhancing the managerial relevance of our findings. Third, we provide participants with a bonus amount for participating in the study that they can either donate to one of the organizations or keep the full amount for themselves. Thus, this is a consequential decision for every participant.

Method

Participants and design. We recruited 801 participants (49.2% women, M_{age} = 38.78) from Prolific Academic to take part in this study. This study used a 3 (Evaluation type: Joint vs. Separate close to goal vs. Separate far from goal) x 2 (Framing: To-date vs. To-go) between-subjects design.

Procedure. Participants were informed at the start of the study that there would be an opportunity to donate to UNICEF and that they would be given 50 pence, in addition to their £0.50 payment for participation. They could use the money to donate to the project(s) they will see or keep it for themselves. Thus, participants could choose to keep the bonus or donate it, making this a consequential decision.

Participants were then shown either two charity projects or a single charity project from UNICEF. One of these projects was titled "Donate to buy a water pump" (adapted from previous research manipulating framing, Bonezzi et al. 2011). The second project was selected based on a pretest with 6 different charity projects asking participants to compare each project to donating to buy a water pump (see Web Appendix A for details of pretest). The project titled "Donate to buy vaccines" was rated closest to the water pump project in impact and neediness and was thus used in this study. This study thus used two projects that were for different, unrelated causes but part of the same organization.

In the joint evaluation condition, participants saw both projects with one closer to reaching its goal and the other farther from reaching its goal. The order (left vs right) and the names of the organizations were counterbalanced. In the separate evaluation condition, participants saw a single organization either closer to or farther from its goal. In the to-date frame condition, the amount raised so far was marked in grey, and the project farther from its goal had raised '£55 to-date' out of £300 and the project closer to its goal had raised '£245 to-date' out of £300. In the to-go frame condition the amount remaining to reach the goal was

marked in grey, and the project farther from its goal had '£245 to-go' to reach £300 and the one closer to its goal had '£55 to-go' to reach £300 (see Web Appendix A for stimuli).

Participants were then reminded that they had earned a bonus of £0.50 and that they could donate their bonus to the UNICEF project(s) they just saw or keep the money for themselves, in addition to their payment. Unlike previous studies, participants were not able to split their donation in this study and thus would have to give the full bonus they had earned if they decided to donate. Participants indicated their answer, and the money was later distributed to the causes and participants according to their answers after the study ended.

While our previous studies had measured participants' perceptions of need, this study aimed to explore their motivations for their decisions. Thus we asked participants to indicate the extent to which their decision was based on three factors; our proposed mediator need ("My decision was based on how much the organization was in need") along with fairness ("My decision was based on fairness"), and effort ("My decision was based on how much work was put into raising money") on scales ranging from 1(Strongly disagree) to 7(Strongly agree). They also rated the information they saw in terms of evaluability and justifiability with items taken from previous research (Li and Hsee 2019). For evaluability. participants saw questions reflecting the condition they were in (e.g., those in the farther from goal, to-date condition would answer "If an organization has £55 left to raise out of £300, do you have any idea how in need they are?", 1 - I don't have any idea and 7 – I have a clear idea) and justifiability ("When making donation decisions, do you think you should consider how much the organization is in need?", 1 - Should not consider and 7 - Should definitely consider). Lastly, participants completed an attention check asking them to select both always and never and answered basic demographic questions including age, gender, income, and ethnicity.

Results

Data Preparation. After excluding participants who failed the attention check (as per our pre-registered criteria), we were left with 781 participants (49.3% Women, M_{age} = 38.73).

Donation. A hybrid t-test showed that there was a preference reversal between joint and separate evaluations (collapsing across to-date and to-go frames), t(779) = 2.10, *p* = .04. A paired t-test revealed that, in joint evaluation, participants gave significantly more to the project further (M = .16) as opposed to closer (M = .11) to its goal, t(256) = 2.14, *p* = .03. Examining the to-date and to-go frames separately, this difference was significant in the to-date frame condition in JE (M_{far} = .16, M_{close} = .10), t(127) = 1.96, *p* = .05, consistent with previous studies. However, the difference in the to-go frame condition in JE was not significant (M_{far} = .16, M_{close} = .12), t(128) = 1.07, *p* = .29. Independent samples t-tests showed that, in separate evaluations, there was no difference in donations to the projects based on distance from goal (M_{far} = .25, M_{close} = .25), t(522) = .26, *p* = .79, neither in the to-date frame condition (M_{far} = .28, M_{close} = .24), t(260) = .74, *p* = .46, nor in the to-go frame condition (M_{far} = .23, M_{close} = .26), t(260) = 1.11, *p* = .27.

Next, we looked at the reasons behind participants decisions. Given the study included a binary choice as the dependent variable, traditional mediation analysis was not appropriate and instead we analyzed differences in motivation in joint and separate evaluations independently, in line with our pre-registration. When evaluating options jointly, participants indicated that need

was a stronger motivator than both fairness, t(253) = 3.00, $p = .003$, and effort, t(255) = 7.77, $p < .001$. Fairness was a stronger motivator than effort, t(252) = 6.73, $p < .001$. However, when evaluating options separately, there was no difference between the importance of need and fairness, t(516) = .17, $p = .86$, though both were stronger motivators than effort t(522) = 9.44, $p < .001$ and t(517) = 9.58, $p < .001$. Regression analyses with need, fairness and effort predicted donations to charity, $R^2 = .46$, F(3, 766) = 215.18, $p < .001$. Overall donations were predicted by need, B = .06, $p < .001$, and fairness, B = .02, $p < .001$, but not effort, B = .004, $p = .40$. These results indicate that, while fairness may play a role, it is a weaker predictor of donations than need, particularly in joint evaluations.

We then looked at evaluability and justifiability. Participants felt that it was equally easy to evaluate need for the charity further from its goal in joint vs. separate evaluations, in both the to-date frame, t(254) = .30, $p = .77$, and the to-go frame, t(255) = .71, $p = .48$. Similarly, participants felt that it was equally easy to evaluate need for the charity closer to its goal in joint vs. separate evaluations, in both the to-date frame, t(256) = 1.23, $p = .22$, and the to-go frame, t(257) = .51, $p = .61$. Lastly, need was seen as equally justifiable across conditions, F(2, 776) = .79, $p = .46$.

Discussion

This study provides further support for our prediction that consumers give more to a charitable project further from its goal compared to one closer to its goal in joint evaluation, but not in separate evaluation. This study also shows that the effect holds for consequential decisions involving allocating real money to the self versus a cause. Further, it provides empirical evidence that need is a stronger motivator of charitable giving than both fairness and effort. Importantly, evaluation mode did not alter perceptions of evaluability or justifiability, but does change participants' use of need as an important factor in decision making. Finally, this study suggests that a to-go frame may attenuate the basic effect, perhaps by shifting focus to the final goal.

REFERENCES

Aaker, Jennifer, Kathleen D. Vohs, and Cassie Mogilner (2010), "Nonprofits Are Seen as Warm and for-Profits as Competent: Firm Stereotypes Matter," *Journal of Consumer Research*, 37 (2), 224–37.

Anik, Lalin and Michael I. Norton (2020), "On Being the Tipping Point: Social Threshold Incentives Motivate Behavior," *Journal of the Association for Consumer Research*, 5 (1), 19–33.

Argo, Nichole, David Klinowski, Tamar Krishnamurti, and Sarah Smith (2020), "The Completion Effect in Charitable Crowdfunding," *Journal of Economic Behavior & Organization*, 172, 17–32.

Bonezzi, Andrea, C. Miguel Brendl, and Matteo De Angelis (2011), "Stuck in the Middle: The Psychophysics of Goal Pursuit," *Psychological Science*, 22 (5), 607–12.

Cryder, Cynthia E., George Loewenstein, and Howard Seltman (2013), "Goal Gradient in Helping Behavior," *Journal of Experimental Social Psychology*, 49 (6), 1078–83.

Fishbach, Ayelet, Marlone D. Henderson, and Minjung Koo (2011), "Pursuing goals with others: Group identification and motivation resulting from things done versus things left undone," *Journal of Experimental Psychology: General*, 140 (3), 520–34.

Fishbach, Ayelet, Minjung Koo, and Stacey R. Finkelstein (2014), "Chapter Five - Motivation Resulting from Completed and Missing Actions," in *Advances in Experimental Social Psychology*, J. M. Olson and M. P. Zanna, eds., New York: Academic Press, 257–307.

Fiske, Susan T., Amy JC Cuddy, and Peter Glick (2007), "Universal dimensions of social cognition: Warmth and competence," *Trends in Cognitive Sciences*, 11 (2), 77–83.

Grewal, Dhruv and Howard Marmorstein (1994), "Market Price Variation, Perceived Price Variation, and Consumers' Price Search Decisions for Durable Goods," *Journal of Consumer Research*, 21 (3), 453–60.

Grewal, Dhruv, Howard Marmorstein, and Arun Sharma (1996), "Communicating Price Information through Semantic Cues: The Moderating Effects of Situation and Discount Size," *Journal of Consumer Research*, 23 (2), 148.

Hayes, Andrew F. (2013), *Introduction to mediation, moderation, and conditional process analysis: a regression-based approach*, New York, NY: Guilford Press.

Jensen, Jakob D., Andy J. King, and Nick Carcioppolo (2013), "Driving Toward a Goal and the Goal-Gradient Hypothesis: The Impact of Goal Proximity on Compliance Rate, Donation Size, and Fatigue," *Journal of Applied Social Psychology*, 43 (9), 1881–95.

Koo, Minjung and Ayelet Fishbach (2008), "Dynamics of self-regulation: How (un)accomplished goal actions affect motivation.," *Journal of Personality and Social Psychology*, 94 (2), 183–95.

Koo, Minjung and Ayelet Fishbach (2012), "The Small-Area Hypothesis: Effects of Progress Monitoring on Goal Adherence," *Journal of Consumer Research*, 39 (3), 493–509.

Koo, Minjung and Ayelet Fishbach (2014), "Dynamics of self-regulation: How (un)accomplished goal actions affect motivation," *Motivation Science*, 1 (S), 73–90.

Li, Xilin and Christopher K. Hsee (2019), "Beyond Preference Reversal: Distinguishing Justifiability from Evaluability in Joint Versus Single Evaluations," *Organizational Behavior and Human Decision Processes*, 153, 63–74.

Montoya, Amanda K. and Andrew F. Hayes (2017), “Two-Condition Within-Participant Statistical Mediation Analysis: A Path-Analytic Framework,” *Psychological Methods*, 22 (1), 6–27.
Wash, Rick (2013), “The Value of Completing Crowdfunding Projects.,” *ICWSM*, 13, 2013.
Wiebenga, Jacob H. and Bob M. Fennis (2014), “The Road Traveled, the Road Ahead, or Simply on the Road? When Progress Framing Affects Motivation in Goal Pursuit,” *Journal of Consumer Psychology*, 24 (1), 49–62.
